# Clinical and Inflammatory Outcomes of Rotational Atherectomy in Calcified Coronary Lesions: A Systematic Review and Meta-Analysis

**DOI:** 10.3390/jcm14155389

**Published:** 2025-07-31

**Authors:** Az Hafid Nashar, Andriany Qanitha, Abdul Hakim Alkatiri, Muhammad Azka Alatsari, Nabilah Puteri Larassaphira, Rif’at Hanifah, Rasiha Rasiha, Nurul Qalby, Akhtar Fajar Muzakkir

**Affiliations:** 1 Pusat Jantung Terpadu (Makassar Cardiac Center), Dr. Wahidin Sudirohusodo General Teaching Hospital, Makassar 90245, South Sulawesi, Indonesia; azhafidn@unhas.ac.id (A.H.N.); ahalkatiri@unhas.ac.id (A.H.A.); afm@unhas.ac.id (A.F.M.); 2 Department of Cardiology and Vascular Medicine, Faculty of Medicine, Hasanuddin University, Makassar 90245, South Sulawesi, Indonesia; 3 Department of Physiology, Faculty of Medicine, Hasanuddin University, Makassar 90245, South Sulawesi, Indonesia; 4 Faculty of Medicine, Hasanuddin University, Makassar 90245, South Sulawesi, Indonesia; atsarimaa20c@student.unhas.ac.id (M.A.A.); larassaphiranp20c@student.unhas.ac.id (N.P.L.); hanifahr20c@student.unhas.ac.id (R.H.); rasiha20c@student.unhas.ac.id (R.R.); 5 Department of Cardiology, Heart and Lung Division, University Medical Center Utrecht, 3584 CX Utrecht, The Netherlands; n.qalby@umcutrecht.nl; 6 Department of Public Health and Community Medicine, Faculty of Medicine, Hasanuddin University, Makassar 90245, South Sulawesi, Indonesia

**Keywords:** rotational atherectomy, calcified coronary artery, inflammation, clinical outcomes, interleukin-6

## Abstract

**Objectives**: To assess the clinical and inflammatory outcomes of patients with calcified coronary arteries treated with rotational atherectomy (RA), compared to those with other intervention procedures. **Methods**: We conducted a systematic search of PubMed (Medline) and Embase. This review followed the PRISMA (Preferred Reporting Items for Systematic Reviews and Meta-Analyses) guidelines and applied the PICO criteria. **Results**: A total of 110 articles were analyzed, comprising 2,328,417 patients with moderate to severe coronary calcified lesions treated with RA, conventional percutaneous coronary intervention (PCI), or other advanced interventions. The pooled incidence of short- to mid-term major adverse cardiovascular events (MACEs) was 6% (95% CI 4–7%), increasing to 17% (95% CI 15–21%) at 6 months. Mortality was 2% (95% CI 1–3%) within 6 months, rising to 7% (95% CI 6–9%) thereafter. RA significantly increased the risk of long-term MACEs, mortality, total lesion revascularization (TLR), bleeding, and fluoroscopy time, and was borderline associated with an increased risk of short-term myocardial infarction and a reduced risk of coronary dissection. RA and other invasive procedures showed similar risks for short-term MACEs, mortality, total vascular revascularization (TVR), stent thrombosis, heart failure, stroke, and inflammation. **Conclusions**: RA is linked to higher long-term risks of MACEs, mortality, TLR, bleeding, and fluoroscopy time compared to other interventions. While RA shows comparable outcomes for short-term MACEs and mortality with other procedures, it may slightly reduce the risk of coronary dissection. These findings underscore the importance of careful patient selection and weighing long-term risks when considering RA for calcified coronary lesions.

## 1. Introduction

Moderate to severe coronary artery calcification affects nearly one-third of patients undergoing percutaneous coronary intervention (PCI) [1,2]. Calcified lesions present critical barriers during PCI, including challenges in device delivery, inadequate stent deployment, and a high incidence of procedural complications [3,4]. These issues can lead to poor clinical outcomes, such as restenosis and stent thrombosis [5]. Various calcium modification techniques have emerged to tackle these challenges, including rotational, orbital, and laser atherectomy, as well as shockwave lithoplasty [3,6]. The optimal approach for treating calcified lesions often involves combining enhanced intravascular imaging with appropriate plaque modification tools to ensure adequate lesion preparation and optimal stent deployment [7].

Rotational atherectomy (RA) still serves as a fundamental element in the management of heavily calcified coronary lesions, particularly in the drug-eluting stent (DES) era. Recent evidence has provided a clearer comparison between RA and other calcium modification techniques. The DIRO randomized trial showed that RA achieved better stent expansion and greater tissue modification than orbital atherectomy (OA), with comparable clinical outcomes [8]. A retrospective analysis of left main disease further proved similar short-term outcomes between RA and OA, though OA was linked to higher rates of perforation and dissection [9]. A systematic review also showed no clear advantage of OA or intravascular lithotripsy (IVL) over RA in terms of long-term major adverse cardiovascular events (MACEs), while IVL showed procedural benefits in selected cases [10]. Comparative studies suggest IVL may better maintain microvascular function post-PCI [11], but with higher lesion selectivity and limited data in complex disease [12]. These findings strengthen the continued clinical relevance of RA in complex PCI.

RA has evolved from a plaque debulking technique to a lesion modification strategy for treating calcified coronary lesions, particularly in the drug-eluting stent (DES) era [13,14]. It is primarily used to modify heavily calcified lesions, facilitating balloon dilation and stent deployment [14,15]. Indications for RA have advanced to include diffuse atheromatous disease, in-stent restenosis, and chronic total occlusions [16]. While RA increases procedural success in calcified lesions, its impact on long-term outcomes remains debatable [17,18]. Some studies report excellent mid-term outcomes with aggressive plaque modification before DES implantation [19], while others show no reduction in late lumen loss [18]. Despite these mixed results, RA remains a valuable tool in the treatment of complex calcified lesions, evolving from debulking to plaque modification over the past 40 years [20]. Current guidelines from the ACC/AHA and ESC provide limited recommendations on RA use [21]. Although RA does not reduce restenosis rates, it remains a valuable tool for treating complex calcified lesions in the drug-eluting stent era [14,22].

RA in PCI induced an inflammatory response, which can affect clinical outcomes. C-reactive protein (CRP) and interleukin-6 (IL-6) are key inflammatory markers associated with cardiovascular risk and restenosis after procedures [23]. These markers have been demonstrated to predict clinical restenosis and MACEs following interventions. This meta-analysis aimed to update and quantify the incidence rate of composite MACEs and mortality following RA, and to compare the impact of RA with other invasive strategies on clinical outcomes (e.g., intra- and post-procedural complications, composite MACEs) and inflammatory responses (e.g., CRP, IL-6) in patients undergoing coronary interventions for moderate to severe coronary artery calcification.

## 2. Materials and Methods

### 2.1. Data Sources

Clinical studies evaluating the safety and effectiveness of RA in patients undergoing PCI for moderate to severe calcified coronary artery disease were identified through a search of electronic databases, including PubMed and EMBASE, covering publications up to February 2025. The detailed PICO of this systematic review is presented in Table 1.

The search strategy included a variety of terms related to “rotational atherectomy”, “rotablation”, “rotational”, “PCI”, and “atherectomy”. Detailed descriptions of the search strategies for both PubMed and EMBASE are provided in Table S1. This systematic review and meta-analysis were conducted in accordance with the PRISMA (Preferred Reporting Items for Systematic Reviews and Meta-Analyses) guidelines, as detailed in Table S2. This systematic review and meta-analysis was registered on the International Platform of Registered Systematic Reviews and Meta-Analysis Protocols (INPLASY) with the protocol number INPLASY202530044 (https://doi.org/10.37766/inplasy2025.3.0044).

### 2.2. Study Selection and End Points

Three authors (A.Q., A.Z.A., and N.P.L.) independently screened all titles and abstracts. Articles were selected based on the following inclusion criteria: (a) the study reported RA only or compared RA with Standard PCI or other debulking atherectomy methods (e.g., orbital atherectomy, direct atherectomy, laser, or intracoronary lithotripsy); (b) clinical trials, cohort studies, and case–control studies; (c) studies reporting adverse clinical outcomes, such as post-procedural complications (e.g., coronary artery dissection, device-induced arterial perforation, cardiac tamponade), slow flow/no reflow, MI, emergency CABG, stroke, in-stent restenosis, stent thrombosis, target vessel revascularization (TVR), target lesion revascularization (TLR), mortality, and composite MACEs; or studies reporting inflammatory markers (e.g., CRP, IL-6, TNF-alpha) pre- and post-procedure.

Studies that did not provide clear inflammatory or clinical outcome measures, did not focus on RA, or were animal and laboratory studies were excluded. Any disagreements among the authors were resolved through consensus or discussion. MACEs were defined based on the criteria used in each study and typically included myocardial infarction, all-cause mortality, target vessel revascularization (TVR), target lesion revascularization (TLR), emergency coronary artery bypass grafting (CABG), stent restenosis, or stent thrombosis. Short- and mid-term outcomes referred to events occurring during hospitalization or within 6 months post-procedure, while long-term outcomes were defined as adverse events occurring more than 6 months after the index procedure. In cases where definitions of clinical endpoints—particularly MACEs and inflammatory markers—varied across studies, we adopted the definitions provided by the original authors and documented the corresponding criteria in our data extraction table (Table S3). When pooling data, we made efforts to harmonize outcome categories where clinically appropriate and conducted sensitivity analyses when necessary to account for discrepancies in definitions.

### 2.3. Data Extraction and Quality Assessment

**Figure 1:**
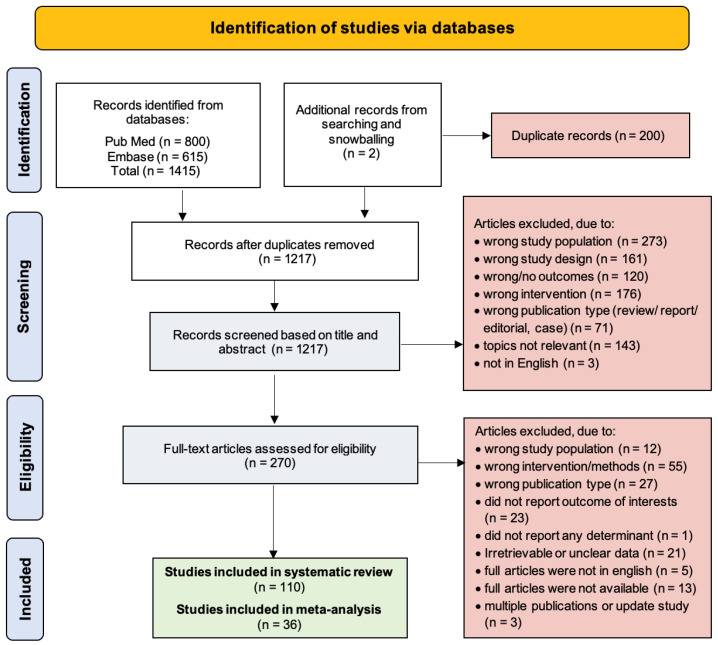
PRISMA flow chart of the study selection.

Titles and abstracts of all relevant studies were imported into the Rayyan Intelligent Systematic Review platform (https://rayyan.ai, last accessed on 30 November 2024), where duplicate entries were removed. Studies were selected followed to the PRISMA flow chart, as shown in Figure 1. Data from all eligible studies were extracted into a standardized dataset by AQ, MAA, NPL, RH, and RR. Extracted information included the first author, publication year, study design, sample size and demographic characteristics, duration of follow-up, coronary artery disease (CAD) risk factors, type of intervention (e.g., RA alone or in comparison with other techniques), reported outcomes, incidence of adverse events, and key findings, which are summarized in Table S3. The extracted data were reviewed and validated by other authors (A.Q., A.H.A., A.H.N., and A.F.M.).

The quality of included observational studies was assessed using the Newcastle–Ottawa Scale (NOS), while RCTs were evaluated using the Risk of Bias (RoB) tool in Review Manager (RevMan ver. 5.4 for Mac). The NOS grades each study on three criteria: study group selection (maximum of four stars), comparability of the groups (maximum of two stars), and outcome assessment (maximum of three stars). The detailed risk of bias for each observational cohort study is shown in Table 2. For RCTs, the RoB 2 tool assesses five domains: bias from the randomization process, deviations from intended interventions, missing outcome data, outcome measurement, and selection of reported results. Three independent reviewers (N.P.L., R.H., and M.A.A.) conducted the risk of bias evaluations using these tools. Any disagreements were resolved by consensus.

### 2.4. Statistical Analysis

**Figure 2:**
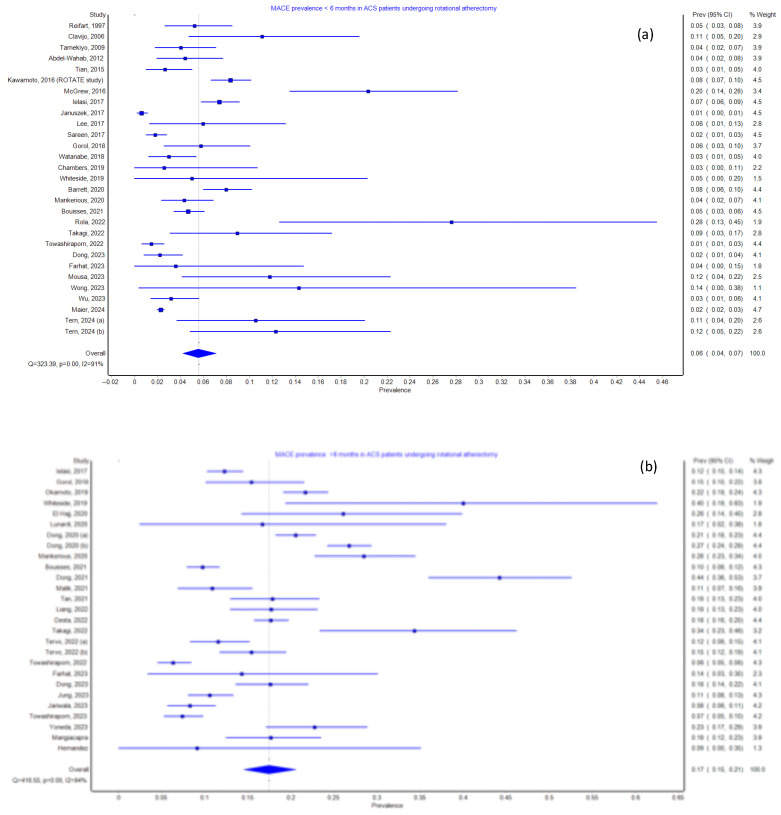

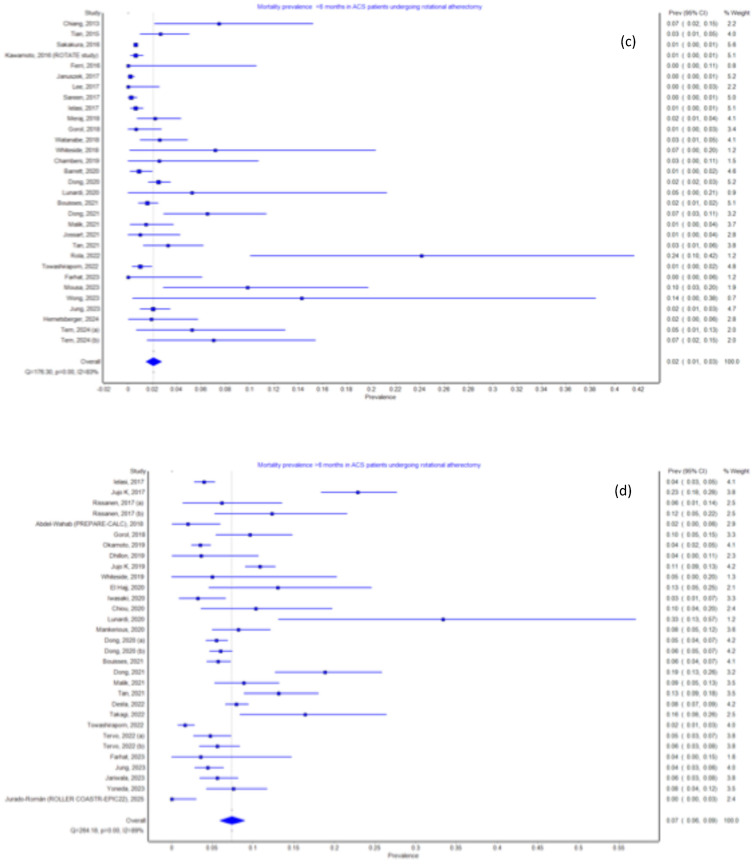
Incidence rates of: (**a**) short- and mid-term MACEs; and (**b**) long-term MACEs; (**c**) short- and mid-term mortality; and (**d**) long-term mortality.

Categorical data were presented as frequencies (*n* [%]), while continuous data were reported as means ± standard deviation (SD), or medians (Q1–Q3). Pooled event rates for short- and mid-term MACEs, long-term MACEs, as well as short-, mid-, and long-term mortality, along with their 95% confidence intervals (CIs), were calculated (Figure 2a–d). Meta-analyses were conducted using a random-effects model across the included studies. Relative risk (RR) estimates for dichotomous variables and mean differences (MDs) for continuous variables, along with their corresponding 95% CIs, were synthesized to examine the effect of RA on inflammatory and adverse clinical outcomes. Heterogeneity between studies was examined using the Chi-squared test and calculated using the I^2^ statistic. The degree of heterogeneity was classified as low (I^2^ < 25%), moderate (I^2^ = 25–49%), and substantial (I^2^ > 50%). To assess potential publication bias, we used a funnel plot and Egger’s regression test, with a *p*-value < 0.05 considered statistically significant. All statistical analyses were performed using Review Manager (RevMan) version 5.4, meta-Excel for Windows, and SPSS version 29 for Mac.

## 3. Results

### 3.1. Characteristics of Includes Studies

An initial search yielded 1415 articles. After removing duplicates and performing a snowballing search, the number was reduced to 1217. Title and abstract screening was then conducted, leading to the selection of 270 full-text articles for eligibility assessment. Ultimately, 110 studies fulfilled the inclusion criteria for the systematic review, with 36 subsequently included in the meta-analysis.

Our meta-analysis sourced the data from 2,328,417 patients across 36 studies (14 randomized controlled trials and 22 observational studies), all of which involved CAD patients with moderately to severely calcified coronary lesions who underwent RA, compared to those receiving a non-RA invasive strategy.

In this meta-analysis, we compared RA and other invasive strategies, including conventional percutaneous coronary intervention (PCI, *n* = 10), modified balloon angioplasty (cutting or scoring balloon, *n* = 9), Percutaneous Transluminal Coronary Angioplasty (PTCA, *n* = 9), intravascular lithotripsy (IVL, *n* = 6), Excimer Laser Coronary Angioplasty (ELCA, *n* = 2), orbital atherectomy (OA, *n* = 10), and Transmyocardial Laser Revascularization (TMLR, *n* = 1).

The mean age of participants ranged between 51.3 and 83.1 years, with the majority being male, accounting for 50% to 98.7% of the study population. Hypertension was the predominant risk factor, with its incidence ranging from 50% to 100% of participants. Table S3 provides a comprehensive overview of the participants’ characteristics, associated risk factors, and event rates reported in the included studies.

Table 2 presents the quality assessment of the observational studies included in the systematic review. Of the 92 observational studies evaluated for risk of bias (RoB), the median Newcastle–Ottawa Scale (NOS) score was 7.1 ± 1.3, with 63 studies (68.5%) rated as “good,” indicating a low risk of bias.

### 3.2. Incidence Rate of MACEs and Mortality on Rotational Atherectomy (RA)

We estimated the pooled incidence rates of short-, mid-, and long-term major MACEs following RA. The pooled incidence rate for short- and mid-term MACEs was 6% (95% CI 4–7%), increasing to 17% (95% CI 15–21%) beyond 6 months after the RA procedure (Figure 2a,b). The pooled mortality rate for patients undergoing RA was 2% (95% CI 1–3%) within 30 days and 6 months post-procedure, increasing to 7% (95% CI 6–9%) beyond 6 months following RA (Figure 2c,d).

### 3.3. Meta-Analysis

We illustrate the incidence rates of short- and mid-term (<6 months), as well as long-term (>6 months) composite MACEs and mortality among the study participants. We estimated short- and mid-term MACEs from a total of 31 studies, and the other 31 studies were used to quantify long-term mortality. Meanwhile, 29 studies were used for short- and mid-term MACEs, and 27 studies for long-term MACEs.

**Figure 3:**
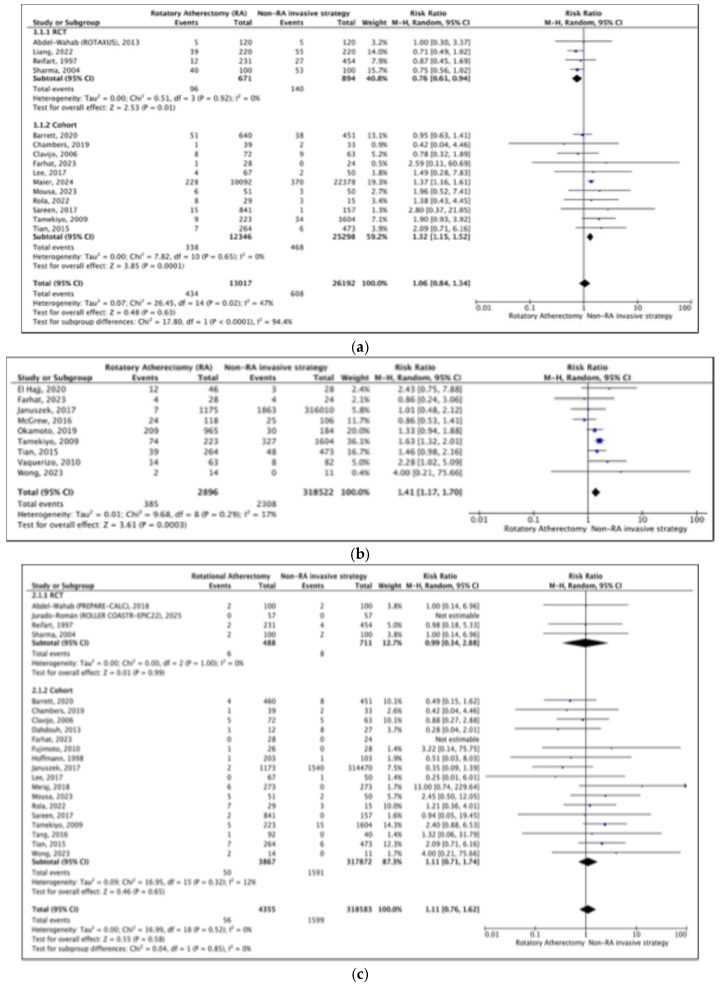

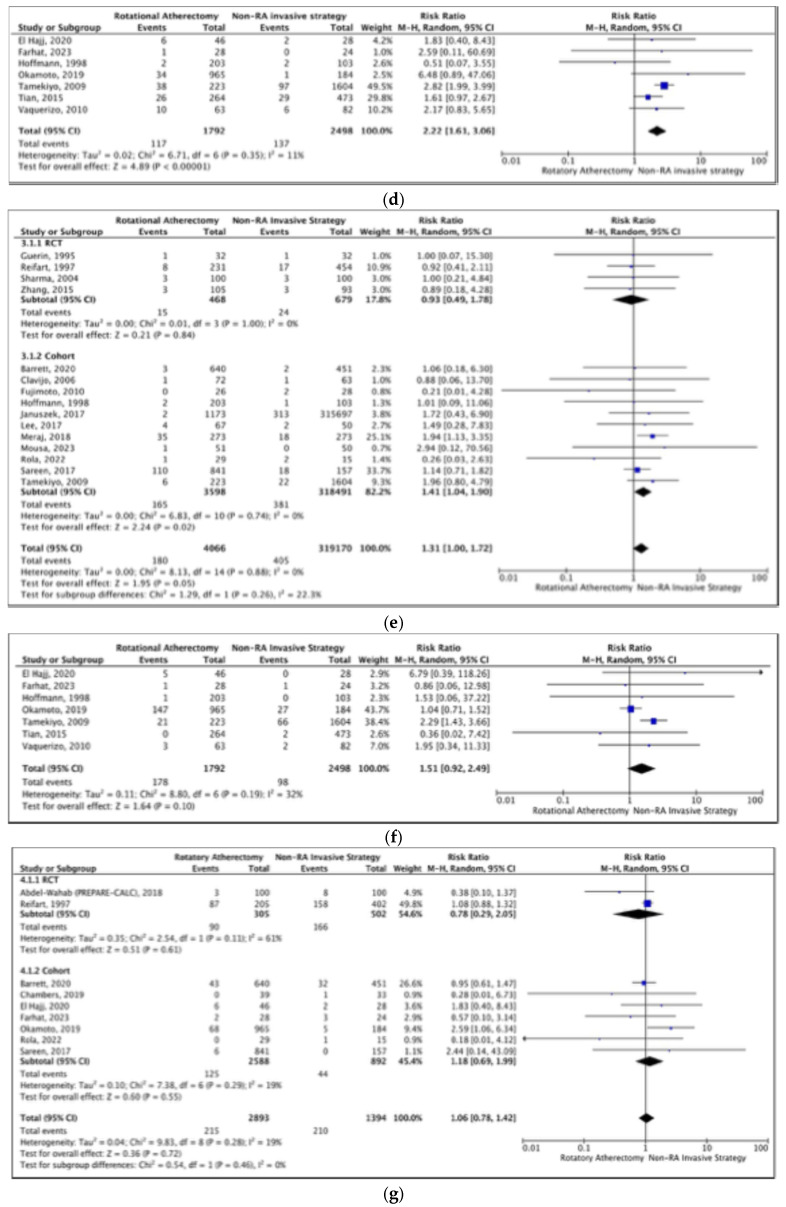

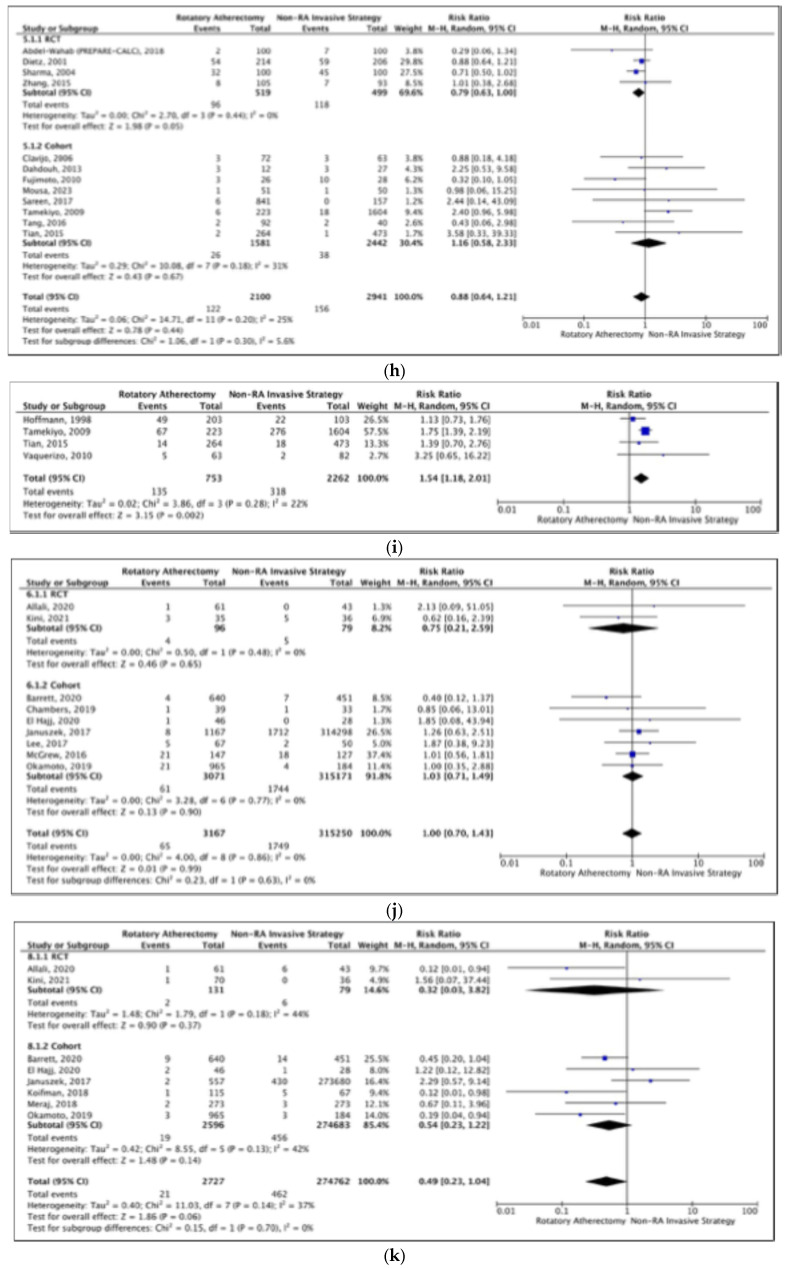

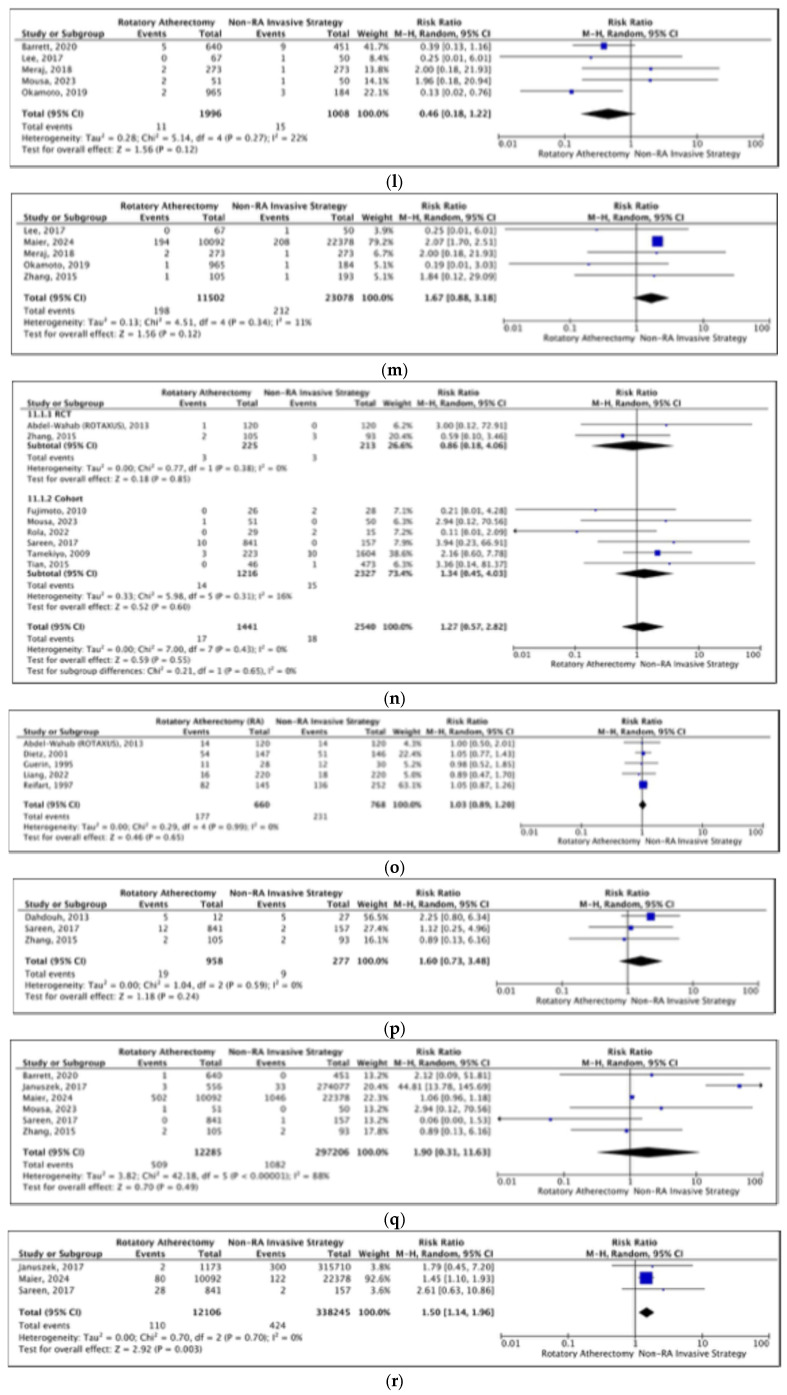

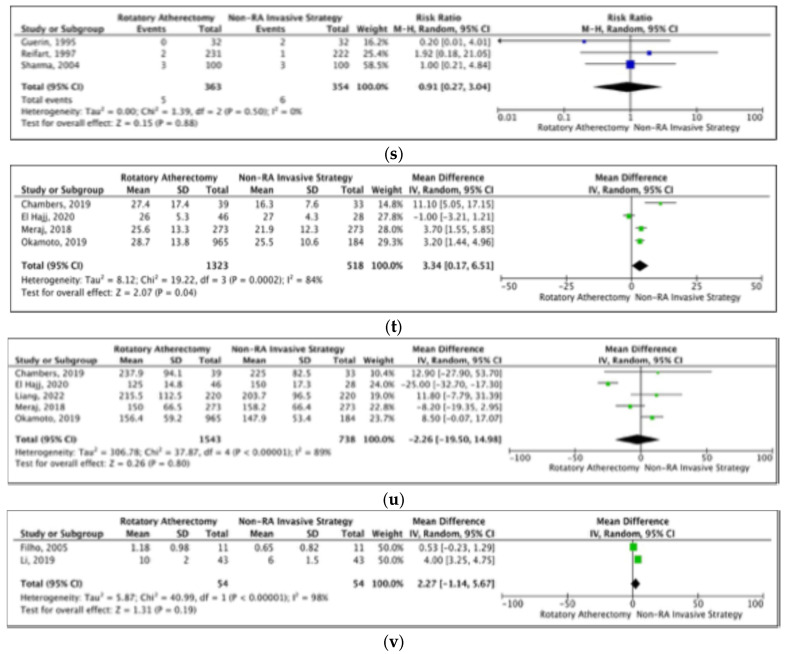
(**a**) Impact of RA vs. non-RA invasive strategies on short- and mid-term MACEs; (**b**) Impact of RA vs. non-RA invasive strategies on long-term MACEs; (**c**) Impact of RA vs. non-RA invasive strategies on short- and mid-term mortality; (**d**) Impact of RA vs. non-RA invasive strategies on long-term mortality; (**e**) Impact of RA vs. non-RA invasive strategies on short- and mid-term MI; (**f**) Impact of RA vs. non-RA invasive strategies on long-term MI; (**g**) Impact of RA vs. non-RA invasive strategies on Total Vascular Revascularization (TVR); (**h**) Impact of RA vs. non-RA invasive strategies on total lesion revascularization; (**i**) Impact of RA vs. non-RA invasive strategies on long-term TLR; (**j**) Impact of RA vs. non-RA invasive strategies on slow/no flow (TIMI flow < 3); (**k**) Impact of RA vs. non-RA invasive strategies on coronary dissection; (**l**) Impact of RA vs. non-RA invasive strategies on coronary perforation; (**m**) Impact of RA vs. non-RA invasive strategies on cardiac tamponade or effusion; (**n**) Impact of RA vs. non-RA invasive strategies on stent thrombosis; (**o**) Impact of RA vs. non-RA invasive strategies on in-stent restenosis; (**p**) Impact of RA vs. non-RA invasive strategies on heart failure (NYHA IV); (**q**) Impact of RA vs. non-RA invasive strategies on stroke; (**r**) Impact of RA vs. non-RA invasive strategies on bleeding; (**s**) Impact of RA vs. non-RA invasive strategies on emergency coronary artery bypass grafting (CABG); (**t**) Impact of RA vs. non-RA invasive strategies on fluoroscopy time; (**u**) Impact of RA vs. non-RA invasive strategies on contrast volume; (**v**) Impact of RA vs. non-RA invasive strategies on inflammatory marker (IL-6).

Meta-analyses were performed to assess the effect of RA on various adverse clinical outcomes, including (1) composite MACEs, (2) mortality, (3) myocardial infarction (MI), (4) total vascular revascularization (TVR), (5) total lesion revascularization (TLR), (6) slow/no flow (TIMI flow < 3), (7) coronary dissection, (8) coronary perforation, (9) cardiac tamponade or effusion, (10) stent thrombosis, (11) in-stent restenosis, (12) heart failure NYHA IV, (13) stroke, (14) bleeding, (15) emergency coronary artery bypass grafting (CABG), (16) fluoroscopy time, and (17) contrast volume. In addition, the effect of RA on inflammatory outcomes (i.e., IL-6) was also analyzed (Figure 3a–v). Detailed outcomes of interest and the studies included in the meta-analysis are provided in Table 3.

### 3.4. Outcomes of Rotational Atherectomy vs. Non-RA Invasive Strategy

#### 3.4.1. Composite MACEs

##### Short- and Mid-Term MACEs (≤6 Months)

The meta-analysis included four randomized controlled trials and eleven cohort studies that compared the effects of RA and non-RA invasive strategies on short- and mid-term MACEs. The findings from RCTs and observational cohort studies were contradictory. RCTs demonstrated that RA reduced the risk of short- and mid-term MACEs by 24%, while observational cohort studies showed an increase in risk by 32%. However, the pooled risk ratio (RR) showed no statistically significant difference between patients treated with RA versus those receiving non-RA strategies on short- and mid-term MACEs, with an RR of 1.06 (0.84–1.34). Among the 15 studies reporting these outcomes, moderate heterogeneity was observed (I^2^ = 47%, *p* = 0.02).

##### Long-Term MACEs (>6 Months)

Meanwhile, for long-term MACEs, the pooled analysis of 9 cohort studies demonstrated that RA significantly increased the risk by 41% compared to the non-RA invasive strategy (RR 1.41 [95% CI 1.17–1.70], *p* = 0.0003).

#### 3.4.2. Mortality

##### Short- and Mid-Term Mortality (≤6 Months)

We then analyzed the effect of RA on short- and mid-term MACEs, resulting in a pooled RR of 1.11 (0.76–1.62), indicating no significant difference between RA and other invasive strategies in affecting MACEs within 6 months after the procedure. Low heterogeneity was detected among the 21 studies included in the analysis.

##### Long-Term Mortality (>6 Months)

Our analysis identified a significant association between the RA procedure and an increased risk of long-term mortality compared to other invasive strategies. The pooled RR was 2.22 (95% CI 1.61–3.06), suggesting a two-fold higher risk of death after 6 months in patients who underwent the RA procedure. This result was consistent across studies, with low heterogeneity observed (I^2^ = 11%, *p* = 0.35).

#### 3.4.3. Myocardial Infarction (MI)

##### Short- and Mid-Term MI (≤6 Months)

Short- and mid-term MI outcomes following RA were reported in 15 of the studies included in our analysis. The results indicated a borderline significant association between RA and the occurrence of MI within six months, with a pooled RR of 1.31 (95% CI 1.00–1.72, *p* = 0.05), along with low heterogeneity (I^2^ = 22.3%, *p* = 0.26).

##### Long-Term MI (>6 Months)

When comparing RA and non-RA procedures in predicting long-term MI among patients with heavily calcified coronary lesions, we found no statistically significant difference between the groups. The pooled risk ratio was 1.51 (95% CI 0.92–2.49), suggesting a comparable incidence of long-term MI between the two groups. However, moderate heterogeneity was observed across the included studies (I^2^ = 32%, *p* = 0.19).

#### 3.4.4. Total Vascular Revascularization (TVR)

The RA and non-RA procedures likewise demonstrated a similar effect on the incidence of total vascular resistance after the procedure, with a pooled RR of 1.06 (95% CI 0.78–1.42) derived from both RCTs and cohort studies. Our analysis indicated low heterogeneity among the included studies (I^2^ = 19%, *p* = 0.28).

#### 3.4.5. Total Lesion Revascularization (TLR)

##### Short- and Mid-Term TLR (≤6 Months)

The next important outcome was total lesion revascularization (TLR). In four RCTs, we found that RA was borderline significantly associated with a lower risk of short-term TLR, with a pooled RR of 0.79 (95% CI 0.63–1.00). However, observational cohort studies revealed no significant difference between the RA and non-RA groups in terms of short-term TLR, with moderate heterogeneity (I^2^ = 25%, *p* = 0.20) observed across the 12 included studies.

##### Long-Term TLR (>6 Months)

Conversely, RA was found to significantly increase the risk of long-term TLR compared to non-RA procedures. The pooled RR indicated that patients who underwent RA had a 54% increased likelihood of experiencing TLR beyond 6 months compared to those who underwent non-RA procedures. The corresponding forest plot illustrates this association, with the analysis showing moderate heterogeneity among the included studies (I^2^ = 22%).

#### 3.4.6. Slow/No Flow (TIMI Flow < 3)

Slow or no flow was frequently reported as an adverse event in RA and advanced revascularization procedures. In the corresponding forest plot, a pooled analysis of nine studies revealed no significant difference in the risk of slow/no flow between RA and other invasive techniques, indicated by a risk ratio estimated at 1.00 (95% CI 0.70–1.43).

#### 3.4.7. Coronary Dissection

The next crucial assessment was to compare the incidence of intra-procedural coronary dissection between RA and other invasive procedures. A borderline significant association was found, showing RA had a lower risk of coronary dissection compared to non-RA procedures, with an RR of 0.49 (95% CI 0.23–1.04). However, moderate heterogeneity was noted among the studies (I^2^ = 37%, *p* = 0.14).

#### 3.4.8. Coronary Perforation

Likewise, the trend suggested a lower, though not statistically significant, risk of coronary perforation in the RA group compared to other invasive strategies, with a pooled RR of 0.46 (95% CI 0.18–1.22, *p* = 0.12). This outcome was reported in five studies, with low heterogeneity (I^2^ = 22%, *p* = 0.27).

#### 3.4.9. Cardiac Tamponade or Effusion

Moreover, RA and other invasive procedures showed similar effects in terms of cardiac tamponade or cardiac effusion, with a pooled RR of 1.67 (95% CI 0.88–3.18), with included studies showing low heterogeneity (I^2^ = 11%, *p* = 0.34).

#### 3.4.10. Stent Thrombosis

Both RCTs and cohort comparisons showed similar risks of stent thrombosis between the RA and non-RA groups, with a pooled RR of 1.27 (95% CI 0.57–2.82), *p* = 0.55.

#### 3.4.11. In-Stent Restenosis

Analysis from five RCTs showed that RA and other invasive procedures shared an equal risk of in-stent restenosis, with a pooled RR of 1.03 (95% CI 0.89–1.20) and no heterogeneity.

#### 3.4.12. Heart Failure NYHA IV

The incidence of Heart Failure (NYHA class IV) was another important outcome assessed in this study. Data from three studies were pooled, resulting in an RR of 1.60 (95% CI 0.73–3.48), suggesting no significant difference in heart failure occurrence between RA and other invasive treatment approaches.

#### 3.4.13. Stroke

Both RA and non-RA groups had similar risk in the incidence of stroke after the procedure, with a pooled RR of 1.90 (95% CI 0.31–11.63). However, this finding should be interpreted cautiously due to the high heterogeneity observed among the included studies, with an I^2^ value of 88% (*p* < 0.00001).

#### 3.4.14. Bleeding

In this forest plot, the pooled RR of 1.50 (95% CI 1.14–1.96) indicated that RA increased the risk of bleeding compared to other invasive procedures. Although only three studies reported this predictor, no heterogeneity was observed between studies.

#### 3.4.15. Emergency Coronary Artery Bypass Grafting (CABG)

Three studies reported emergency CABG as the outcome of interest. From the analysis, we found an equal risk of CABG between RA and non-RA invasive procedures, with a pooled RR of 0.91 (95% CI 0.27–3.04), with no heterogeneity observed across the included studies.

#### 3.4.16. Fluoroscopy Time

The pooled analysis of four studies demonstrated that RA patients spent more time under fluoroscopy (*p* = 0.04), with a mean difference of 3.34 min (95% CI 0.17–6.51), compared to those undergoing other invasive procedures. However, this finding should be interpreted with caution due to the high heterogeneity observed among the included studies, with an I^2^ value of 84% (*p* = 0.0002).

#### 3.4.17. Contrast Volume

No significant difference was observed in the volume of contrast used during RA and other invasive strategies, with high heterogeneity across studies (I^2^ = 89%).

#### 3.4.18. Inflammatory Marker: Interleukin-6

Finally, no significant difference was observed in inflammatory markers (IL-6) between RA and other invasive strategies. However, only two studies reported this outcome and therefore showed a high heterogeneity (I^2^ = 98%, *p*< 0.0001).

### 3.5. Publication Bias

**Figure 4:**
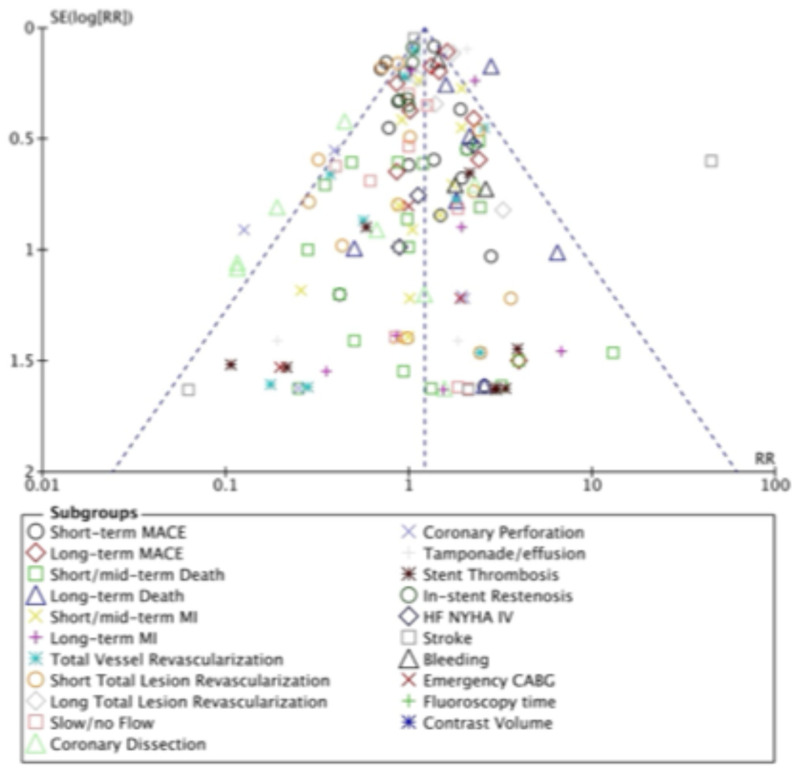
Funnel plot of the included studies in this meta-analysis.

We employed a funnel plot to visually assess potential publication bias across studies. Although there is a possibility of underreporting studies with lower risk ratios and small sample sizes, we observed a symmetric funnel plot, with studies ranging from large to small sample sizes, evenly distributed for all outcomes of interest, as described in Figure 4.

**Figure 5:**
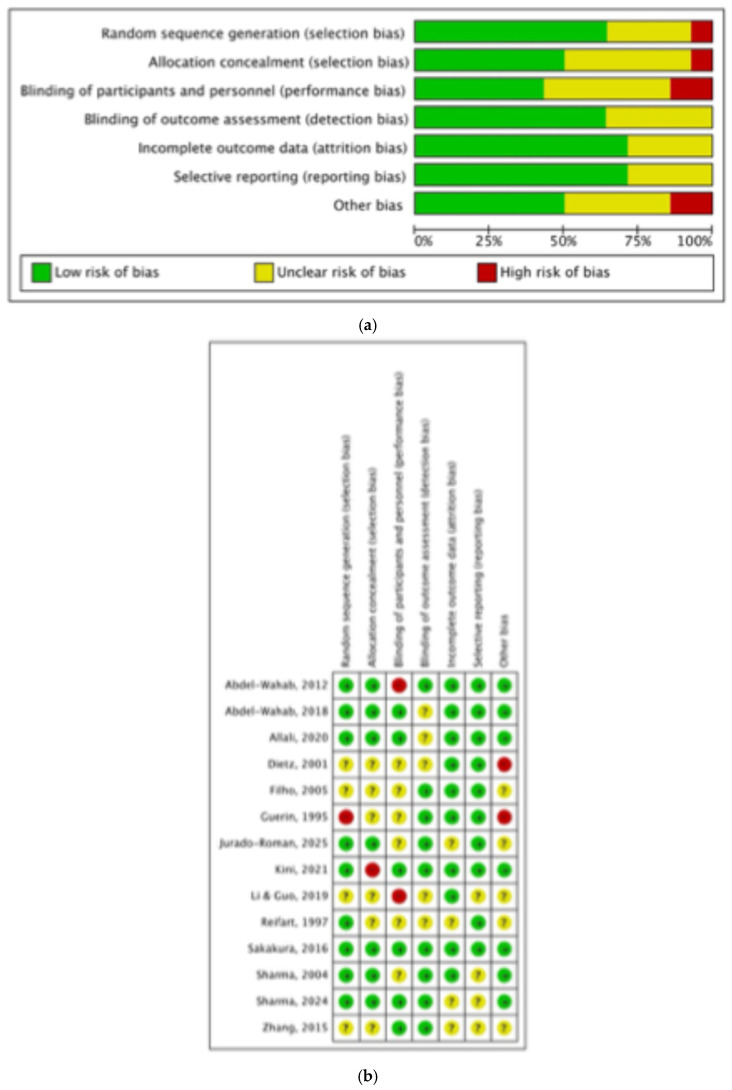
(**a**) Risk of Bias of RCTs included in meta-analysis (*n* = 14); (**b**) Summary of Risk of Bias (RoB) assessment for each included RCT.

Among the 14 RCTs, a significant proportion exhibited a low risk of bias. However, the domains of participant and personnel blinding showed the highest risk (12.5%), as illustrated in Figure 5a. A detailed RoB assessment for each RCT is presented in Figure 5b.

## 4. Discussion

Rotatory atherectomy (RA) is an effective technique for treating heavily calcified coronary lesions, with high procedural success rates [13,24,25]. However, long-term outcomes reveal high rates of MACEs following RA [26]. This is consistent with our findings, where we observed an incidence rate of MACEs ranging from 6% to 17%, and an incidence rate of mortality ranging from 2% to 7%.

Based on four RCTs, we found that RA significantly reduced the incidence of short-term MACEs by 24% compared to other invasive strategies. In contrast, analysis of observational studies suggested that RA increased the risk of short-term MACEs by 32%. Overall, the pooled relative risk (RR) for short-term MACEs indicated comparable risks between RA and other invasive strategies. From this meta-analysis, we found that RA significantly increased the risk of long-term MACEs, mortality, TLR, bleeding, and longer fluoroscopy time, compared to conventional PCI, PTCA, and other atherectomy techniques. Additionally, RA was borderline significantly associated with an increased risk of short-term MI and a borderline reduction in the risk of coronary dissection. Furthermore, we found that RA and other invasive procedures had similar risks for short-term MACEs, mortality, TVR, slow/no flow, stent thrombosis, in-stent restenosis, heart failure, stroke, emergency CABG, contrast volume, and inflammation (i.e., Interleukin-6).

Factors associated with increased risk of MACEs after invasive interventions include hemodialysis, advanced age, high EuroSCORE II, and longer stent length [25,27]. Compared to other atherectomy techniques, RA was not inferior to intravascular lithotripsy (IVL) in terms of stent expansion and cardiovascular outcome, but had shorter fluoroscopy times [28,29]. Comparative studies suggest orbital atherectomy may have lower mortality rates than RA [30]. Other complications that might occur when using RA, including no/slow flow, coronary dissection, coronary perforation, and burr entrapment [31,32]. To address these challenges, it is recommended to develop optimal RA techniques and combine them with other approaches.

Furthermore, the evaluation of inducible ischemia plays a critical role in guiding the patient-selection decision to perform invasive procedures such as RA. Non-invasive stress testing modalities, including stress echocardiography, myocardial perfusion imaging (SPECT), and cardiac MRI, are commonly used to assess inducible myocardial ischemia in patients prior to RA [33]. Procedural assessments, such as fractional flow reserve (FFR) or instantaneous wave-free ratio (iFR), provide objective functional data during angiography, helping to determine the hemodynamic significance of intermediate lesions [34]. The presence of inducible ischemia has been linked to worse prognosis and increased MACEs [35], underscoring the importance of integrating functional ischemia assessment prior to RA. Contemporary RA practice involves the use of smaller burr sizes, shorter ablation runs, and lower rotational speeds to enhance safety and reduce complications [22,36]. Although not routinely recommended, RA facilitates stent delivery and expansion in severely calcified lesions [37]. Intravascular imaging is employed to guide burr size selection and assess calcification thickness [32]. Although RA is effective, its use has been constrained by factors like high cost and concerns regarding potential complications.

RA is generally indicated for the management of severe calcified lesions, bulky plaques, or lesions that pose challenges for stent placement. It is important to note that RA is not advised for mild to moderately calcified lesions, as its use in such cases may lead to greater tissue damage and a higher risk of neointimal hyperplasia [14]. Indications for this plaque modification technique including diffuse atheromatous disease requiring the placement of long stents, diffuse in-stent restenosis, chronic total occlusion, and calcified ostial lesions [38]. In patients with de novo calcified lesions who clinically require PCIs, lesion modification using rotational or orbital atherectomy is recommended to improve procedural success when calcification is severe. If the severity of calcification is intermediate or unclear based on angiography, intravascular imaging techniques such as intravascular ultrasound (IVUS) or optical coherence tomography (OCT) can be helpful for more accurate assessment and classification [22].

For severely calcified lesions, using modified balloons after RA may reduce MACE rates compared to plain balloon angioplasty [39]. Utilizing intravascular imaging tools like IVUS and OCT can enhance the safety and effectiveness of RA by reducing adverse events. IVUS, in particular, provides insight into guidewire bias, which is essential for selecting the proper burr size during the procedure. In addition, IVUS remains functional even if dissection or hematoma occurs following RA. OCT offers precise insights into calcified lesions, including measurements of calcification thickness [32].

The essential elements of an optimal technique of RA include maintaining the burr-to-artery ratio between 0.4 and 0.6, an ablation speed of 135,000 to 180,000 rpm, and a short ablation process of less than 30 s. In addition, the burr should be advanced gradually with a pecking motion and avoid deceleration beyond 5000 rpm. Furthermore, performing RA through the radial access results in lower bleeding and fewer vascular complications, while achieving similar procedural success compared to the femoral access [37].

The occurrence of MACEs after RA is affected by factors such as stent type, lesion complexity, and patient-specific factors. In patients undergoing RA, drug-eluting stents (DESs) have shown better outcomes than bare-metal stents (BMSs), mainly due to a reduced need for repeat revascularization—a result that aligns with findings from larger clinical trials and registry studies [22]. In addition, RA followed by stent implantation has shown favorable in-hospital and follow-up outcomes, with DESs associated with a reduced incidence of MACEs [40]. The implementation of DESs has significantly lowered the rates of restenosis, TLR, and MACEs across a range of lesion types [14].

Furthermore, the implantation of second-generation drug-eluting stents (DESs), in conjunction with modern RA techniques, has led to improvements in procedural success rates, reduction in late lumen loss (LLL), decreased incidence of restenosis, lower frequency of MACEs, and significant decline in other complications for patients with calcified lesions. These favorable results are largely due to improved procedural techniques and the use of advanced modern devices [41]. On the other hand, routine angiographic follow-up at 9 months has not demonstrated a significant decrease in MACEs and has shown increased late lumen loss (LLL) in lesions treated with RA [22]. Among RA-facilitated PCI strategies, combining RA with drug-eluting stents (DESs) has produced the best outcomes, leveraging both the structural support of the stent and its antiproliferative effects to minimize restenosis and lower MACE rates [42]. Overall, while RA remains a valuable tool for managing calcified lesions, careful patient selection and technique optimization are crucial for improving long-term outcomes.

Percutaneous coronary interventions increase intracoronary concentrations of inflammatory markers like TNF-α and IL-6 [43]. RA can also reduce serum inflammatory factors and plaque stabilization factors [44], potentially influencing the presentation and outcomes of coronary syndromes [45]. Rotatory atherectomy and balloon angioplasty both increase intracoronary IL-6 concentrations post-procedure [43].

### Pathomechanism of Adverse Outcomes After Rotational Atherectomy

**Figure 6:**
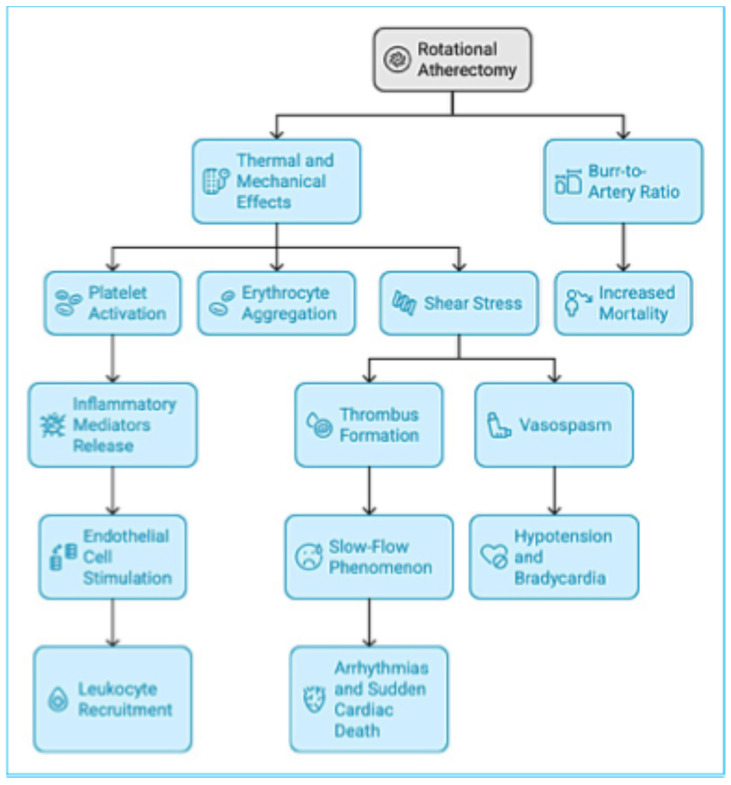
Pathomechanism of adverse outcomes after rotational atherectomy.

RA exerts significant thermal and mechanical effects on coronary arteries, influencing vascular injury, inflammation, and clinical outcomes (Figure 6). The interaction between the high-speed rotating burr and calcified plaque generates friction, leading to localized heat production and subsequent thermal injury. This thermal damage has been closely linked to platelet activation, erythrocyte aggregation, and smooth muscle proliferation, all of which contribute to coronary restenosis [46,47]. Activated platelets release a variety of inflammatory mediators, including growth factors, cytokines, and chemokines, which stimulate endothelial cells and facilitate the recruitment of leukocytes to the site of injury [48]. In patients with coronary artery disease, platelet activation is linked to elevated levels of inflammatory cytokines such as IL-1β, IL-6, and TNF-α [49].

The shear stress generated by the rotating burr is a key factor in triggering platelet activation and thrombus formation. The mechanical force applied to the arterial wall may lead to direct tissue injury and red blood cell rupture, which further promotes platelet aggregation [50]. This process occurs in two stages: first, von Willebrand factor (vWF) binds to the GPIb receptor on the platelet surface, initiating early platelet adhesion. In the subsequent phase, the release of endogenous adenosine diphosphate (ADP) perpetuates platelet activation by promoting continuous interactions between vWF, fibrinogen, and the GPIIb/IIIa receptor [51,52]. This cascade results in the formation of large platelet aggregates, triggering an irreversible clotting response [50]. Shear stress also affects endothelial function by stimulating the release of pro-inflammatory cytokines such as IL-6 and TNF-α, which further drive the inflammatory response. Notably, IL-6 plays a key role in regulating the liver’s production of C-reactive protein (CRP), highlighting the connection between vascular injury, systemic inflammation, and atherothrombotic processes [43].

The clinical consequences of RA are strongly influenced by procedural factors, particularly the rotational speed of the burr and the burr-to-artery ratio (BtAR). Higher rotational speeds, exceeding 170,000 rpm, have been associated with an increased risk of slow flow phenomenon, a complication characterized by impaired myocardial perfusion and compromised diastolic function. This phenomenon has been linked to life-threatening arrhythmias and, in severe cases, sudden cardiac death [53,54]. However, Sakakura et al. have suggested that reducing rotational speed does not necessarily mitigate the risk of slow flow, highlighting the complexity of optimizing procedural parameters.

Conversely, operating at lower speeds—typically below 150,000 rpm—introduces its own set of complications, most notably vasospasm. The prolonged contact between the burr and calcified plaque per unit time in the lower speed group is more likely to induce vasospasm, which can result in transient episodes of hypotension and bradycardia [55].

The burr-to-artery ratio has emerged as a critical determinant of clinical outcomes. A higher BtAR has been linked to increased mortality, potentially due to excessive debris production, heightened platelet activation, and microvascular embolization. These factors collectively contribute to myocardial systolic dysfunction [56]. RA utilizes a high-speed rotating burr to modify calcified plaques, which can lead to vessel injury. The resulting damage to the endothelium may contribute to microvascular obstruction and distal embolization, elevating the risk of myocardial infarction and other cardiac events [43,56].

This meta-analysis is limited by potential sources of bias inherent in aggregate data analyses. The inclusion of a wide range of PCI and atherectomy procedures, combined with the analysis of diverse outcomes, introduced substantial heterogeneity. Notably, several outcomes in our analysis demonstrated substantial heterogeneity, including stroke (I^2^ = 88%), fluoroscopy time (I^2^ = 84%), IL-6 (I^2^ = 98%), and contrast volume (I^2^ = 89%). These variations likely reflect significant differences in study design, population characteristics, procedural protocols, and outcome measurement across the included studies. The heterogeneity observed in stroke outcomes may be attributed to variations in baseline cerebrovascular risk and differences in follow-up duration. High variability in fluoroscopy time may be influenced by operator experience, lesion complexity, and the use of intravascular imaging. Inflammatory markers, such as IL-6, showed extreme heterogeneity due to the limited number of studies. Similarly, contrast volume discrepancies may relate to procedural technique, vessel size, or imaging guidance strategies.

Collectively, variations across studies may have influenced outcome reporting and contributed to variability in the pooled estimates. Although the funnel plot appeared symmetric, the possibility of publication bias cannot be excluded, particularly given the likelihood of underreporting non-significant or unfavorable results. To mitigate these limitations, standardized data extraction, rigorous quality appraisal, and the use of random-effects models were employed. Nevertheless, these factors must be considered when interpreting the generalizability of the findings.

## 5. Conclusions

While RA remains a valuable plaque modification strategy for the treatment of severely calcified coronary lesions, it is associated with increased long-term risks—including MACEs, all-cause mortality, TLR, bleeding, and prolonged fluoroscopy time—compared to other invasive approaches. These findings underscore the importance of careful patient selection and thorough evaluation of long-term outcomes when considering RA in clinical practice. To improve the safety and effectiveness of RA, future research should focus on optimizing procedural parameters, assessing long-term inflammatory and neurovascular outcomes, identifying patient-specific risk predictors, and generating real-world and cost-effectiveness data to support evidence-based decision-making. This study is limited by clinical and methodological heterogeneity among included trials, as well as small sample sizes for certain outcomes, which may affect the generalizability of the results.

## Figures and Tables

**Table 1:** PICOs of systematic review.

**Population**	Patients undergoing PCI for (moderate to severe) calcified coronary artery disease
**Intervention**	Rotational atherectomy (RA)
**Comparison**	Standard/conventional PCI, Percutaneous Transluminal Coronary Angioplasty (PTCA), or other atherectomy techniques (i.e., orbital atherectomy (OA), Excimer Laser Coronary Angioplasty (ELCA), intracoronary lithotripsy (IVL), or Transmyocardial Laser Revascularization (TMLR)
**Outcomes**	Inflammatory markers (i.e., CRP, IL-6, other markers)
	Clinical outcomes: in-stent restenosis, procedural complications (i.e., coronary artery dissection, device-induced coronary perforation, cardiac tamponade), slow flow/no reflow, myocardial infarction (MI), stroke, emergency coronary bypass graft (CABG), target vessel revascularization (TVR), target lesion revascularization (TLR), mortality, and composite MACEs

**Table 2:** Risk of bias for observational studies (*n* = 88) using the Newcastle–Ottawa Score (NOS).

Study, Year	Selection				Comparability		Outcome			Overall	Risk of Bias
	Representative of the Exposed Cohort	Selection of External Control	Ascertainment of Exposure	Outcome Not Present at Start	Main Factor	Additional Factor	Assessment of Outcomes	Sufficient Follow-Up Time	Adequacy for Follow-Up		
Barret, 2020	*	*	*	*	*	*	*	*	*	9	Low
Blachutzik, 2023	*		*	*	*		*	*	*	7	Low
Chambers, 2019		*	*	*	*	*	*	*	*	8	Low
Clavijo, 2006	*		*	*	*		*	*	*	7	Low
Dahdouh, 2013			*	*	*		*	*	*	6	Moderate
Dong, 2023	*		*	*	*		*	*	*	7	Low
El Hajj, 2020			*	*	*		*	*	*	7	Low
Farhat, 2023	*	*	*	*	*	*		*	*	8	Low
Fujimoto, 2010		*	*	*	*	*	*	*		7	Low
Gallinoro, 2022	*		*	*	*		*		*	6	Moderate
Gioia, 2000	*			*			*		*	4	Moderate
Gorol, 2018	*		*	*	*	*	*	*	*	8	Low
Hemetsberger, 2024	*		*		*		*		*	5	Moderate
Hernandez, 2018				*			*		*	3	High
Hoffmann, 1998	*	*	*	*	*	*	*	*	*	9	Low
Ielasi, 2017	*	*	*	*			*	*	*	7	Low
Iwasaki, 2020	*		*	*	*		*	*	*	7	Low
Januszek, 2017	*	*	*	*	*	*	*		*	8	Low
Koifman, 2018	*	*	*	*	*	*	*		*	8	Low
Lee, 2017	*		*	*	*		*		*	6	Moderate
Li, 2019			*	*	*		*		*	5	Moderate
Maier, 2024	*	*	*	*	*	*	*		*	9	Low
Meraj, 2018	*	*		*	*	*	*		*	7	Low
Motwani, 2000	*		*	*			*		*	5	Moderate
Mousa, 2023	*	*	*	*	*	*	*	*	*	9	Low
Okamoto, 2019	*	*	*	*	*	*	*	*	*	9	Low
Rola, 2022	*	*	*	*	*		*	*	*	8	Low
Sareen, 2017	*	*	*	*	*	*	*	*	*	9	Low
Tamekiyo, 2009	*		*	*	*		*	*		7	Low
Tang, 2016	*	*	*	*	*	*	*	*	*	9	Low
Tian, 2015	*	*	*	*			*	*	*	7	Low
Vaquerizo, 2010	*		*	*	*	*	*	*	*	8	Low
Wong, 2023	*		*	*			*			4	Moderate
Al Maclsaac, 1995	*		*	*	*		*	*	*	7	Low
Ayoub, 2023	*		*	*	*		*	*	*	7	Low
L. Desta, 2022	*		*	*	*		*	*	*	7	Low
Mezilis, 2010	*		*	*			*	*	*	6	Moderate
Jiang, 2012	*		*	*			*	*		5	Moderate
Jung 2023	*	*	*	*	*	*	*	*	*	9	Low
Kato, 2012		*	*	*	*	*	*	*	*	8	Low
Kawamoto, 2016	*		*	*	*	*	*	*	*	8	Low
Khattab, 2007	*	*	*	*	*	*	*	*		8	Low
Kotronias, 2019		*	*	*	*	*	*	*	*	8	Low
Kubota, 2010	*	*	*	*	*		*	*		7	Low
Meuwissen, 2003	*	*	*	*	*		*		*	7	Low
Wu, 2023	*		*	*	*	*	*		*	7	Low
Abdel-wahab, 2012	*	*	*	*	*	*	*	*	*	9	Low
Benezet, 2011			*	*	*		*	*	*	6	Moderate
Bouisset, 2021	*		*	*	*	*	*	*	*	8	Low
Chen, 2016	*		*	*	*		*	*	*	7	Low
Chiang, 2013	*		*	*	*	*	*	*	*	8	Low
Chiou, 2020	*		*	*			*	*	*	6	Moderate
Cho, 2000			*	*	*	*	*	*		6	Moderate
Dardas, 2011	*		*	*	*		*	*	*	8	Low
de Melo, 2015	*		*	*	*		*	*	*	7	Low
Dhillon, 2019			*	*	*		*	*	*	6	Moderate
Dong, 2021			*	*	*		*	*	*	6	Moderate
Dong, 2020	*		*	*	*	*	*	*	*	8	Low
Eftychiou, 2016	*		*	*	*		*	*	*	8	Low
Ferri, 2016			*	*	*	*	*	*	*	7	Low
Furuichi, 2009	*		*	*	*		*	*	*	7	Low
Garcia-Lara, 2011		*	*	*	*	*	*	*	*	8	Low
Jujo K, 2019	*	*	*	*	*	*	*	*	*	9	Low
Kauffman, 1989	*		*	*			*			4	Moderate
Kawamoto, 2016	*		*	*	*		*	*	*	8	Low
Koch, 2002			*	*	*		*		*	5	Moderate
Lippmann, 2017			*	*	*	*	*		*	6	Moderate
Lunardi, 2020			*	*	*	*	*	*	*	7	Low
Malik, 2021	*		*	*	*	*	*	*	*	8	Low
Naito, 2012	*	*	*	*	*	*	*	*		8	Low
Mankerious, 2020	*		*	*	*	*	*	*	*	8	Low
Naito, 2012	*	*	*	*	*	*	*	*		8	Low
Patel, 1997	*		*	*			*	*		5	Moderate
Popma, 1993			*		*	*	*	*	*	6	Moderate
Rathore, 2010	*	*	*	*	*	*	*		*	8	Low
Rissanen, 2017	*		*	*			*	*	*	6	Moderate
Sakakura, 2016	*	*	*	*	*	*	*		*	8	Low
Sharma, 1998	*		*	*			*	*	*	6	Moderate
Simsek, 2022	*	*	*	*	*	*	*		*	8	Low
Takagi, 2022	*		*	*	*	*	*	*	*	8	Low
Tan, 2021	*		*	*			*	*	*	6	Moderate
Tern, 2024	*		*	*			*		*	5	Moderate
Tervo, 2022	*		*	*	*	*	*	*	*	8	Low
Towashiraporn, 2023	*		*	*			*	*	*	6	Moderate
Towashiraporn, 2022	*		*	*	*	*	*	*	*	8	Low
Watanabe, 2018	*		*	*	*	*	*		*	7	Low
Wei, 2016	*		*	*	*	*	*	*	*	8	Low
Whiteside, 2018	*		*	*			*		*	5	Moderate
Whiteside, 2019			*	*	*	*	*	*	*	7	Low
Yabushita, 2014	*		*	*			*	*	*	6	Moderate
Yoneda, 2023	*	*	*	*	*	*	*	*	*	9	Low
Zimarino,1994	*		*	*			*	*	*	6	Moderate

**Table 3:** Effect of rotatory atherectomy (RA) on clinical and inflammatory outcomes as reported by the recent literature.

No	Outcomes of Interest	Studies (*n*)	Participants (*n*)
1	Composite MACEs	24	360,627
2	Mortality	28	327,228
3	Myocardial infarction (MI)	22	327,526
4	Total vascular revascularization (TVR)	9	4287
5	Total lesion revascularization (TLR)	16	8056
6	Slow/no flow (TIMI flow < 3)	9	318,417
7	Coronary dissection	8	277,489
8	Coronary perforation	5	3004
9	Cardiac tamponade or effusion	5	34,580
10	Stent thrombosis	8	3981
11	In-stent restenosis	5	1428
12	Heart failure (HF) NYHA IV	3	1235
13	Stroke	6	309,491
14	Bleeding	3	350,351
15	Emergency CABG	3	717
16	Fluoroscopy time	4	1841
17	Contrast volume	5	2281
18	Interleukin-6 (IL-6)	2	108

## Data Availability

All data generated or analyzed during this study are included in this published article, available from the corresponding author (myaqanitha@gmail.com or a.qanitha@unhas.ac.id) upon reasonable request.

## Supplementary Materials

The following supporting information can be downloaded at https://www.mdpi.com/article/10.3390/jcm14155389/s1, Table S1: Searching strategy on PubMed and EMBASE; Table S2: PRISMA checklist; Table S3: Included studies in systematic review (*n* = 110) and meta-analysis (*n* = 36).

## Abbreviations

RA	Rotational atherectomy
OA	Orbital atherectomy
IL-6	Interleukin-6
CRP	C-reactive protein
TLR	Total lesion revascularization
TVR	Total vascular revascularization
MACE	Major adverse cardiovascular event
PCI	Percutaneous coronary intervention
CABG	Coronary bypass graft
PTCA	Percutaneous Transluminal Coronary Angioplasty
ELCA	Excimer Laser Coronary Angioplasty
IVL	Intracoronary lithotripsy
TMLR	Transmyocardial Laser Revascularization
CAD	Coronary artery disease
DES	Drug-eluting stent
BMS	Bare-metal stent
LLL	Late lumen loss
vWF	von Willebrand factors
