# Supplementary material for: Clinical and Inflammatory Outcomes of Rotational Atherectomy in Calcified Coronary Lesions: A Systematic Review and Meta-Analysis
Source: J Clin Med. 2025 Jul 31;14(15):5389. doi: 10.3390/jcm14155389 (PMC12347861; doi:10.3390/jcm14155389)
Supplement: Supplementary file 1 [file jcm-14-05389-s001.zip › jcm-3688480_Supplementary Materials (Table S1).pdf]

Table S1(a). Searching strategy on PubMed

Search number	Query	Search Details on PubMed	Results
11	#10 AND English [la]	(((("atherectomy"[MeSH Terms] OR "atherectomy"[All Fields] OR "atherectomies"[All Fields] OR ("atherectomy, coronary"[MeSH Terms] OR ("atherectomy"[All Fields] AND "coronary"[All Fields]) OR "coronary atherectomy"[All Fields] OR ("rotational"[All Fields] AND "atherectomy"[All Fields]) OR "rotational atherectomy"[All Fields])) AND ("coronary artery disease"[MeSH Terms] OR ("coronary"[All Fields] AND "artery"[All Fields] AND "disease"[All Fields]) OR "coronary artery disease"[All Fields] OR (("calcifiability"[All Fields] OR "calcifiable"[All Fields] OR "calcified"[All Fields] OR "calcifier"[All Fields] OR "calcifiers"[All Fields] OR "calcifies"[All Fields] OR "calcify"[All Fields] OR "calcifying"[All Fields]) AND ("coronary artery disease"[MeSH Terms] OR ("coronary"[All Fields] AND "artery"[All Fields] AND "disease"[All Fields]) OR "coronary artery disease"[All Fields] OR ("constriction, pathologic"[MeSH Terms] OR ("constriction"[All Fields] AND "pathologic"[All Fields]) OR "pathologic constriction"[All Fields] OR "stenosi"[All Fields] OR "stenosis"[All Fields]) OR ("thrombosis"[MeSH Terms] OR "thrombosis"[All Fields] OR "thrombus"[All Fields]))) OR ((("stable"[All Fields] OR "stabled"[All Fields] OR "stables"[All Fields] OR "stabling"[All Fields]) AND ("coronary artery disease"[MeSH Terms] OR ("coronary"[All Fields] AND "artery"[All Fields] AND "disease"[All Fields]) OR "coronary artery disease"[All Fields] OR ("angina pectoris"[MeSH Terms] OR ("angina"[All Fields] AND "pectoris"[All Fields]) OR "angina pectoris"[All Fields] OR "angina"[All Fields] OR "anginas"[All Fields]) OR ("angina pectoris"[MeSH Terms] OR ("angina"[All Fields] AND "pectoris"[All Fields]) OR "angina pectoris"[All Fields]))) OR "SCAD"[All Fields]) OR ("atherosclerosis"[MeSH Terms] OR "atherosclerosis"[All Fields] OR ("atherosclerotic"[All Fields] AND "cardiovascular"[All Fields] AND "disease"[All Fields]) OR "atherosclerotic cardiovascular disease"[All Fields] OR ("ascvd"[All Fields] OR "ascvds"[All Fields])) OR ((("chronic"[All Fields] OR "chronical"[All Fields] OR "chronically"[All Fields] OR "chronicities"[All Fields] OR "chronicity"[All Fields] OR "chronicization"[All Fields] OR "chronics"[All Fields]) AND ("coronaries"[All Fields] OR "heart"[MeSH Terms] OR "heart"[All Fields] OR "coronary"[All Fields]) AND ("syndrom"[All Fields] OR "syndromal"[All Fields] OR "syndromally"[All Fields] OR "syndrome"[MeSH Terms] OR "syndrome"[All Fields] OR "syndromes"[All Fields] OR "syndrome s"[All Fields] OR "syndromic"[All Fields] OR "syndroms"[All Fields])) OR ("cell commun signal"[Journal] OR "ccs"[All Fields]))) AND ("inflammation"[MeSH Terms] OR "inflammation"[All Fields] OR "inflammations"[All Fields] OR "inflammation s"[All Fields] OR (("inflammatories"[All Fields] OR "inflammatory"[All Fields]) AND "marker*"[All Fields]) OR ("curr res psychol"[Journal] OR "crp"[All Fields]) OR ("c reactive protein"[MeSH	800

		Terms] OR ("c reactive"[All Fields] AND "protein"[All Fields]) OR "c reactive protein"[All Fields] OR "c reactive protein"[All Fields]) OR ("interleukin 6"[MeSH Terms] OR "interleukin 6"[All Fields] OR "interleukin 6"[All Fields]) OR ("interleukin 6"[MeSH Terms] OR "interleukin 6"[All Fields] OR "il 6"[All Fields]) OR ("tumour necrosis factor alpha"[All Fields] OR "tumor necrosis factor alpha"[MeSH Terms] OR ("tumor"[All Fields] AND "necrosis"[All Fields] AND "factor alpha"[All Fields]) OR "tumor necrosis factor alpha"[All Fields] OR ("tumor"[All Fields] AND "necrosis"[All Fields] AND "factor"[All Fields] AND "alpha"[All Fields]) OR "tumor necrosis factor alpha"[All Fields]) OR "TNF-A"[All Fields] OR ("in-stent"[All Fields] AND "restenosis"[All Fields]) OR ("constriction, pathologic"[MeSH Terms] OR ("constriction"[All Fields] AND "pathologic"[All Fields]) OR "pathologic constriction"[All Fields] OR "stenosi"[All Fields] OR "stenosis"[All Fields]) OR (("acute"[All Fields] OR "acutely"[All Fields] OR "acutes"[All Fields]) AND ("thrombose"[All Fields] OR "thrombosing"[All Fields] OR "thrombosis"[MeSH Terms] OR "thrombosis"[All Fields] OR "thrombosed"[All Fields] OR "thromboses"[All Fields])) OR (("systemic"[All Fields] OR "systemically"[All Fields] OR "systemics"[All Fields]) AND ("inflammation"[MeSH Terms] OR "inflammation"[All Fields] OR "inflammations"[All Fields] OR "inflammation s"[All Fields])) OR ("post-procedure"[All Fields] AND "complication*"[All Fields])) AND ("PCI"[All Fields] OR ("percutaneous coronary intervention"[MeSH Terms] OR ("percutaneous"[All Fields] AND "coronary"[All Fields] AND "intervention"[All Fields]) OR "percutaneous coronary intervention"[All Fields]) OR ("angioplasty, balloon, coronary"[MeSH Terms] OR ("angioplasty"[All Fields] AND "balloon"[All Fields] AND "coronary"[All Fields]) OR "coronary balloon angioplasty"[All Fields] OR ("percutaneous"[All Fields] AND "transluminal"[All Fields] AND "coronary"[All Fields] AND "angioplasty"[All Fields]) OR "percutaneous transluminal coronary angioplasty"[All Fields]) OR ("angioplasty, balloon, coronary"[MeSH Terms] OR ("angioplasty"[All Fields] AND "balloon"[All Fields] AND "coronary"[All Fields]) OR "coronary balloon angioplasty"[All Fields] OR "ptca"[All Fields]) OR ("balloon"[All Fields] OR "balloon s"[All Fields] OR "balloons"[All Fields]))) NOT ("editorial"[Publication Type] OR "editorial"[All Fields] OR ("letter"[Publication Type] OR "correspondence as topic"[MeSH Terms] OR "letter"[All Fields]) OR "comment*"[All Fields])) NOT ("animals"[MeSH Terms:noexp] OR "animal"[All Fields])) AND "English"[Language]	
10	#9 NOT Animal	((("atherectomy"[MeSH Terms] OR "atherectomy"[All Fields] OR "atherectomies"[All Fields] OR ("atherectomy, coronary"[MeSH Terms] OR ("atherectomy"[All Fields] AND "coronary"[All Fields]) OR "coronary atherectomy"[All Fields] OR ("rotational"[All Fields] AND "atherectomy"[All Fields]) OR "rotational atherectomy"[All Fields])) AND ("coronary artery disease"[MeSH Terms] OR ("coronary"[All Fields] AND "artery"[All Fields] AND "disease"[All Fields]) OR "coronary artery	888

		disease"[All Fields] OR (("calcifiability"[All Fields] OR "calcifiable"[All Fields] OR "calcified"[All Fields] OR "calcifier"[All Fields] OR "calcifiers"[All Fields] OR "calcifies"[All Fields] OR "calcify"[All Fields] OR "calcifying"[All Fields]) AND ("coronary artery disease"[MeSH Terms] OR ("coronary"[All Fields] AND "artery"[All Fields] AND "disease"[All Fields]) OR "coronary artery disease"[All Fields] OR ("constriction, pathologic"[MeSH Terms] OR ("constriction"[All Fields] AND "pathologic"[All Fields]) OR "pathologic constriction"[All Fields] OR "stenosi"[All Fields] OR "stenosis"[All Fields]) OR ("thrombosis"[MeSH Terms] OR "thrombosis"[All Fields] OR "thrombus"[All Fields]))) OR ((("stable"[All Fields] OR "stabled"[All Fields] OR "stables"[All Fields] OR "stabling"[All Fields]) AND ("coronary artery disease"[MeSH Terms] OR ("coronary"[All Fields] AND "artery"[All Fields] AND "disease"[All Fields]) OR "coronary artery disease"[All Fields] OR ("angina pectoris"[MeSH Terms] OR ("angina"[All Fields] AND "pectoris"[All Fields]) OR "angina pectoris"[All Fields] OR "angina"[All Fields] OR "anginas"[All Fields]) OR ("angina pectoris"[MeSH Terms] OR ("angina"[All Fields] AND "pectoris"[All Fields]) OR "angina pectoris"[All Fields]))) OR "SCAD"[All Fields]) OR ("atherosclerosis"[MeSH Terms] OR "atherosclerosis"[All Fields] OR ("atherosclerotic"[All Fields] AND "cardiovascular"[All Fields] AND "disease"[All Fields]) OR "atherosclerotic cardiovascular disease"[All Fields] OR ("ascvd"[All Fields] OR "ascvds"[All Fields])) OR ((("chronic"[All Fields] OR "chronical"[All Fields] OR "chronically"[All Fields] OR "chronicities"[All Fields] OR "chronicity"[All Fields] OR "chronicization"[All Fields] OR "chronics"[All Fields]) AND ("coronaries"[All Fields] OR "heart"[MeSH Terms] OR "heart"[All Fields] OR "coronary"[All Fields]) AND ("syndrom"[All Fields] OR "syndromal"[All Fields] OR "syndromally"[All Fields] OR "syndrome"[MeSH Terms] OR "syndrome"[All Fields] OR "syndromes"[All Fields] OR "syndrome s"[All Fields] OR "syndromic"[All Fields] OR "syndroms"[All Fields])) OR ("cell commun signal"[Journal] OR "ccs"[All Fields]))) AND ("inflammation"[MeSH Terms] OR "inflammation"[All Fields] OR "inflammations"[All Fields] OR "inflammation s"[All Fields] OR (("inflammatories"[All Fields] OR "inflammatory"[All Fields]) AND "marker*"[All Fields]) OR ("curr res psychol"[Journal] OR "crp"[All Fields]) OR ("c reactive protein"[MeSH Terms] OR ("c reactive"[All Fields] AND "protein"[All Fields]) OR "c reactive protein"[All Fields] OR "c reactive protein"[All Fields]) OR ("interleukin 6"[MeSH Terms] OR "interleukin 6"[All Fields] OR "interleukin 6"[All Fields]) OR ("interleukin 6"[MeSH Terms] OR "interleukin 6"[All Fields] OR "il 6"[All Fields]) OR ("tumour necrosis factor alpha"[All Fields] OR "tumor necrosis factor alpha"[MeSH Terms] OR ("tumor"[All Fields] AND "necrosis"[All Fields] AND "factor alpha"[All Fields]) OR "tumor necrosis factor alpha"[All Fields] OR ("tumor"[All Fields] AND "necrosis"[All Fields] AND "factor"[All Fields] AND "alpha"[All Fields]) OR "tumor necrosis factor alpha"[All Fields]) OR "TNF-A"[All Fields] OR ("in-stent"[All Fields] AND "restenosis"[All Fields]) OR ("constriction, pathologic"[MeSH Terms] OR ("constriction"[All Fields]	

		AND "pathologic"[All Fields]) OR "pathologic constriction"[All Fields] OR "stenosi"[All Fields] OR "stenosis"[All Fields]) OR (("acute"[All Fields] OR "acutely"[All Fields] OR "acutes"[All Fields]) AND ("thrombose"[All Fields] OR "thrombosing"[All Fields] OR "thrombosis"[MeSH Terms] OR "thrombosis"[All Fields] OR "thrombosed"[All Fields] OR "thromboses"[All Fields])) OR (("systemic"[All Fields] OR "systemically"[All Fields] OR "systemics"[All Fields]) AND ("inflammation"[MeSH Terms] OR "inflammation"[All Fields] OR "inflammations"[All Fields] OR "inflammation s"[All Fields])) OR ("post-procedure"[All Fields] AND "complication*"[All Fields])) AND ("PCI"[All Fields] OR ("percutaneous coronary intervention"[MeSH Terms] OR ("percutaneous"[All Fields] AND "coronary"[All Fields] AND "intervention"[All Fields]) OR "percutaneous coronary intervention"[All Fields]) OR ("angioplasty, balloon, coronary"[MeSH Terms] OR ("angioplasty"[All Fields] AND "balloon"[All Fields] AND "coronary"[All Fields]) OR "coronary balloon angioplasty"[All Fields] OR ("percutaneous"[All Fields] AND "transluminal"[All Fields] AND "coronary"[All Fields] AND "angioplasty"[All Fields]) OR "percutaneous transluminal coronary angioplasty"[All Fields]) OR ("angioplasty, balloon, coronary"[MeSH Terms] OR ("angioplasty"[All Fields] AND "balloon"[All Fields] AND "coronary"[All Fields]) OR "coronary balloon angioplasty"[All Fields] OR "ptca"[All Fields]) OR ("balloon"[All Fields] OR "balloon s"[All Fields] OR "balloons"[All Fields]))) NOT ("editorial"[Publication Type] OR "editorial"[All Fields] OR ("letter"[Publication Type] OR "correspondence as topic"[MeSH Terms] OR "letter"[All Fields]) OR "comment*"[All Fields])) NOT ("animals"[MeSH Terms:noexp] OR "animal"[All Fields])	
9	#8 NOT (editorial OR letter OR comment*)	(("atherectomy"[MeSH Terms] OR "atherectomy"[All Fields] OR "atherectomies"[All Fields] OR ("atherectomy, coronary"[MeSH Terms] OR ("atherectomy"[All Fields] AND "coronary"[All Fields]) OR "coronary atherectomy"[All Fields] OR ("rotational"[All Fields] AND "atherectomy"[All Fields]) OR "rotational atherectomy"[All Fields])) AND ("coronary artery disease"[MeSH Terms] OR ("coronary"[All Fields] AND "artery"[All Fields] AND "disease"[All Fields]) OR "coronary artery disease"[All Fields] OR (("calcifiability"[All Fields] OR "calcifiable"[All Fields] OR "calcified"[All Fields] OR "calcifier"[All Fields] OR "calcifiers"[All Fields] OR "calcifies"[All Fields] OR "calcify"[All Fields] OR "calcifying"[All Fields]) AND ("coronary artery disease"[MeSH Terms] OR ("coronary"[All Fields] AND "artery"[All Fields] AND "disease"[All Fields]) OR "coronary artery disease"[All Fields] OR ("constriction, pathologic"[MeSH Terms] OR ("constriction"[All Fields] AND "pathologic"[All Fields]) OR "pathologic constriction"[All Fields] OR "stenosi"[All Fields] OR "stenosis"[All Fields]) OR ("thrombosis"[MeSH Terms] OR "thrombosis"[All Fields] OR "thrombus"[All Fields]))) OR ((("stable"[All Fields] OR "stabled"[All Fields] OR "stables"[All Fields] OR "stabling"[All Fields]) AND ("coronary artery disease"[MeSH Terms] OR ("coronary"[All Fields] AND "artery"[All Fields] AND "disease"[All Fields])	914

		OR "coronary artery disease"[All Fields] OR ("angina pectoris"[MeSH Terms] OR ("angina"[All Fields] AND "pectoris"[All Fields]) OR "angina pectoris"[All Fields] OR "angina"[All Fields] OR "anginas"[All Fields]) OR ("angina pectoris"[MeSH Terms] OR ("angina"[All Fields] AND "pectoris"[All Fields]) OR "angina pectoris"[All Fields]))) OR "SCAD"[All Fields]) OR ("atherosclerosis"[MeSH Terms] OR "atherosclerosis"[All Fields] OR ("atherosclerotic"[All Fields] AND "cardiovascular"[All Fields] AND "disease"[All Fields]) OR "atherosclerotic cardiovascular disease"[All Fields] OR ("ascvd"[All Fields] OR "ascvds"[All Fields])) OR ((("chronic"[All Fields] OR "chronical"[All Fields] OR "chronically"[All Fields] OR "chronicities"[All Fields] OR "chronicity"[All Fields] OR "chronicization"[All Fields] OR "chronics"[All Fields]) AND ("coronaries"[All Fields] OR "heart"[MeSH Terms] OR "heart"[All Fields] OR "coronary"[All Fields]) AND ("syndrom"[All Fields] OR "syndromal"[All Fields] OR "syndromally"[All Fields] OR "syndrome"[MeSH Terms] OR "syndrome"[All Fields] OR "syndromes"[All Fields] OR "syndrome s"[All Fields] OR "syndromic"[All Fields] OR "syndroms"[All Fields])) OR ("cell commun signal"[Journal] OR "ccs"[All Fields]))) AND ("inflammation"[MeSH Terms] OR "inflammation"[All Fields] OR "inflammations"[All Fields] OR "inflammation s"[All Fields] OR (("inflammatories"[All Fields] OR "inflammatory"[All Fields]) AND "marker*"[All Fields]) OR ("curr res psychol"[Journal] OR "crp"[All Fields]) OR ("c reactive protein"[MeSH Terms] OR ("c reactive"[All Fields] AND "protein"[All Fields]) OR "c reactive protein"[All Fields] OR "c reactive protein"[All Fields]) OR ("interleukin 6"[MeSH Terms] OR "interleukin 6"[All Fields] OR "interleukin 6"[All Fields]) OR ("interleukin 6"[MeSH Terms] OR "interleukin 6"[All Fields] OR "il 6"[All Fields]) OR ("tumour necrosis factor alpha"[All Fields] OR "tumor necrosis factor alpha"[MeSH Terms] OR ("tumor"[All Fields] AND "necrosis"[All Fields] AND "factor alpha"[All Fields]) OR "tumor necrosis factor alpha"[All Fields] OR ("tumor"[All Fields] AND "necrosis"[All Fields] AND "factor"[All Fields] AND "alpha"[All Fields]) OR "tumor necrosis factor alpha"[All Fields]) OR "TNF-A"[All Fields] OR ("in-stent"[All Fields] AND "restenosis"[All Fields]) OR ("constriction, pathologic"[MeSH Terms] OR ("constriction"[All Fields] AND "pathologic"[All Fields]) OR "pathologic constriction"[All Fields] OR "stenosi"[All Fields] OR "stenosis"[All Fields]) OR (("acute"[All Fields] OR "acutely"[All Fields] OR "acutes"[All Fields]) AND ("thrombose"[All Fields] OR "thrombosing"[All Fields] OR "thrombosis"[MeSH Terms] OR "thrombosis"[All Fields] OR "thrombosed"[All Fields] OR "thromboses"[All Fields])) OR (("systemic"[All Fields] OR "systemically"[All Fields] OR "systemics"[All Fields]) AND ("inflammation"[MeSH Terms] OR "inflammation"[All Fields] OR "inflammations"[All Fields] OR "inflammation s"[All Fields])) OR ("post-procedure"[All Fields] AND "complication*"[All Fields]))) AND ("PCI"[All Fields] OR ("percutaneous coronary intervention"[MeSH Terms] OR ("percutaneous"[All Fields] AND "coronary"[All Fields] AND "intervention"[All Fields]) OR "percutaneous coronary intervention"[All	

		Fields]) OR ("angioplasty, balloon, coronary"[MeSH Terms] OR ("angioplasty"[All Fields] AND "balloon"[All Fields] AND "coronary"[All Fields]) OR "coronary balloon angioplasty"[All Fields] OR ("percutaneous"[All Fields] AND "transluminal"[All Fields] AND "coronary"[All Fields] AND "angioplasty"[All Fields]) OR "percutaneous transluminal coronary angioplasty"[All Fields]) OR ("angioplasty, balloon, coronary"[MeSH Terms] OR ("angioplasty"[All Fields] AND "balloon"[All Fields] AND "coronary"[All Fields]) OR "coronary balloon angioplasty"[All Fields] OR "ptca"[All Fields]) OR ("balloon"[All Fields] OR "balloon s"[All Fields] OR "balloons"[All Fields]))) NOT ("editorial"[Publication Type] OR "editorial"[All Fields] OR ("letter"[Publication Type] OR "correspondence as topic"[MeSH Terms] OR "letter"[All Fields]) OR "comment*"[All Fields])	
8	#1 AND #2 AND #7	("atherectomy"[MeSH Terms] OR "atherectomy"[All Fields] OR "atherectomies"[All Fields] OR ("atherectomy, coronary"[MeSH Terms] OR ("atherectomy"[All Fields] AND "coronary"[All Fields]) OR "coronary atherectomy"[All Fields] OR ("rotational"[All Fields] AND "atherectomy"[All Fields]) OR "rotational atherectomy"[All Fields])) AND ("coronary artery disease"[MeSH Terms] OR ("coronary"[All Fields] AND "artery"[All Fields] AND "disease"[All Fields]) OR "coronary artery disease"[All Fields] OR (("calcifiability"[All Fields] OR "calcifiable"[All Fields] OR "calcified"[All Fields] OR "calcifier"[All Fields] OR "calcifiers"[All Fields] OR "calcifies"[All Fields] OR "calcify"[All Fields] OR "calcifying"[All Fields]) AND ("coronary artery disease"[MeSH Terms] OR ("coronary"[All Fields] AND "artery"[All Fields] AND "disease"[All Fields]) OR "coronary artery disease"[All Fields] OR ("constriction, pathologic"[MeSH Terms] OR ("constriction"[All Fields] AND "pathologic"[All Fields]) OR "pathologic constriction"[All Fields] OR "stenosi"[All Fields] OR "stenosis"[All Fields]) OR ("thrombosis"[MeSH Terms] OR "thrombosis"[All Fields] OR "thrombus"[All Fields]))) OR ((("stable"[All Fields] OR "stabled"[All Fields] OR "stables"[All Fields] OR "stabling"[All Fields]) AND ("coronary artery disease"[MeSH Terms] OR ("coronary"[All Fields] AND "artery"[All Fields] AND "disease"[All Fields]) OR "coronary artery disease"[All Fields] OR ("angina pectoris"[MeSH Terms] OR ("angina"[All Fields] AND "pectoris"[All Fields]) OR "angina pectoris"[All Fields] OR "angina"[All Fields] OR "anginas"[All Fields]) OR ("angina pectoris"[MeSH Terms] OR ("angina"[All Fields] AND "pectoris"[All Fields]) OR "angina pectoris"[All Fields]))) OR "SCAD"[All Fields]) OR ("atherosclerosis"[MeSH Terms] OR "atherosclerosis"[All Fields] OR ("atherosclerotic"[All Fields] AND "cardiovascular"[All Fields] AND "disease"[All Fields]) OR "atherosclerotic cardiovascular disease"[All Fields] OR ("ascvd"[All Fields] OR "ascvds"[All Fields])) OR ((("chronic"[All Fields] OR "chronical"[All Fields] OR "chronically"[All Fields] OR "chronicities"[All Fields] OR "chronicity"[All Fields] OR "chronicization"[All Fields] OR "chronics"[All Fields]) AND ("coronaries"[All Fields] OR "heart"[MeSH Terms] OR "heart"[All Fields]	929

		OR "coronary"[All Fields]) AND ("syndrom"[All Fields] OR "syndromal"[All Fields] OR "syndromally"[All Fields] OR "syndrome"[MeSH Terms] OR "syndrome"[All Fields] OR "syndromes"[All Fields] OR "syndrome s"[All Fields] OR "syndromic"[All Fields] OR "syndroms"[All Fields])) OR ("cell commun signal"[Journal] OR "ccs"[All Fields]))) AND ("inflammation"[MeSH Terms] OR "inflammation"[All Fields] OR "inflammations"[All Fields] OR "inflammation s"[All Fields] OR (("inflammatories"[All Fields] OR "inflammatory"[All Fields]) AND "marker*"[All Fields]) OR ("curr res psychol"[Journal] OR "crp"[All Fields]) OR ("c reactive protein"[MeSH Terms] OR ("c reactive"[All Fields] AND "protein"[All Fields]) OR "c reactive protein"[All Fields] OR "c reactive protein"[All Fields]) OR ("interleukin 6"[MeSH Terms] OR "interleukin 6"[All Fields] OR "interleukin 6"[All Fields]) OR ("interleukin 6"[MeSH Terms] OR "interleukin 6"[All Fields] OR "il 6"[All Fields]) OR ("tumour necrosis factor alpha"[All Fields] OR "tumor necrosis factor alpha"[MeSH Terms] OR ("tumor"[All Fields] AND "necrosis"[All Fields] AND "factor alpha"[All Fields]) OR "tumor necrosis factor alpha"[All Fields] OR ("tumor"[All Fields] AND "necrosis"[All Fields] AND "factor"[All Fields] AND "alpha"[All Fields]) OR "tumor necrosis factor alpha"[All Fields]) OR "TNF-A"[All Fields] OR ("in-stent"[All Fields] AND "restenosis"[All Fields]) OR ("constriction, pathologic"[MeSH Terms] OR ("constriction"[All Fields] AND "pathologic"[All Fields]) OR "pathologic constriction"[All Fields] OR "stenosi"[All Fields] OR "stenosis"[All Fields]) OR (("acute"[All Fields] OR "acutely"[All Fields] OR "acutes"[All Fields]) AND ("thrombose"[All Fields] OR "thrombosing"[All Fields] OR "thrombosis"[MeSH Terms] OR "thrombosis"[All Fields] OR "thrombosed"[All Fields] OR "thromboses"[All Fields])) OR (("systemic"[All Fields] OR "systemically"[All Fields] OR "systemics"[All Fields]) AND ("inflammation"[MeSH Terms] OR "inflammation"[All Fields] OR "inflammations"[All Fields] OR "inflammation s"[All Fields])) OR ("post-procedure"[All Fields] AND "complication*"[All Fields])) AND ("PCI"[All Fields] OR ("percutaneous coronary intervention"[MeSH Terms] OR ("percutaneous"[All Fields] AND "coronary"[All Fields] AND "intervention"[All Fields]) OR "percutaneous coronary intervention"[All Fields]) OR ("angioplasty, balloon, coronary"[MeSH Terms] OR ("angioplasty"[All Fields] AND "balloon"[All Fields] AND "coronary"[All Fields]) OR "coronary balloon angioplasty"[All Fields] OR ("percutaneous"[All Fields] AND "transluminal"[All Fields] AND "coronary"[All Fields] AND "angioplasty"[All Fields]) OR "percutaneous transluminal coronary angioplasty"[All Fields]) OR ("angioplasty, balloon, coronary"[MeSH Terms] OR ("angioplasty"[All Fields] AND "balloon"[All Fields] AND "coronary"[All Fields]) OR "coronary balloon angioplasty"[All Fields] OR "ptca"[All Fields]) OR ("balloon"[All Fields] OR "balloon s"[All Fields] OR "balloons"[All Fields]))	

7	(PCI OR percutaneous coronary intervention OR percutaneous transluminal coronary angioplasty OR PTCA OR balloon)	"PCI"[All Fields] OR ("percutaneous coronary intervention"[MeSH Terms] OR ("percutaneous"[All Fields] AND "coronary"[All Fields] AND "intervention"[All Fields]) OR "percutaneous coronary intervention"[All Fields]) OR ("angioplasty, balloon, coronary"[MeSH Terms] OR ("angioplasty"[All Fields] AND "balloon"[All Fields] AND "coronary"[All Fields]) OR "coronary balloon angioplasty"[All Fields] OR ("percutaneous"[All Fields] AND "transluminal"[All Fields] AND "coronary"[All Fields] AND "angioplasty"[All Fields]) OR "percutaneous transluminal coronary angioplasty"[All Fields]) OR ("angioplasty, balloon, coronary"[MeSH Terms] OR ("angioplasty"[All Fields] AND "balloon"[All Fields] AND "coronary"[All Fields]) OR "coronary balloon angioplasty"[All Fields] OR "ptca"[All Fields]) OR ("balloon"[All Fields] OR "balloon s"[All Fields] OR "balloons"[All Fields])	180,659
6	#5 AND English [la]	(((("atherectomy"[MeSH Terms] OR "atherectomy"[All Fields] OR "atherectomies"[All Fields] OR ("atherectomy, coronary"[MeSH Terms] OR ("atherectomy"[All Fields] AND "coronary"[All Fields]) OR "coronary atherectomy"[All Fields] OR ("rotational"[All Fields] AND "atherectomy"[All Fields]) OR "rotational atherectomy"[All Fields])) AND ("coronary artery disease"[MeSH Terms] OR ("coronary"[All Fields] AND "artery"[All Fields] AND "disease"[All Fields]) OR "coronary artery disease"[All Fields] OR (("calcifiability"[All Fields] OR "calcifiable"[All Fields] OR "calcified"[All Fields] OR "calcifier"[All Fields] OR "calcifiers"[All Fields] OR "calcifies"[All Fields] OR "calcify"[All Fields] OR "calcifying"[All Fields]) AND ("coronary artery disease"[MeSH Terms] OR ("coronary"[All Fields] AND "artery"[All Fields] AND "disease"[All Fields]) OR "coronary artery disease"[All Fields] OR ("constriction, pathologic"[MeSH Terms] OR ("constriction"[All Fields] AND "pathologic"[All Fields]) OR "pathologic constriction"[All Fields] OR "stenosi"[All Fields] OR "stenosis"[All Fields]) OR ("thrombosis"[MeSH Terms] OR "thrombosis"[All Fields] OR "thrombus"[All Fields]))) OR ((("stable"[All Fields] OR "stabled"[All Fields] OR "stables"[All Fields] OR "stabling"[All Fields]) AND ("coronary artery disease"[MeSH Terms] OR ("coronary"[All Fields] AND "artery"[All Fields] AND "disease"[All Fields]) OR "coronary artery disease"[All Fields] OR ("angina pectoris"[MeSH Terms] OR ("angina"[All Fields] AND "pectoris"[All Fields]) OR "angina pectoris"[All Fields] OR "angina"[All Fields] OR "anginas"[All Fields]) OR ("angina pectoris"[MeSH Terms] OR ("angina"[All Fields] AND "pectoris"[All Fields]) OR "angina pectoris"[All Fields]))) OR "SCAD"[All Fields]) OR ("atherosclerosis"[MeSH Terms] OR "atherosclerosis"[All Fields] OR ("atherosclerotic"[All Fields] AND "cardiovascular"[All Fields] AND "disease"[All Fields]) OR "atherosclerotic cardiovascular disease"[All Fields] OR ("ascvd"[All Fields] OR "ascvds"[All Fields])) OR ((("chronic"[All Fields] OR "chronical"[All Fields] OR "chronically"[All Fields] OR "chronicities"[All Fields] OR "chronicity"[All Fields] OR "chronicization"[All Fields] OR "chronics"[All Fields]) AND ("coronaries"[All Fields] OR "heart"[MeSH Terms] OR "heart"[All Fields]	932

		OR "coronary"[All Fields]) AND ("syndrom"[All Fields] OR "syndromal"[All Fields] OR "syndromally"[All Fields] OR "syndrome"[MeSH Terms] OR "syndrome"[All Fields] OR "syndromes"[All Fields] OR "syndrome s"[All Fields] OR "syndromic"[All Fields] OR "syndroms"[All Fields])) OR ("cell commun signal"[Journal] OR "ccs"[All Fields]))) AND ("inflammation"[MeSH Terms] OR "inflammation"[All Fields] OR "inflammations"[All Fields] OR "inflammation s"[All Fields] OR (("inflammatories"[All Fields] OR "inflammatory"[All Fields]) AND "marker*"[All Fields]) OR ("curr res psychol"[Journal] OR "crp"[All Fields]) OR ("c reactive protein"[MeSH Terms] OR ("c reactive"[All Fields] AND "protein"[All Fields]) OR "c reactive protein"[All Fields] OR "c reactive protein"[All Fields]) OR ("interleukin 6"[MeSH Terms] OR "interleukin 6"[All Fields] OR "interleukin 6"[All Fields]) OR ("interleukin 6"[MeSH Terms] OR "interleukin 6"[All Fields] OR "il 6"[All Fields]) OR ("tumour necrosis factor alpha"[All Fields] OR "tumor necrosis factor alpha"[MeSH Terms] OR ("tumor"[All Fields] AND "necrosis"[All Fields] AND "factor alpha"[All Fields]) OR "tumor necrosis factor alpha"[All Fields] OR ("tumor"[All Fields] AND "necrosis"[All Fields] AND "factor"[All Fields] AND "alpha"[All Fields]) OR "tumor necrosis factor alpha"[All Fields]) OR "TNF-A"[All Fields] OR ("in-stent"[All Fields] AND "restenosis"[All Fields]) OR ("constriction, pathologic"[MeSH Terms] OR ("constriction"[All Fields] AND "pathologic"[All Fields]) OR "pathologic constriction"[All Fields] OR "stenosi"[All Fields] OR "stenosis"[All Fields]) OR (("acute"[All Fields] OR "acutely"[All Fields] OR "acutes"[All Fields]) AND ("thrombose"[All Fields] OR "thrombosing"[All Fields] OR "thrombosis"[MeSH Terms] OR "thrombosis"[All Fields] OR "thrombosed"[All Fields] OR "thromboses"[All Fields])) OR (("systemic"[All Fields] OR "systemically"[All Fields] OR "systemics"[All Fields]) AND ("inflammation"[MeSH Terms] OR "inflammation"[All Fields] OR "inflammations"[All Fields] OR "inflammation s"[All Fields])) OR ("post-procedure"[All Fields] AND "complication*"[All Fields]))) NOT ("editorial"[Publication Type] OR "editorial"[All Fields] OR ("letter"[Publication Type] OR "correspondence as topic"[MeSH Terms] OR "letter"[All Fields]) OR "comment*"[All Fields])) NOT ("animals"[MeSH Terms:noexp] OR "animal"[All Fields])) AND "English"[Language]	
5	#4 NOT animal	((("atherectomy"[MeSH Terms] OR "atherectomy"[All Fields] OR "atherectomies"[All Fields] OR ("atherectomy, coronary"[MeSH Terms] OR ("atherectomy"[All Fields] AND "coronary"[All Fields]) OR "coronary atherectomy"[All Fields] OR ("rotational"[All Fields] AND "atherectomy"[All Fields]) OR "rotational atherectomy"[All Fields])) AND ("coronary artery disease"[MeSH Terms] OR ("coronary"[All Fields] AND "artery"[All Fields] AND "disease"[All Fields]) OR "coronary artery disease"[All Fields] OR (("calcifiability"[All Fields] OR "calcifiable"[All Fields] OR "calcified"[All Fields] OR "calcifier"[All Fields] OR	1,026

		"calcifiers"[All Fields] OR "calcifies"[All Fields] OR "calcify"[All Fields] OR "calcifying"[All Fields]) AND ("coronary artery disease"[MeSH Terms] OR ("coronary"[All Fields] AND "artery"[All Fields] AND "disease"[All Fields]) OR "coronary artery disease"[All Fields] OR ("constriction, pathologic"[MeSH Terms] OR ("constriction"[All Fields] AND "pathologic"[All Fields]) OR "pathologic constriction"[All Fields] OR "stenosi"[All Fields] OR "stenosis"[All Fields]) OR ("thrombosis"[MeSH Terms] OR "thrombosis"[All Fields] OR "thrombus"[All Fields]))) OR ((("stable"[All Fields] OR "stabled"[All Fields] OR "stables"[All Fields] OR "stabling"[All Fields]) AND ("coronary artery disease"[MeSH Terms] OR ("coronary"[All Fields] AND "artery"[All Fields] AND "disease"[All Fields]) OR "coronary artery disease"[All Fields] OR ("angina pectoris"[MeSH Terms] OR ("angina"[All Fields] AND "pectoris"[All Fields]) OR "angina pectoris"[All Fields] OR "angina"[All Fields] OR "anginas"[All Fields]) OR ("angina pectoris"[MeSH Terms] OR ("angina"[All Fields] AND "pectoris"[All Fields]) OR "angina pectoris"[All Fields]))) OR "SCAD"[All Fields]) OR ("atherosclerosis"[MeSH Terms] OR "atherosclerosis"[All Fields] OR ("atherosclerotic"[All Fields] AND "cardiovascular"[All Fields] AND "disease"[All Fields]) OR "atherosclerotic cardiovascular disease"[All Fields] OR ("ascvd"[All Fields] OR "ascvds"[All Fields])) OR ((("chronic"[All Fields] OR "chronical"[All Fields] OR "chronically"[All Fields] OR "chronicities"[All Fields] OR "chronicity"[All Fields] OR "chronicization"[All Fields] OR "chronics"[All Fields]) AND ("coronaries"[All Fields] OR "heart"[MeSH Terms] OR "heart"[All Fields] OR "coronary"[All Fields]) AND ("syndrom"[All Fields] OR "syndromal"[All Fields] OR "syndromally"[All Fields] OR "syndrome"[MeSH Terms] OR "syndrome"[All Fields] OR "syndromes"[All Fields] OR "syndrome s"[All Fields] OR "syndromic"[All Fields] OR "syndroms"[All Fields])) OR ("cell commun signal"[Journal] OR "ccs"[All Fields]))) AND ("inflammation"[MeSH Terms] OR "inflammation"[All Fields] OR "inflammations"[All Fields] OR "inflammation s"[All Fields] OR (("inflammatories"[All Fields] OR "inflammatory"[All Fields]) AND "marker*"[All Fields]) OR ("curr res psychol"[Journal] OR "crp"[All Fields]) OR ("c reactive protein"[MeSH Terms] OR ("c reactive"[All Fields] AND "protein"[All Fields]) OR "c reactive protein"[All Fields] OR "c reactive protein"[All Fields]) OR ("interleukin 6"[MeSH Terms] OR "interleukin 6"[All Fields] OR "interleukin 6"[All Fields]) OR ("interleukin 6"[MeSH Terms] OR "interleukin 6"[All Fields] OR "il 6"[All Fields]) OR ("tumour necrosis factor alpha"[All Fields] OR "tumor necrosis factor alpha"[MeSH Terms] OR ("tumor"[All Fields] AND "necrosis"[All Fields] AND "factor alpha"[All Fields]) OR "tumor necrosis factor alpha"[All Fields] OR ("tumor"[All Fields] AND "necrosis"[All Fields] AND "factor"[All Fields] AND "alpha"[All Fields]) OR "tumor necrosis factor alpha"[All Fields]) OR "TNF-A"[All Fields] OR ("in-stent"[All Fields] AND "restenosis"[All Fields]) OR ("constriction, pathologic"[MeSH Terms] OR ("constriction"[All Fields] AND "pathologic"[All Fields]) OR "pathologic constriction"[All Fields] OR "stenosi"[All Fields] OR "stenosis"[All Fields]) OR (("acute"[All Fields] OR	

		"acutely"[All Fields] OR "acutes"[All Fields]) AND ("thrombose"[All Fields] OR "thrombosing"[All Fields] OR "thrombosis"[MeSH Terms] OR "thrombosis"[All Fields] OR "thrombosed"[All Fields] OR "thromboses"[All Fields])) OR (("systemic"[All Fields] OR "systemically"[All Fields] OR "systemics"[All Fields]) AND ("inflammation"[MeSH Terms] OR "inflammation"[All Fields] OR "inflammations"[All Fields] OR "inflammation s"[All Fields])) OR ("post-procedure"[All Fields] AND "complication*"[All Fields])))) NOT ("editorial"[Publication Type] OR "editorial"[All Fields] OR ("letter"[Publication Type] OR "correspondence as topic"[MeSH Terms] OR "letter"[All Fields]) OR "comment*"[All Fields])) NOT ("animals"[MeSH Terms:noexp] OR "animal"[All Fields])	
4	#1 AND #2 NOT (editorial OR letter OR comment*)	(("atherectomy"[MeSH Terms] OR "atherectomy"[All Fields] OR "atherectomies"[All Fields] OR ("atherectomy, coronary"[MeSH Terms] OR ("atherectomy"[All Fields] AND "coronary"[All Fields]) OR "coronary atherectomy"[All Fields] OR ("rotational"[All Fields] AND "atherectomy"[All Fields]) OR "rotational atherectomy"[All Fields])) AND ("coronary artery disease"[MeSH Terms] OR ("coronary"[All Fields] AND "artery"[All Fields] AND "disease"[All Fields]) OR "coronary artery disease"[All Fields] OR (("calcifiability"[All Fields] OR "calcifiable"[All Fields] OR "calcified"[All Fields] OR "calcifier"[All Fields] OR "calcifiers"[All Fields] OR "calcifies"[All Fields] OR "calcify"[All Fields] OR "calcifying"[All Fields]) AND ("coronary artery disease"[MeSH Terms] OR ("coronary"[All Fields] AND "artery"[All Fields] AND "disease"[All Fields]) OR "coronary artery disease"[All Fields] OR ("constriction, pathologic"[MeSH Terms] OR ("constriction"[All Fields] AND "pathologic"[All Fields]) OR "pathologic constriction"[All Fields] OR "stenosi"[All Fields] OR "stenosis"[All Fields]) OR ("thrombosis"[MeSH Terms] OR "thrombosis"[All Fields] OR "thrombus"[All Fields]))) OR ((("stable"[All Fields] OR "stabled"[All Fields] OR "stables"[All Fields] OR "stabling"[All Fields]) AND ("coronary artery disease"[MeSH Terms] OR ("coronary"[All Fields] AND "artery"[All Fields] AND "disease"[All Fields]) OR "coronary artery disease"[All Fields] OR ("angina pectoris"[MeSH Terms] OR ("angina"[All Fields] AND "pectoris"[All Fields]) OR "angina pectoris"[All Fields] OR "angina"[All Fields] OR "anginas"[All Fields]) OR ("angina pectoris"[MeSH Terms] OR ("angina"[All Fields] AND "pectoris"[All Fields]) OR "angina pectoris"[All Fields]))) OR "SCAD"[All Fields]) OR ("atherosclerosis"[MeSH Terms] OR "atherosclerosis"[All Fields] OR ("atherosclerotic"[All Fields] AND "cardiovascular"[All Fields] AND "disease"[All Fields]) OR "atherosclerotic cardiovascular disease"[All Fields] OR ("ascvd"[All Fields] OR "ascvds"[All Fields])) OR ((("chronic"[All Fields] OR "chronical"[All Fields] OR "chronically"[All Fields] OR "chronicities"[All Fields] OR "chronicity"[All Fields] OR "chronicization"[All Fields] OR "chronics"[All Fields]) AND ("coronaries"[All Fields] OR "heart"[MeSH Terms] OR "heart"[All Fields] OR "coronary"[All Fields]) AND ("syndrom"[All Fields] OR	1,065

		"syndromal"[All Fields] OR "syndromally"[All Fields] OR "syndrome"[MeSH Terms] OR "syndrome"[All Fields] OR "syndromes"[All Fields] OR "syndrome s"[All Fields] OR "syndromic"[All Fields] OR "syndroms"[All Fields])) OR ("cell commun signal"[Journal] OR "ccs"[All Fields]))) AND ("inflammation"[MeSH Terms] OR "inflammation"[All Fields] OR "inflammations"[All Fields] OR "inflammation s"[All Fields] OR (("inflammatories"[All Fields] OR "inflammatory"[All Fields]) AND "marker*"[All Fields]) OR ("curr res psychol"[Journal] OR "crp"[All Fields]) OR ("c reactive protein"[MeSH Terms] OR ("c reactive"[All Fields] AND "protein"[All Fields]) OR "c reactive protein"[All Fields] OR "c reactive protein"[All Fields]) OR ("interleukin 6"[MeSH Terms] OR "interleukin 6"[All Fields] OR "interleukin 6"[All Fields]) OR ("interleukin 6"[MeSH Terms] OR "interleukin 6"[All Fields] OR "il 6"[All Fields]) OR ("tumour necrosis factor alpha"[All Fields] OR "tumor necrosis factor alpha"[MeSH Terms] OR ("tumor"[All Fields] AND "necrosis"[All Fields] AND "factor alpha"[All Fields]) OR "tumor necrosis factor alpha"[All Fields] OR ("tumor"[All Fields] AND "necrosis"[All Fields] AND "factor"[All Fields] AND "alpha"[All Fields]) OR "tumor necrosis factor alpha"[All Fields]) OR "TNF-A"[All Fields] OR ("in-stent"[All Fields] AND "restenosis"[All Fields]) OR ("constriction, pathologic"[MeSH Terms] OR ("constriction"[All Fields] AND "pathologic"[All Fields]) OR "pathologic constriction"[All Fields] OR "stenosi"[All Fields] OR "stenosis"[All Fields]) OR (("acute"[All Fields] OR "acutely"[All Fields] OR "acutes"[All Fields]) AND ("thrombose"[All Fields] OR "thrombosing"[All Fields] OR "thrombosis"[MeSH Terms] OR "thrombosis"[All Fields] OR "thrombosed"[All Fields] OR "thromboses"[All Fields])) OR (("systemic"[All Fields] OR "systemically"[All Fields] OR "systemics"[All Fields]) AND ("inflammation"[MeSH Terms] OR "inflammation"[All Fields] OR "inflammations"[All Fields] OR "inflammation s"[All Fields])) OR ("post-procedure"[All Fields] AND "complication*"[All Fields]))) NOT ("editorial"[Publication Type] OR "editorial"[All Fields] OR ("letter"[Publication Type] OR "correspondence as topic"[MeSH Terms] OR "letter"[All Fields]) OR "comment*"[All Fields])	
3	#1 AND #2	("atherectomy"[MeSH Terms] OR "atherectomy"[All Fields] OR "atherectomies"[All Fields] OR ("atherectomy, coronary"[MeSH Terms] OR ("atherectomy"[All Fields] AND "coronary"[All Fields]) OR "coronary atherectomy"[All Fields] OR ("rotational"[All Fields] AND "atherectomy"[All Fields]) OR "rotational atherectomy"[All Fields])) AND ("coronary artery disease"[MeSH Terms] OR ("coronary"[All Fields] AND "artery"[All Fields] AND "disease"[All Fields]) OR "coronary artery disease"[All Fields] OR (("calcifiability"[All Fields] OR "calcifiable"[All Fields] OR "calcified"[All Fields] OR "calcifier"[All Fields] OR "calcifiers"[All Fields] OR "calcifies"[All Fields] OR "calcify"[All Fields] OR "calcifying"[All Fields]) AND ("coronary artery disease"[MeSH Terms] OR ("coronary"[All Fields] AND "artery"[All Fields] AND "disease"[All Fields])	1,085

		OR "coronary artery disease"[All Fields] OR ("constriction, pathologic"[MeSH Terms] OR ("constriction"[All Fields] AND "pathologic"[All Fields]) OR "pathologic constriction"[All Fields] OR "stenosi"[All Fields] OR "stenosis"[All Fields]) OR ("thrombosis"[MeSH Terms] OR "thrombosis"[All Fields] OR "thrombus"[All Fields]))) OR ((("stable"[All Fields] OR "stabled"[All Fields] OR "stables"[All Fields] OR "stabling"[All Fields]) AND ("coronary artery disease"[MeSH Terms] OR ("coronary"[All Fields] AND "artery"[All Fields] AND "disease"[All Fields]) OR "coronary artery disease"[All Fields] OR ("angina pectoris"[MeSH Terms] OR ("angina"[All Fields] AND "pectoris"[All Fields]) OR "angina pectoris"[All Fields] OR "angina"[All Fields] OR "anginas"[All Fields]) OR ("angina pectoris"[MeSH Terms] OR ("angina"[All Fields] AND "pectoris"[All Fields]) OR "angina pectoris"[All Fields]))) OR "SCAD"[All Fields]) OR ("atherosclerosis"[MeSH Terms] OR "atherosclerosis"[All Fields] OR ("atherosclerotic"[All Fields] AND "cardiovascular"[All Fields] AND "disease"[All Fields]) OR "atherosclerotic cardiovascular disease"[All Fields] OR ("ascvd"[All Fields] OR "ascvds"[All Fields])) OR ((("chronic"[All Fields] OR "chronical"[All Fields] OR "chronically"[All Fields] OR "chronicities"[All Fields] OR "chronicity"[All Fields] OR "chronicization"[All Fields] OR "chronics"[All Fields]) AND ("coronaries"[All Fields] OR "heart"[MeSH Terms] OR "heart"[All Fields] OR "coronary"[All Fields]) AND ("syndrom"[All Fields] OR "syndromal"[All Fields] OR "syndromally"[All Fields] OR "syndrome"[MeSH Terms] OR "syndrome"[All Fields] OR "syndromes"[All Fields] OR "syndrome s"[All Fields] OR "syndromic"[All Fields] OR "syndroms"[All Fields])) OR ("cell commun signal"[Journal] OR "ccs"[All Fields]))) AND ("inflammation"[MeSH Terms] OR "inflammation"[All Fields] OR "inflammations"[All Fields] OR "inflammation s"[All Fields] OR (("inflammatories"[All Fields] OR "inflammatory"[All Fields]) AND "marker*"[All Fields]) OR ("curr res psychol"[Journal] OR "crp"[All Fields]) OR ("c reactive protein"[MeSH Terms] OR ("c reactive"[All Fields] AND "protein"[All Fields]) OR "c reactive protein"[All Fields] OR "c reactive protein"[All Fields]) OR ("interleukin 6"[MeSH Terms] OR "interleukin 6"[All Fields] OR "interleukin 6"[All Fields]) OR ("interleukin 6"[MeSH Terms] OR "interleukin 6"[All Fields] OR "il 6"[All Fields]) OR ("tumour necrosis factor alpha"[All Fields] OR "tumor necrosis factor alpha"[MeSH Terms] OR ("tumor"[All Fields] AND "necrosis"[All Fields] AND "factor alpha"[All Fields]) OR "tumor necrosis factor alpha"[All Fields] OR ("tumor"[All Fields] AND "necrosis"[All Fields] AND "factor"[All Fields] AND "alpha"[All Fields]) OR "tumor necrosis factor alpha"[All Fields]) OR "TNF-A"[All Fields] OR ("in-stent"[All Fields] AND "restenosis"[All Fields]) OR ("constriction, pathologic"[MeSH Terms] OR ("constriction"[All Fields] AND "pathologic"[All Fields]) OR "pathologic constriction"[All Fields] OR "stenosi"[All Fields] OR "stenosis"[All Fields]) OR (("acute"[All Fields] OR "acutely"[All Fields] OR "acutes"[All Fields]) AND ("thrombose"[All Fields] OR "thrombosing"[All Fields] OR "thrombosis"[MeSH Terms] OR "thrombosis"[All Fields] OR "thrombosed"[All Fields] OR	

		"thromboses"[All Fields])) OR (("systemic"[All Fields] OR "systemically"[All Fields] OR "systemics"[All Fields]) AND ("inflammation"[MeSH Terms] OR "inflammation"[All Fields] OR "inflammations"[All Fields] OR "inflammation s"[All Fields])) OR ("post-procedure"[All Fields] AND "complication*"[All Fields]))	
2	(Inflammation OR Inflammatory marker* OR CRP OR C-reactive protein OR Interleukin-6 OR IL-6 OR Tumor necrosis factor alpha OR TNF-A OR in-stent restenosis OR stenosis OR acute thrombosis OR systemic inflammation OR post-procedure complication*)	"inflammation"[MeSH Terms] OR "inflammation"[All Fields] OR "inflammations"[All Fields] OR "inflammation s"[All Fields] OR (("inflammatories"[All Fields] OR "inflammatory"[All Fields]) AND "marker*"[All Fields]) OR ("curr res psychol"[Journal] OR "crp"[All Fields]) OR ("c reactive protein"[MeSH Terms] OR ("c reactive"[All Fields] AND "protein"[All Fields]) OR "c reactive protein"[All Fields] OR "c reactive protein"[All Fields]) OR ("interleukin 6"[MeSH Terms] OR "interleukin 6"[All Fields] OR "interleukin 6"[All Fields]) OR ("interleukin 6"[MeSH Terms] OR "interleukin 6"[All Fields] OR "il 6"[All Fields]) OR ("tumour necrosis factor alpha"[All Fields] OR "tumor necrosis factor alpha"[MeSH Terms] OR ("tumor"[All Fields] AND "necrosis"[All Fields] AND "factor alpha"[All Fields]) OR "tumor necrosis factor alpha"[All Fields] OR ("tumor"[All Fields] AND "necrosis"[All Fields] AND "factor"[All Fields] AND "alpha"[All Fields]) OR "tumor necrosis factor alpha"[All Fields]) OR "TNF-A"[All Fields] OR ("in-stent"[All Fields] AND "restenosis"[All Fields]) OR ("constriction, pathologic"[MeSH Terms] OR ("constriction"[All Fields] AND "pathologic"[All Fields]) OR "pathologic constriction"[All Fields] OR "stenosi"[All Fields] OR "stenosis"[All Fields]) OR (("acute"[All Fields] OR "acutely"[All Fields] OR "acutes"[All Fields]) AND ("thrombose"[All Fields] OR "thrombosing"[All Fields] OR "thrombosis"[MeSH Terms] OR "thrombosis"[All Fields] OR "thrombosed"[All Fields] OR "thromboses"[All Fields])) OR (("systemic"[All Fields] OR "systemically"[All Fields] OR "systemics"[All Fields]) AND ("inflammation"[MeSH Terms] OR "inflammation"[All Fields] OR "inflammations"[All Fields] OR "inflammation s"[All Fields])) OR ("post-procedure"[All Fields] AND "complication*"[All Fields])	1,616,622
1	(Atherectomy OR rotational atherectomy) AND (Coronary Artery Disease OR (calcified (coronary artery disease OR stenosis OR thrombus)) OR (stable (coronary artery disease OR	("atherectomy"[MeSH Terms] OR "atherectomy"[All Fields] OR "atherectomies"[All Fields] OR ("atherectomy, coronary"[MeSH Terms] OR ("atherectomy"[All Fields] AND "coronary"[All Fields]) OR "coronary atherectomy"[All Fields] OR ("rotational"[All Fields] AND "atherectomy"[All Fields]) OR "rotational atherectomy"[All Fields])) AND ("coronary artery disease"[MeSH Terms] OR ("coronary"[All Fields] AND "artery"[All Fields] AND "disease"[All Fields]) OR "coronary artery disease"[All Fields] OR (("calcifiability"[All Fields] OR "calcifiable"[All Fields] OR "calcified"[All Fields] OR "calcifier"[All Fields] OR "calcifiers"[All Fields] OR "calcifies"[All Fields] OR "calcify"[All Fields] OR "calcifying"[All Fields]) AND ("coronary artery disease"[MeSH Terms] OR ("coronary"[All Fields] AND "artery"[All Fields] AND "disease"[All Fields]) OR "coronary artery disease"[All Fields] OR ("constriction,	2,812

	angina OR angina pectoris) OR SCAD) OR (atherosclerotic cardiovascular disease OR ASCVD) OR (chronic coronary syndrome OR CCS))	pathologic"[MeSH Terms] OR ("constriction"[All Fields] AND "pathologic"[All Fields]) OR "pathologic constriction"[All Fields] OR "stenosi"[All Fields] OR "stenosis"[All Fields]) OR ("thrombosis"[MeSH Terms] OR "thrombosis"[All Fields] OR "thrombus"[All Fields]))) OR ((("stable"[All Fields] OR "stabled"[All Fields] OR "stables"[All Fields] OR "stabling"[All Fields]) AND ("coronary artery disease"[MeSH Terms] OR ("coronary"[All Fields] AND "artery"[All Fields] AND "disease"[All Fields]) OR "coronary artery disease"[All Fields] OR ("angina pectoris"[MeSH Terms] OR ("angina"[All Fields] AND "pectoris"[All Fields]) OR "angina pectoris"[All Fields] OR "angina"[All Fields] OR "anginas"[All Fields]) OR ("angina pectoris"[MeSH Terms] OR ("angina"[All Fields] AND "pectoris"[All Fields]) OR "angina pectoris"[All Fields]))) OR "SCAD"[All Fields]) OR ("atherosclerosis"[MeSH Terms] OR "atherosclerosis"[All Fields] OR ("atherosclerotic"[All Fields] AND "cardiovascular"[All Fields] AND "disease"[All Fields]) OR "atherosclerotic cardiovascular disease"[All Fields] OR ("ascvd"[All Fields] OR "ascvds"[All Fields])) OR ((("chronic"[All Fields] OR "chronical"[All Fields] OR "chronically"[All Fields] OR "chronicities"[All Fields] OR "chronicity"[All Fields] OR "chronicization"[All Fields] OR "chronics"[All Fields]) AND ("coronaries"[All Fields] OR "heart"[MeSH Terms] OR "heart"[All Fields] OR "coronary"[All Fields]) AND ("syndrom"[All Fields] OR "syndromal"[All Fields] OR "syndromally"[All Fields] OR "syndrome"[MeSH Terms] OR "syndrome"[All Fields] OR "syndromes"[All Fields] OR "syndrome s"[All Fields] OR "syndromic"[All Fields] OR "syndroms"[All Fields])) OR ("cell commun signal"[Journal] OR "ccs"[All Fields])))	

Table S1(b). Searching strategy on EMBASE

ID	Query	Results	Date
#6	(('inflammation'/exp OR 'inflammation marker':ti,ab OR 'inflammatory marker':ti,ab OR crp:ti,ab OR 'c-reactive protein':ti,ab OR 'interleukin-6':ti,ab OR 'il-6':ti,ab OR 'tumor necrosis factor alpha':ti,ab OR 'tnf-alpha':ti,ab OR 'in-stent restenosis':ti,ab OR stenosis:ti,ab OR (acute:ti,ab AND thrombosis:ti,ab) OR (systemic:ti,ab AND inflammation:ti,ab) OR (post:ti,ab AND procedural:ti,ab AND complication:ti,ab) OR macce:ti,ab OR mace:ti,ab OR (major:ti,ab AND adverse:ti,ab AND cardiovascular:ti,ab AND event$:ti,ab)) AND (percutaneous:ti,ab AND coronary:ti,ab AND 'intervention'/exp OR 'percutaneous coronary intervention':ti,ab OR 'percutaneous transluminal coronary angioplasty':ti,ab OR ptca:ti,ab OR 'balloon angioplasty':ti,ab) AND (('atherectomy'/exp OR atherectomy:ti,ab OR 'rotational atherectomy': ti,ab) AND (coronary:ti,ab AND artery:ti,ab AND 'disease'/exp OR 'coronary artery disease':ti,ab OR (('calcified':ti,ab OR 'stable':ti,ab) AND (coronary:ti,ab AND artery:ti,ab AND disease:ti,ab OR stenosis:ti,ab OR thrombus:ti,ab)) OR (angina:ti,ab AND pectoris:ti,ab) OR (stable:ti,ab AND angina:ti,ab) OR scad:ti,ab OR (atherosclerotic:ti,ab AND 'cardiovascular disease':ti,ab) OR ascvd:ti,ab OR (chronic:ti,ab AND coronary:ti,ab AND syndrome:ti,ab) OR ccs:ti,ab))) NOT (editor$ OR comment$ OR letter OR 'case report' OR 'case reports')	615	15 Feb 2025
#5	(('inflammation'/exp OR 'inflammation marker':ti,ab OR 'inflammatory marker':ti,ab OR crp:ti,ab OR 'c-reactive protein':ti,ab OR 'interleukin-6':ti,ab OR 'il-6':ti,ab OR 'tumor necrosis factor alpha':ti,ab OR 'tnf-alpha':ti,ab OR 'in-stent restenosis':ti,ab OR stenosis:ti,ab OR (acute:ti,ab AND thrombosis:ti,ab) OR (systemic:ti,ab AND inflammation:ti,ab) OR (post:ti,ab AND procedural:ti,ab AND complication:ti,ab) OR macce:ti,ab OR mace:ti,ab OR (major:ti,ab AND adverse:ti,ab AND cardiovascular:ti,ab AND event$:ti,ab)) AND (percutaneous:ti,ab AND coronary:ti,ab AND 'intervention'/exp OR 'percutaneous coronary intervention':ti,ab OR 'percutaneous transluminal coronary angioplasty':ti,ab OR ptca:ti,ab OR 'balloon angioplasty':ti,ab) AND (('atherectomy'/exp OR atherectomy:ti,ab OR 'rotational atherectomy':ti,ab) AND (coronary:ti,ab AND artery:ti,ab AND 'disease'/exp OR 'coronary artery disease':ti,ab OR (('calcified':ti,ab OR 'stable':ti,ab) AND (coronary:ti,ab AND artery:ti,ab AND disease:ti,ab OR stenosis:ti,ab OR thrombus:ti,ab)) OR (angina:ti,ab AND pectoris:ti,ab) OR (stable:ti,ab AND angina:ti,ab) OR scad:ti,ab OR (atherosclerotic:ti,ab AND 'cardiovascular disease':ti,ab) OR ascvd:ti,ab OR (chronic:ti,ab AND coronary:ti,ab AND syndrome:ti,ab) OR ccs:ti,ab))) NOT (editor$ OR comment$ OR letter OR 'case report')	619	15 Feb 2025

#4	('inflammation'/exp OR 'inflammation marker':ti,ab OR 'inflammatory marker':ti,ab OR crp:ti,ab OR 'c-reactive protein':ti,ab OR 'interleukin-6':ti,ab OR 'il-6':ti,ab OR 'tumor necrosis factor alpha':ti,ab OR 'tnf-alpha':ti,ab OR 'in-stent restenosis':ti,ab OR stenosis:ti,ab OR (acute:ti,ab AND thrombosis:ti,ab) OR (systemic:ti,ab AND inflammation:ti,ab) OR (post:ti,ab AND procedural:ti,ab AND complication:ti,ab) OR macce:ti,ab OR mace:ti,ab OR (major:ti,ab AND adverse:ti,ab AND cardiovascular:ti,ab AND event$:ti,ab)) AND (percutaneous:ti,ab AND coronary:ti,ab AND 'intervention'/exp OR 'percutaneous coronary intervention':ti,ab OR 'percutaneous transluminal coronary angioplasty':ti,ab OR ptca:ti,ab OR 'balloon angioplasty':ti,ab) AND (('atherectomy'/exp OR atherectomy:ti,ab OR 'rotational atherectomy': ti,ab) AND (coronary:ti,ab AND artery:ti,ab AND 'disease'/exp OR 'coronary artery disease':ti,ab OR (('calcified':ti,ab OR 'stable':ti,ab) AND (coronary:ti,ab AND artery:ti,ab AND disease:ti,ab OR stenosis:ti,ab OR thrombus:ti,ab)) OR (angina:ti,ab AND pectoris:ti,ab) OR (stable:ti,ab AND angina:ti,ab) OR scad:ti,ab OR (atherosclerotic:ti,ab AND 'cardiovascular disease':ti,ab) OR ascvd:ti,ab OR (chronic:ti,ab AND coronary:ti,ab AND syndrome:ti,ab) OR ccs:ti,ab))	787	15 Feb 2025
#3	('atherectomy'/exp OR atherectomy:ti,ab OR 'rotational atherectomy':ti,ab) AND (coronary:ti,ab AND artery:ti,ab AND 'disease'/exp OR 'coronary artery disease':ti,ab OR (('calcified':ti,ab OR 'stable':ti,ab) AND (coronary:ti,ab AND artery:ti,ab AND disease:ti,ab OR stenosis:ti,ab OR thrombus:ti,ab)) OR (angina:ti,ab AND pectoris:ti,ab) OR (stable:ti,ab AND angina:ti,ab) OR scad:ti,ab OR (atherosclerotic:ti,ab AND 'cardiovascular disease':ti,ab) OR ascvd:ti,ab OR (chronic:ti,ab AND coronary:ti,ab AND syndrome:ti,ab) OR ccs:ti,ab)	2,910	15 Feb 2025
#2	percutaneous:ti,ab AND coronary:ti,ab AND 'intervention'/exp OR 'percutaneous coronary intervention':ti,ab OR 'percutaneous transluminal coronary angioplasty':ti,ab OR ptca:ti,ab OR 'balloon angioplasty':ti,ab	96,749	15 Feb 2025
#1	'inflammation'/exp OR 'inflammation marker':ti,ab OR 'inflammatory marker':ti,ab OR crp:ti,ab OR 'c-reactive protein':ti,ab OR 'interleukin-6':ti,ab OR 'il-6':ti,ab OR 'tumor necrosis factor alpha':ti,ab OR 'tnf-alpha':ti,ab OR 'in-stent restenosis':ti,ab OR stenosis:ti,ab OR (acute:ti,ab AND thrombosis:ti,ab) OR (systemic:ti,ab AND inflammation:ti,ab) OR (post:ti,ab AND procedural:ti,ab AND complication:ti,ab) OR macce:ti,ab OR mace:ti,ab OR (major:ti,ab AND adverse:ti,ab AND cardiovascular:ti,ab AND event$:ti,ab)	5,259,965	15 Feb 2025
