# Supplementary material for: Clinical and Inflammatory Outcomes of Rotational Atherectomy in Calcified Coronary Lesions: A Systematic Review and Meta-Analysis
Source: J Clin Med. 2025 Jul 31;14(15):5389. doi: 10.3390/jcm14155389 (PMC12347861; doi:10.3390/jcm14155389)
Supplement: Supplementary file 1 [file jcm-14-05389-s001.zip › jcm-3688480_Supplementary Materials (Table S3).pdf]

Table S3. Included studies in systematic review (n=110) and meta-analysis (n=36)

No.	First Author, Year	Study design and Period	Study Population	Sample size (n)	Follow-up (months)	Male (n,%)	Age (years)	CAD risk factors n (%)	Clinical pesentation n (%)	Comparisons	Outcomes measured	Events Rota (n/total)	Events Comparator (n/total)	Conclusions
1	Abdel-Wahab (ROTAXUS trial), 2013	RCT (August 2006-March 2010)	patients with complex calcified native coronary lesions	240	9 months	86 (72.3)	70.5 ± 8.2	HT: 106 (89.1) DM: 33 (27.7) Smoking: 24 (20.2)	All Stable CAD	stenting without RA	In-stent late lumen loss (LLL), angiographic and strategy success, binary restenosis, definite stent thrombosis, and major adverse cardiac events	Strategy success:111/120<br>binary restenosis: 14/120<br>definite stent thrombosis: 0.8%<br>MACE: 5/120	Strategy success: 100/120<br>binary restenosis: 14/120<br>definite stent thrombosis: 0%<br>MACE: 5/120	Balloon dilation with only provisional rotablation remains the default strategy for complex calcified lesions before DES implantation
2	Abdel-Wahab (PREPARE-CALC trial), 2018	RCT (September 2014-October 2017)	Patient with MI and severely calcified native coronary lesions undergoing PCI to a strategy of lesion preparation using MB or RA followed by DES implantation	200	9 months	77 (77%)	74.8±7.1	HT: 93 (93%) DM: 68 (68%) Smoking: 15 (15%)	UA: 8 (8%) Stable CAD	modified balloon group	Strategy success, in-stent LLL, in-segment LLL, binary restenosis, stent thrombosis, target vessel failure, all major cardiac events	Strategy success:98/100<br>Stent thrombosis: 0<br>stent failure: 1/100<br>Death: 2/100<br>TVR: 3/100<br>TLR: 2/100	Strategy success: 81/100<br>Stent thrombosis: 0<br>stent failure: 4/100<br>Death: 2/100<br>TVR: 8/100<br>TLR: 7/100	Lesion preparation with upfront RA before DES implantation is feasible in nearly all patients with complex calcified coronary lesions
3	Allali, 2020	RCT (September 2014-October 2017)	patients with severely calcified coronary artery disease	104	9 months	47 (77%)	74.8 ± 6.5	HT: 58 (96%) DM: 21 (34%) Smoking: 9 (15%)	All Stable CAD	scoring/cutting balloon vs RA	Any significant stenosis, dissection, or thrombolysis in myocardial	Large dissection (>5 mm): 1/61<br>Stent failure: 4/61<br>Death: 0<br>stent thrombosis: 0	Large dissection (>5 mm): 6/43<br>Stent failure: 0<br>Death: 0<br>stent thrombosis: 0<br>Final TIMI flow< III: 0	side branch compromise was more frequently observed after lesion preparation

No.	First Author, Year	Study design and Period	Study Population	Sample size (n)	Follow-up (months)	Male (n,%)	Age (years)	CAD risk factors n (%)	Clinical pesentation n (%)	Comparisons	Outcomes measured	Events Rota (n/total)	Events Comparator (n/total)	Conclusions
			amenable to PCI								infarction flow <3.	Final TIMI flow< III: 1/61		with SCB as compared with RA
4	Dietz, 2001	RCT (May 1992-May 1996)	Patients with angiographically documented coronary artery disease and symptoms of stable angina pectoris or evidence of myocardial ischemia in the target lesion	452	6 months	73%	61 ± 9	HT: NR, DM: 18%<br>Smoking: NR	All Stable CAD	PTCR vs PCTA	Clinical and angiographic outcome	TLR: 54/214<br>Long-term success: 115/233<br>Restenosis: 54/147 (37%)	TLR: 59/206<br>LTS: 108/235<br>Restenosis: 51/146 (35%)	RA yields a higher rate of procedural success than balloon angioplasty without stents in highly complex lesions
5	Filho, 2005	RCT (2003)	CAD patients confirmed through coronario-graphy at Hemodynamics Service of InCor - HCFMUSP	22	18 months	54.5%	60 ± 11.9	HT: 19 (86.4)<br>DM: 8 (36.4)<br>smoking: 12 (54.5)	All Stable CAD	rotablator vs. balloon angioplasty (BA)	Intracoronary release of inflammatory markers (IM) after pPCI, i.e. cytokines TNF-a, IL-6 and IL-1 and the soluble adhesion molecules ICAM-1, E-selectin and P-selectin (analyzed by using ELISA)	Rota (n=11)<br>Delta<br>TNF-alfa: 0.40 ± 0.78<br>IL-6: 1.18 ± 0.98<br>IL-1: -0.05 ± 0.05<br>ICAM: -13.74 ± 15.7<br>E-Selectin: -2.40 ± 5.1<br>P-Selectin: -3.46 ± 14	Non-RA (n=11)<br>Delta<br>TNF-alfa: 0.49 ± 0.53<br>IL-6: 0.65 ± 0.82<br>IL-1: 0.04 ± 0.12<br>ICAM: -23.94 ± 34.56<br>E-Selectin:-1.20 ± 7.37<br>P-Selectin:3.59 ± 14.65	-Early after PCI, there is an increase of intracoronary concentrations of TNF-a & IL-6<br>-No significant difference between inflammatory markers released in coronary flow through rotablator and BA
6	Guerin, 1995	RCT (April 1992 -	Patient with type B2 lesion	Total : 64 patients<br>RA : 32	6 months	Total : 48 (75%)<br>RA : 25 (78,1%)	RA : 64.6 ± 10.8 (39-78)	NR	AMI : 17 (26,6%)<br>UA : 28 (43,7%)	RA with adjunctive balloon angioplasty	Procedural outcome and hospital complications,	Procedural Technical failure: 1/32<br>Death : 0/32	Procedural Outcome Technical failure with crossover : 1/32<br>Death : 0/32	In type B2 lesions, RA with systematic

No.	First Author, Year	Study design and Period	Study Population	Sample size (n)	Follow-up (months)	Male (n,%)	Age (years)	CAD risk factors n (%)	Clinical pesentation n (%)	Comparisons	Outcomes measured	Events Rota (n/total)	Events Comparator (n/total)	Conclusions
		September 1993)		patients PTCA : 32 patients		PTCA : 23 (71,9%)	PTCA : 63.3 ± 10.4 (38-79)			vs. conventional PTCA	balloon inflation time and pressure, restenosis rate, and duration of hospital stay	Emergency CABG : 0/32<br>Q-wave MI : 1/32<br>Minor complication : 8/32<br><br>Balloon inflation time and pressure<br>Total inflation time : 6.4 ± 2.6 min<br>Maximal inflation pressure : 6.6 ± 2.5 bar<br>Restenosis rate : 11/28<br>LoS : 8.5 ± 6.5 days	Emergency CABG : 2/32<br>Q-wave MI : 1/32<br>Minor complication : 0/32<br><br>Balloon inflation time and pressure<br>Total inflation time : 11.7 ± 7.4 min<br>Maximal inflation pressure : 4.0 ± 1.3 bar<br>Restenosis rate : 12/30<br>LoS : 9.1 ± 6.2 days	balloon angi-oplasty does not seem to increase procedural success, and the restenosis rate remains comparable
7	Jurado-Román (ROLLER COASTR-EPIC22 trial), 2025	Multi-center RCT, (July 2019 - Dec 2023)	Patients with moderate to severe calcified coronary lesions undergoing PCI	171 patients (57 RA, 57 IVL, 57 ELCA)	12 months	132 (77.2%)	70.9 ± 8.2	HT: 44 (77.2%) RA, 44 (78.6%) IVL, 48 (84.2%) ELCA DM: 26 (45.6%) RA, 27 (48.2%) IVL, 31 (54.4%) ELCA Smoking: 18 (32.1%) RA, 14 (25%) IVL, 15 (26.3%) ELCA	Stable CAD: 110 (64.3%) ACS (NSTEMI/UA): 61 (35.7%)	RA vs. IVL vs. ELCA	Stent expansion (primary), procedural success, clinical outcomes	MACE: Not specified<br>Death: 0/57<br>Stroke: 0/57<br>Stent Thrombosis: 0/57	IVL Group:<br>Death: 0/57,<br>Stroke: 0/57,<br>Stent Thrombosis: 0/57,<br>TLR: 0/57<br><br>ELCA Group:<br>Death: 1/57,<br>Stroke: 0/57,<br>Stent Thrombosis: 0/57,<br>TLR: 0/57	IVL was noninferior to RA for stent expansion. ELCA did not reach noninferiority margin. No significant differences in procedural success, MSA, or complications, though IVL had numerically fewer complications.

No.	First Author, Year	Study design and Period	Study Population	Sample size (n)	Follow-up (months)	Male (n,%)	Age (years)	CAD risk factors n (%)	Clinical pesentation n (%)	Comparisons	Outcomes measured	Events Rota (n/total)	Events Comparator (n/total)	Conclusions
8	Kini, 2021	RCT<br>(May 2017 - March 2019)	Patient with calcified bifurcation lesions	71 patients (71 lesions)<br>RA : 35 patients<br>CBA : 36 patients	In hospital	Total : 50 (70.1%)<br>RA group : 28 (80%)<br>CBA group : 22 (61,1%)	Total : 67.2 ± 10.1<br>RA group : 67.8 ± 10.5<br>CBA group : 66.6 ± 9.9	HT: 81,7%<br>DM: 36,6%<br>Smoking: 8,5%	MI: 14,1%	Rotational atherectomy vs. cutting balloon angioplasty (CBA)	Side branch (SB) compromise defined as SB diameter stenosis ≥70%, SB dissection or TIMI flow grade <3<br><br>SB fractional flow reserve (FFR) assessment	SB compromise : 7/35<br>SB DS >70% : 2/35<br>SB Dissect: 1/70<br>TIMI flow grade <3 : 3/35<br>SB FFR : 0.84 ± 0.08<br>SB FFR <0.80 : 7/28	SB compromise : 9/36<br>SB DS >70% : 4/36<br>SB Dissect: 0/36<br>TIMI flow grade <3 : 5/36<br>SB FFR : 0.83 ± 0.08<br>SB FFR <0.80 : 10/27	The rates of SB compromise and functionally significant SB stenosis after provisional stenting of calcified bifurcation lesions were not statistically significant in patients treated with RA and CBA.
9	Li & Guo, 2019	RCT<br>(January 2016 - January 2017)	Patients with CAD and coronary artery calcification	86	in-hospital	59.3%	67.1 ± 6.1	HT: 51 (59.3)<br>DM: 13 (15.1)<br>dyslipidemia: 68 (79.1)		Coronary rotational atherectomy before PCI vs. directly underwent PCI (control)	Serum inflammatory factors (i.e. IL-6 IL-18), and plaque stabilization factor (i.e. pentraxin 3 (PTX3), and lipoprotein-associated phospholipase A2 (Lp-PLA2))<br>- perioperative complications (malignant arrhythmia, AMI, coronary artery dissection and perforation), and postoperative	RA (n=43)<br><br>delta<br>IL-6 (pg/ml): 10 ± 2<br>IL-18 (pg/ml): 6<br>PTX3 (ng/ml): 2.03 ± 0.02<br>Lp-PLA2 (ng/ml): 4.58 ± 0.61<br><br>malignant arrhythmia: 2/43<br>MI: 1/43<br>coronary dissection: 0/43<br>coronary perforation: 1/43	Non-RA (n=43)<br><br>delta<br>IL-6 (pg/ml): 6 ± 1.5<br>IL-18 (pg/ml): 3<br>PTX3 (ng/ml): 1.39 ± 0.08<br>Lp-PLA2 (ng/ml): 4.58 ± 0.61<br><br>malignant arrhythmia: 4/43<br>MI: 2/43<br>coronary dissection: 2/43<br>coronary perforation: 2/43	Coronary rotational atherectomy may reduce serum inflammatory factors and plaque stabilization factors to some extent. It is confirmed the clinical effectiveness of coronary rotational atherectomy in the treatment of CAD.

No.	First Author, Year	Study design and Period	Study Population	Sample size (n)	Follow-up (months)	Male (n,%)	Age (years)	CAD risk factors n (%)	Clinical pesentation n (%)	Comparisons	Outcomes measured	Events Rota (n/total)	Events Comparator (n/total)	Conclusions
											changes in renal function			
10	Liang , 2022	RCT	Patient with coronary artery calcification who underwent PCI	440 patients (220 intervention and 220 control)	9 months	NR	NR	NR	NR	Rotational artherectomy vs. control	In-stent late lumen loss, major adverse cardiac events, in-segment late lumen loss, binary restenosis, strategy success, procedural duration, and contrast amount	in-stent late lumen loss : 0.34 ± 0.52 mm<br>procedural duration : 76.3 ± 41.8<br>MACE 17.8%<br>in segment late lumen loss 0.28 ± 0.66<br>binary restenosis 7.3% in-stent; 7.7% in-segment<br>contrast vol: 215.5 ± 112.5 ml	in-stent late lumen loss : 0.24 ± 0.47 mm<br>procedural duration : 67.0 ± 38.8<br>MACE : 25.2%<br>in segment late lumen loss 0.17 ± 0.55<br>binary restenosis 8.2% in-stent; 9.0% in-segment<br>contrast vol: 203.7 ± 96.5 ml	In-stent late lumen loss and major adverse cardiac events at 9 months

No.	First Author, Year	Study design and Period	Study Population	Sample size (n)	Follow-up (months)	Male (n,%)	Age (years)	CAD risk factors n (%)	Clinical pesentation n (%)	Comparisons	Outcomes measured	Events Rota (n/total)	Events Comparator (n/total)	Conclusions
11	Reifart, 1997	RCT (October 1991- December 1993)	Patients with coronary artery disease	685 total patients: balloon angioplasty/PTCA (n=222), excimer laser angioplasty/ELCA (n=232), or rotational atherectomy/PTRA (n=231)	4-12 months	PTCA=180 (81%) ELCA=180 (78%) PTRA=184 (80%)	PTCA=62.5±9.5 ELCA=61.7±8.8 PTRA=61.6±10.0	DM PTCA= 36 (16) ELCA= 40 (17) PTRA=35 (15)	UA PTCA= 27 (12) ELCA= 38 (16) PTRA= 42 (18)<br><br>Stable PTCA= 159 (72) ELCA= 153 (67) PTRA= 148 (65)<br><br>Asymptomatic PTCA= 36 (16) ELCA= 41 (17) PTRA= 41 (17)	Balloon angioplasty (PTCA) vs. excimer laser angioplasty (ELCA) vs. rotational atherectomy (PTRA)	1. Major in-hospital complications (death, myocardial infarction, or CABG)<br><br>2. 0 to 360 days composite clinical endpoint (death, Q-wave myocardial infarction, CABG, or repeated angioplasty)<br><br>3. Restenosis rate	1. Major in-hospital complications:<br>Death=2/231<br>CABG =2/231<br>Myocardial infarction=8/231<br><br>2. Cumulative Clinical Events at 0 and 360 Days:<br>Death=5/205<br>Q-wave MI=5/205<br>CABG=15/205<br>Repeated non surgical intervention=75/205<br>TVR =87/205<br>Any event=94/205<br><br>3. Restenosis=82/145	1.PTCA:<br>Death=2/222<br>CABG=1/222<br>Myocardial infarction=8/222<br><br>ELCA:<br>Death=2/232<br>CABG=5/232<br>Myocardial infarction=9/232<br><br>2. Cumulative Clinical Events at 0 and 360 Days:<br>PTCA:<br>Death=7/191<br>Q-wave MI=5/191<br>CABG=12/191<br>Repeated non surgical intervention=52/191<br>TVR=61/191<br>Any event=70/191<br><br>ELCA:<br>Death=4/211<br>Q-wave MI=5/211<br>CABG=15/211<br>Repeated non surgical intervention=85/211<br>TVR=97/211<br>Any event=101/211<br><br>3. Restenosis<br>PTCA: 51/109<br>ELCA: 85/143	Procedural success of rotational atherectomy is superior to laser angioplasty and balloon angioplasty; however, it does not result in better late outcomes.

No.	First Author, Year	Study design and Period	Study Population	Sample size (n)	Follow-up (months)	Male (n,%)	Age (years)	CAD risk factors n (%)	Clinical pesentation n (%)	Comparisons	Outcomes measured	Events Rota (n/total)	Events Comparator (n/total)	Conclusions
12	Sakakura, 2016	RCT	Patients with calcified coronary lesions undergoing rotational atherectomy	100	In hospital	80%	73±8	HT: 94%<br>DM: 47%<br>Smoking: 21%	N/R	Low-speed (140,000 rpm) vs. high-speed (190,000 rpm) rotational atherectomy	Slow flow,<br>TIMI flow grade,<br>MI	Low speed (n=50)<br>Slow flow: 24%<br>TIMI flow:<br>TIMI 3: 76%,<br>TIMI 2: 2.14%,<br>TIMI 1:1.8%,<br>TIMI 0: 0.2%<br>MI: 6%	High speed (n=50)<br>Slow flow: 24%<br>TIMI flow:<br>TIMI 3: 76%,<br>TIMI 2: 2.14%,<br>TIMI 1: 10%,<br>TIMI 0: 0%<br>MI: 6%	No significant difference in slow flow incidence between low-speed and high-speed RA. Other procedural outcomes were similar.
13	Sharma, 2004	RCT (January 1997 - December 1999.)	Patients with diffuse in-stent restenosis (ISR)	200	12 ± 2 months	74% (PRCA), 69% (PTCA)	63 ± 10 (PRCA), 65 ± 10 (PTCA)	HT: 77% (PRCA), 86% (PTCA)<br>DM: 31% (PRCA), 30% (PTCA)<br>Dyslipidemia: 63% (PRCA), 66% (PTCA)	not specified, only resting angina:<br>PRCA 20%<br>PTCA 24%	percutaneous rotational coronary atherectomy (PRCA) VS Conventional balloon angioplasty (PTCA)	Target lesion revascularization (TLR) at 9 months, Major adverse cardiac events (MACE: death, MI, or repeat target lesion intervention) at 1 year	PRCA group<br>TLR: 32/100<br>Death: 2/100<br>MI: 3/100<br>CABG: 3/100<br>MACE : 38%	PTCA group<br>TLR: 45/100<br>Death: 2/100<br>MI: 3/100<br>CABG: 3/100<br>MACE : 52%	Both PRCA and PTCA to be safe and effective, but PRCA re-sulted in less residual intimal hyperplasia, lower repeat stent use, and decreased TLR.
14	Sharma, 2024	RCT (May 2022-April 2023)	Patients with calcified lesions undergoing PCI assisted with RA	60	1 month	78%	71.1 ± 9.4	HT: RA+CBA (96.6%), RA+NCBA (93.5%)<br>DM: RA+CBA (41.4), RA+NCBA (41.9%)<br>Current Smokers: RA+CBA (11.1%), RA+NCBA (19.4%)	NR	RA+CBA (Rotational atherectomy +cutting balloon angioplasty), RA+NCBA (rotational atherectomy + non-compliant balloon angioplasty)	30-day MACE and major bleeding	RA+CBA<br>all-cause death: 0/29<br>MI: 2/29<br>TVR: 1/29<br>TLR: 0/29<br>Stent thrombosis:0<br>Major bleeding:0<br>Vacular complication: 0	RA + NCBA<br>all-cause death:0/31<br>MI:1/31<br>TVR:0<br>TLR:0<br>Stent thrombosis:0<br>Major bleeding:0<br>Vacular complication: 0	RA followed by CBA was safe with rare procedural complications and few clinical adverse events at 30 days.

No.	First Author, Year	Study design and Period	Study Population	Sample size (n)	Follow-up (months)	Male (n,%)	Age (years)	CAD risk factors n (%)	Clinical pesentation n (%)	Comparisons	Outcomes measured	Events Rota (n/total)	Events Comparator (n/total)	Conclusions
15	Zhang, 2015	RCT (January 2005 - December 2008)	Patients with nondilatable calcified lesions	198	48 months	89.4%	59.5 ± 10.5	HT: 57.6%<br>DM: 27.8%<br>Smoking:88.4 %<br>Dyslipidemia: 65.7%	Coronary artery dissection patient	RA vs Delayed RA (after dissection healing)	MACE (including all-cause death, non-fatal MI, stent thrombosis, cardiac tamponade, stroke, target lesion revascularization, and NYHA class IV heart failure).	RA (105):<br>All-cause death: 5/105<br>Cardiac death: 3/105<br>Non-fatal MI: 3/105<br>Stent thrombosis: 2/105<br>Cardiac tamponade: 1/105<br>Stroke: 2/105<br>TLR: 8/105<br>NYHA class IV heart failure: 2/105	Delayed RA (93):<br>All-cause death: 4/93<br>Cardiac death: 2/93<br>Non-fatal MI: 3/93<br>Stent thrombosis: 3/93<br>Cardiac tamponade: 1/93<br>Stroke: 2/93<br>TLR: 7/93<br>NYHA class IV heart failure: 2/93	This study indicates that immediate RA during PCI is safe and effective in patients with coronary artery dissection.
16	Barrett, 2020	P Cohort (2014-2018)	Patients who underwent PCI of a native coronary lesion with RA or OA	1091	30 days	1077 (98.7%)	70.3 ± 8.1	Smoking: 774 (70.9)	NR	Rotatory vs. orbital atherctomy	30-day MACCE, including all-cause mortality, repeat MI, target vessel revascularization (TVR), and stroke.	MACE: 51/640<br>death: 4/640<br>MI: 3/640<br>TVR: 43/640<br>dissect: 9/640<br>perforation: 5/640<br>slow/no flow: 4/640<br>stroke: 1/640	MACE: 38/451<br>death: 8/451<br>MI: 2/451<br>TVR: 32/451<br>dissect: 14/451<br>perforation: 9/451<br>slow/no flow: 7/451<br>stroke: 0/451	no significant difference in MACCE for patients who underwent RA or OA (7.1 vs. 8.2%, p = .36).<br>30-day mortality, MI, TVR, and stroke were also similar in the matched cohort.
17	Blachutzik, 2023	R Cohort (July 2019-November 2021)	patients with clinically significant and severely calcified coronary lesions	21	2 years	7 (77.8)	81.4 ± 6.9	HT: 9 (100)<br>DM: 3 (33.3)<br>Smoking: 1 (11.1)	STEMI: 0<br>NSTEMI: 1 (11.1) UA: 0<br>Stable CAD: 8 (88.9)	intravascular lithotripsy group	Acute effects of RA and IVL on severely calcified coronary stenoses using intravascular optical coherence	Plaque rupture: 0<br>Plaque erosion 1/9<br>Red thrombus 0<br>White thrombus 0<br>Dissection 0	Plaque rupture: 0<br>Plaque erosion 1/9<br>Red thrombus 0<br>White thrombus 1/12<br>Dissection 0	RA and IVL are both effective techniques for the modifi-cation of calcified coronary plaques.

No.	First Author, Year	Study design and Period	Study Population	Sample size (n)	Follow-up (months)	Male (n,%)	Age (years)	CAD risk factors n (%)	Clinical pesentation n (%)	Comparisons	Outcomes measured	Events Rota (n/total)	Events Comparator (n/total)	Conclusions
											tomography (OCT)			
18	Chambers, 2019	R Cohort (January 2010 - March 2016)	Patient with severely calcified coronary bifurcation lesion who underwent PCI	72	30 days	75%	70.6 ± 8.6	HT: 93,1%<br>DM: 44,4%<br>Smoking: 77,8%	NR	Rotational atherectomy (RA) vs. orbital atherectomy (OAS)	MACE, defined as a composite of death, MI, and TVR<br><br>Procedural results, defined as total procedure time, fluoroscopy time, and length of stay	MACE: 1/39<br>death: 1/39<br>MI: 0/39<br>TVR: 0/39<br>slow flow/no reflow: 1/39<br><br>Fluoro time: 27.4 ± 17.4<br>contrast vol: 237.9 ± 94.1	MACE: 2/33<br>death: 2/33<br>MI: 0/33<br>TVR: 1/33<br>slow flow/no reflow: 1/33<br><br>Fluoro time: 16.3 ± 7.6<br>contrast vol: 225.0 ± 82.5	There were no significant differences between OAS and RA subgroups regarding MACE rates (P>0.05)
19	Clavijo, 2006	R Cohort (April 2003-January 2005)	Patient underwent percutaneous coronary angioplasty with sirolimus-eluting stents required rotational atherectomy	150	6 months	54 (67.5%)	71.5 ± 9.6	HT: 67 (83.8%)<br>DM: 36 (44.4%)<br>Smoking: 42 (51.9%)	Stable CAD: 38 (46.9)<br>UA: 29 (35.8)	PCI without rotational atherectomy	Procedural success, MACCE including death, MI, and TLR	GPIIbIIIa use: 11/81<br>MACE: 8/72<br>Death: 5/72<br>Q-Wave MI: 1/72<br>TLR: 3/72	GPIIbIIIa use: 6/69<br>All MACE: 9/63<br>Death: 5/63<br>Q-Wave MI: 1/63<br>TLR: 3/63	Rotational atherectomy, when needed to modify calcified plaques and facilitate delivery of a SES, is safe, effective, and associated with TLR rates rivaling those of SES in noncalcified lesions

No.	First Author, Year	Study design and Period	Study Population	Sample size (n)	Follow-up (months)	Male (n,%)	Age (years)	CAD risk factors n (%)	Clinical pesentation n (%)	Comparisons	Outcomes measured	Events Rota (n/total)	Events Comparator (n/total)	Conclusions
20	Dahdouh et al, 2013	R Cohort (June 2004- December 2010)	Octogenarian patients who underwent PCI for highly calcified left main coronary artery	39	mean: 25.7± 21.4 months	8 (61.5%)	83.1 ± 2.3	HT: 9 (69.2%)<br>DM: 3 (23.0%)<br>Smoking: 1 (7.7%)	STEMI: 0<br>NSTEMI: 2 (16.7%)	PCI with vs. without rotational atherectomy	Procedural success, MACCE including death, nonfatal myocardial infarction, target lesion revascularization, or stroke	All cause of death: 1/12<br>Cardiac death: 1/12<br>HF: 5/12<br>Bleeding 0/12<br>TLR: 3/12<br>Stroke: 0/12	All cause of death: 8/27<br>Cardiac death: 8/27<br>HF: 5/27<br>Bleeding 0/12<br>TLR: 3/27<br>Stroke: 0/27	RA followed by stent implantation by transradial approach, when applied to heavily calcified lesions, appeared to be a safe and effective strategy for octogenarians who were refused for surgery
21	El Hajj, 2020	R Cohort (2016 - 2017)	patients with heavily calcified coronary disease who underwent RA or OA	74	12 months	74 (100%)	70.5 ± 7.0	HT: 69 (93.2)<br>DM: 54 (73.0<br>Smoking: 32 (43.2)	STEMI: 2 (2.7%)<br>NSTEMI: 10 (13.5%)<br>UA: 36 (48.6%)<br>SCAD: 8 (10.8%)	Rotatory vs. orbital atherctomy	The primary endpoint:<br>1-year MACCE (composite of all-cause mortality, MI, TVR, and stroke).<br><br>The secondary endpoint: 1-year cardiovascular death.	1-year<br>MACE: 12/46<br>death: 6/46<br>MI: 5/46<br>TVR: 6/46<br>dissect: 2/46<br>slow/no flow: 1/46<br>fluoro time: 26 ± 5.25<br>contrast vol: 125 ± 14.8	1-year<br>MACE: 3/28<br>death: 2/28<br>MI: 0/28<br>TVR: 2/28<br>dissect: 1/28<br>slow/no flow: 0/28<br>fluoro time: 27 ± 4.25<br>contrast vol: 150 ± 17.3	Both RA and OA are safe and effective in treating severe coronary artery calcification as<br>they provide similar outcomes at 1 year.

No.	First Author, Year	Study design and Period	Study Population	Sample size (n)	Follow-up (months)	Male (n,%)	Age (years)	CAD risk factors n (%)	Clinical pesentation n (%)	Comparisons	Outcomes measured	Events Rota (n/total)	Events Comparator (n/total)	Conclusions
22	Farhat, 2023	R Cohort (2019 - 2021)	Patient with calcified in-stent restenosis	Total : 52 patients<br>RA : 28 patients<br>IVL : 24 patients	12 months	All : 46 (88,5%)<br>RA : 26 (93%)<br>IVL : 20 (83%)	All : 74,1<br>RA : 72,9<br>IVL : 75,4	HT: 40 (76,9%)<br>DM: 25 (48,1%)<br>Smoking: 5 (9,6%)	N/STEMI : 16 (31%)<br>UA : 35 (3%)	Rotational atherectomy vs. intravascular lithotripsy	Procedural success, defined as residual stenosis <30% at the end of the procedure without presence of serious procedure-related complications<br><br>MACE, defined as the composite of death, MI, HF, and TVR	Procedural success : 100%<br><br>In hospital MACE : 1/28<br>In hospital death : 0/28<br><br>12-months<br>MACE : 4/28<br>MI : 1/28<br>TVR : 2/28<br>Death : 1/28	Procedural success : 100%<br><br>In hospital MACE : 0/24<br>In hospital death : 0/24<br><br>12-months<br>MACE : 4/24<br>MI : 1/24<br>TVR : 3/24<br>Death : 0/24	RA and IVL are safe and feasible for calcified ISR yielding comparable results at 1-year follow-up
23	Fujimoto, 2010	R Cohort (July 2004 - December 2008)	HD patients with calcified coronary lesions undergoing DES implantation	Total : 54 patients<br>PTCRA : 26 patients<br>Non-PTCRA : 28 patients	12 months	PTCRA : 20 (76.9%)<br>Non-PTCRA : 22 (78.6%)	PTCRA : 66.2 ± 8.1<br>Non-PTCRA : 65.0 ± 10.4	HT: 44 (81,5%)<br>DM: 35 (64,8%)<br>Smoking: 35 (64,8%)	NR	Percutaneous transluminal coronary rotational atherectomy (PTCRA) vs. non-PTCRA	MACE, defined as composite of death from cardiac causes, myocardial infarction, ischemia-driven TLR, and cerebrovascular events<br><br>Angiographical outcomes, including MLD, diametric stenosis, late loss, in-stent restenosis, target vessel	In-stent thrombosis : 0/26<br>Cardiac death : 1/26<br>MI : 0/26<br>Cerebrovascular event : 0/26<br>TLR : 3/26<br>**12-months**<br>MLD: 2.03 ± 0.98 mm<br>Diametric stenosis : 23.2 ± 35.7%<br>Late loss : 0.61 ± 0.98 mm<br>In-stent restenosis : 17.4%<br>Target vessel failure : 26.1%	In-stent thrombosis : 2/28<br>Cardiac death : 0/28<br>MI : 2/28<br>Cerebrovascular event : 0/28<br>TLR : 10/28<br>**12-months**<br>MLD : 1.51 ± 0.97 mm<br>Diametric stenosis : 40.9 ± 37.1%<br>Late loss : 1.05 ± 1.02 mm<br>In-stent restenosis : 42.3%<br>Target vessel failure : 46.2%<br>FU length : 7.9 ± 1.2 months	PTCRA may be useful for improving the mid-term outcome of DES implantation in hemodialysis patients with calcified lesions

No.	First Author, Year	Study design and Period	Study Population	Sample size (n)	Follow-up (months)	Male (n,%)	Age (years)	CAD risk factors n (%)	Clinical pesentation n (%)	Comparisons	Outcomes measured	Events Rota (n/total)	Events Comparator (n/total)	Conclusions
											failure, follow=up length	FU length : 7.3 ± 1.4 months		
24	Gallinoro, 2022	R Cohort (January 2016 - June 2020)	Patient with calcified lesions undergoing PCI	Total : 105 patients<br>RA : 70 patients<br>IVL : 35 patients	NA	All : 84 (80.0%)<br>RA : 55 (78.6%)<br>IVL : 29 (82.9%)	All : 73.28 ± 8.63<br>RA : 73.86 ± 8.83<br>IVL : 72.11 ± 8.22	HT: 71 (67.6%)<br>DM: 30 (28,6%)<br>Smoking: 41 (39.0%)	NSTEMI : 11 (10.5%)	Rotational atherectomy vs. intravascular lithotripsy	In-stent pressure gradients (vFFRgrad), percentage of vessel with a vFFRpost >0.90	Pre PCI vFFRgrad : 0.65 ± 0.13<br>Post PCI vFFRgrad : 0.043 ± 0.026<br>Percentage of vessel with a vFFRpost >0.90 : 32.9%	Pre PCI vFFRgrad : 0.67 ± 0.11<br>Post PCI vFFRgrad : 0.032 ± 0.026<br>Percentage of vessel with a vFFRpost >0.90 : 37.1%	Calcific lesions with IVL is effective and resulted in lower in-stent pressure gradients compared to RA
25	Hoffmann, 1998	R Cohort (February 1990-May 1996)	Patients with coronary lesions with the presence of of moderate or severe clacification	306 total patients:<br>RA = 147<br>Stent = 103<br>RA + Stent = 56	9 months	RA =103 (71%)<br>Stent =77 (75%)<br>RA + Stent =42 (75%)	RA =67 (10)<br>Stent =65 (10)<br>RA + Stent =67 (11)	DM<br>RA= 26 (17)<br>Stent= 28 (27)<br>RA + Stent =14 (25)<br><br>HT<br>RA= 84 (57)<br>Stent= 56 (54)<br>RA + Stent =30 (54)	UA<br>RA= 54 (37)<br>Stent= 47 (46)<br>RA + Stent =17 (30)	RA vs Stent vs RA + Stent	1. in-hospital MACE (death, myocardial infarction, or emergency coronary bypass surgery)<br><br>2. Follow up clinical outcomes late target lesion revascularization (CABG or coronary angioplasty), death, and myocardial infarctions	**RA only**<br><br>**in-hospital**<br>Death=0/147<br>Q-wave MI=1/147<br>Emergency CABG= 0/147<br><br>**Follow-up**<br>Death=2/147<br>MI=1/147<br>TLR = 41/147	**in-hospital**<br>**Stent:**<br>Death=1/103<br>Q-wave MI=1/103<br>CABG= 1/103<br>**RA + Stent:**<br>Death=1/56<br>Q-wave MI=1/56<br>Emergency CABG= 1/56<br><br>**Follow-up**<br>**Stent:**<br>Death=2/103<br>MI=0/103<br>TLR= 22/103<br><br>**RA + Stent:**<br>Death=0/56<br>MI=0/56<br>TLR= 8/56	Pre-athero ablation using RA followed by adjunct stent placement for calcified lesions in large arteries, is associated with infrequent complications, the largest acute angiographic results

No.	First Author, Year	Study design and Period	Study Population	Sample size (n)	Follow-up (months)	Male (n,%)	Age (years)	CAD risk factors n (%)	Clinical pesentation n (%)	Comparisons	Outcomes measured	Events Rota (n/total)	Events Comparator (n/total)	Conclusions
26	Januszek, 2017	R Cohort (January 2014 - December 2016)	Patient with heavility calcified coronary artery stenoses who undergo PCI	Total : 317 175 patients RA : 1 175 patients Non-RA : 316 010 patients	In hospital	RA : 801 (68.3%) Non-RA : 241 039 (76.4%)	RA : 71.7 ± 9.7 Non-RA : 66.9 ± 10.8	HT: 226 560 (71,4%) DM: 76 707 (24,2%) Smoking: 62 470 (19,7%)	STEMI : 74593 (23,5%) NSTEMI : 60846 (19,2%) UA : 94786 (29,9%) Stable CAD : 84157 (26,5%)	Rotational atherectomy vs. PCIs other than RA	Periprocedural complication, including MACCE, arterial dissection, CAP, cardiac arrest, death, MI, no reflow, cerebral stroke, and puncture site bleeding	All complication : 36/1139 MACCE : 7/1175 Arterial dissection : 2/557 CAP : 12/1163 Cardiac arrest : 9/1166 Death : 2/1173 MI : 2/1173 No-reflow : 8/1167 Cerebral stroke : 3/556 Puncture site bleeding : 2/1173	All complication : 5,778/310,222 MACCE : 1,863/316,010 Arterial dissection : 430/273,680 CAP : 527/315,483 Cardiac arrest : 1,389/314,621 Death : 1,540/314,470 MI : 313/315,697 No-reflow : 1,712/314,298 Cerebral stroke : 33/274,077 Puncture site bleeding : 300/315,710	
27	Koifman, 2018	R Cohort (2013 - 2016)	all patients who underwent percutaneous coronary intervention with severely calcified lesions	184	In hospital	125 (67.9%)	73.5 ± 10.5	HT: 176 (95.7) DM: 96 (52.2) Smoking: 78 (42.4)	NR	Rotatory vs. orbital atherctomy	The primary end point was the increase in troponin after the procedure. Secondary end points included procedural success, all-cause mortality, Q-wave MI, TVR, TLR, and stent thrombosis/	dissect: 1/115 procedure length: 109 ± 54 min residual stenosis: 19 (22-15)	dissect: 5/67 procedure length: 122 ± 52 min residual stenosis: 18 (25-17)	RAand OA have similar safety and efficacy profiles in treating patients with calcified coronary lesions, but higher rate of coronary dissections with OA compared with RA (p = 0.003).

No.	First Author, Year	Study design and Period	Study Population	Sample size (n)	Follow-up (months)	Male (n,%)	Age (years)	CAD risk factors n (%)	Clinical pesentation n (%)	Comparisons	Outcomes measured	Events Rota (n/total)	Events Comparator (n/total)	Conclusions
28	Lee, 2017	R cohort (2012 - 2016)	NSTE-ACS with severe calcified lesion	117	1 month	80 (68.4%)	62 ± 11	HT: 71%<br>DM: 38%	NSTEMI: 9%<br>UA: 23%	Rotatory vs. orbital atherctomy	30-day MACE defined as composite death, MI, TVR, and stroke	MACE: 4/67<br>death: 0/67<br>MI: 4/67<br>TVR: 0/67<br>dissect: 0/67<br>slow/no flow: 5/67<br>cardiac tamponade: 0/67<br>Perforation: 0/67	MACE: 3/50<br>death: 1/50<br>MI: 2/50<br>TVR: 0/50<br>dissection: 0/50<br>Slow/no flow: 2/50<br>cardiac tamponade: 1/50<br>Perforation: 1/50	both RA and OA are safe and effective for treatment of severe CAC as they provided similar clinical outcomes at short-term follow-up
29	Maier, 2024	R Cohort (2017 - 2020)	Patients with CAD with coronary plaques of superficial calcium	total : 32.470 patients<br>RA : 10.092 patients<br>MB : 22.378 patients	In hospital	Total : 24767 (76,27%)<br>RA : 7814 (77,4%)<br>MB : 16950 (75,7%)	RA : 74.23 ± 8.68<br>MB : 71.86 ± 10.02	HT: 82,5%<br>DM: 36,1%	NR	Rotational atherectomy vs modified balloon angioplasty (MB)	Safety outcomes (length of stay, MACCE, acute cerebrovascular events, pericardial effusion, and bleeding)	LoS: 6.41±7.9<br>MACCE: 228/10092 (2,26%)<br>Acute cerebrovascular events : 502/10092 (4.97%)<br>pericardial effusion: 194/10092 (1.92%)<br>bleeding : 80/10092 (0.79%)	LoS: 5.09±7.3<br>MACCE: 370/22378 (1.65%)<br>Acute cerebrovascular events : 1046/22378 (4.67%)<br>pericardial effusion: 208/22378 (0.93%)<br>bleeding : 122/22378 (0.55%)	Modified baloon had a lower risk of bleeding. Patients treated at centers with high annual RA procedure numbers had a lower risk of acute CVE (p<0.001) and were hospitalized shorter (p=0.005)
30	McGrew, 2016	R Cohort (June 2010 - July 2015)	Patient with calcified coronary artery disease who underwent PCI	total : 274 (RA, n= 147; OAS, n= 127)	6 months	NR	NR	NR	NR	Rotational atherectomy (RA) vs. orbital atherectomy (OAS)	1) Procedural success (successful atherectomy, TIMI flow post atherectomy), and 2) 6 months of angina free and MACE (TLR, TVR, MI,	**Procedural Success**<br>successful atherectomy : 131/147<br>TIMI 3 flow post atherectomy : 126/147<br>**6 months**<br>angina free :	**Procedural Success**<br>successful atherectomy : 114/127<br>TIMI 3 flow post atherectomy : 109/127<br>**6 months**<br>angina free : 81/106<br>MACE : 25/106	OAS and RA in contemporary practice are equally safe and effective for plaque modification in significantly calcified

No.	First Author, Year	Study design and Period	Study Population	Sample size (n)	Follow-up (months)	Male (n,%)	Age (years)	CAD risk factors n (%)	Clinical pesentation n (%)	Comparisons	Outcomes measured	Events Rota (n/total)	Events Comparator (n/total)	Conclusions
											and Death) incidence	94/118<br>MACE : 24/118		coronary artery lesions with high success rates
31	Meraj, 2018	P Cohort (2011-2017)	Patients from five tertiary care hospitals who had PCI	907	In hospital	592 (65.3%)	72.3 + 10.6	HT: 866 (95.5)<br>DM: 476 (52.4)<br>Smoking: 129 (14.2)	STEMI: 7 (0.8%)<br>NSTEMI: 118 (13%)<br>UA: 545 (60.1%)<br>SCAD: 192 (21.2%)	Rotatory (n=474) vs. orbital atherctomy (n=433), post matching, n=273 both groups	Primary end point was MI and safety outcomes included significant dissection, perforation, cardiac tamponade, and vascular complications.	MACE:<br>death: 6/273<br>MI: 35/273<br>dissect: 2/273<br>slow/no flow:<br>cardiac tamponade: 2/273<br>perforation: 2/273<br>Fluro time: 25.6 ± 13.3<br>Contrast vol: 150 ± 66.5	MACE:<br>death: 0/273<br>MI: 18/273<br>dissect: 3/273<br>slow/no flow:<br>cardiac tamponade: 1/273<br>perforation: 1/273<br>Fluro time: 21.9 ± 12.3<br>Contrast vol: 158.2 ± 66.4	OA was associated with significantly decreased in-hospital myocardial infarction and mortality after propensity score matching with decreased fluoroscopy time.

No.	First Author, Year	Study design and Period	Study Population	Sample size (n)	Follow-up (months)	Male (n,%)	Age (years)	CAD risk factors n (%)	Clinical pesentation n (%)	Comparisons	Outcomes measured	Events Rota (n/total)	Events Comparator (n/total)	Conclusions
32	Mousa, 2023	R Cohort (January 2006-June 2021)	Patients with balloon-crossable heavily calcified coronary lesions.	101 total patients<br>Rota 51<br>IVL 50	6 months	Total: 64 (63.4)<br>Rota:30 (59)<br>IVL: 34 (68)	Total: 73.3 ± 8.8<br>Rota: 72.8 ± 8.7<br>IVL: 73.7 ± 8.9	Rota<br>DM: 14 (27)<br>HT: 28 (55)<br><br>IVL<br>DM: 20 (40)<br>HT: 38 (76)	NR	Rotational Atherectomy (RA) vs. intravascular lithotripsy (IVL)	1. In hospital complications (death, procedure-related MI, stroke, coronary perforation, no reflow, vascular access complication, CABG)<br><br>2. MACE, defined as the composite of all-cause mortality, TLR, stroke and stent thrombosis (ST)	1. In-hospital follow up<br>Death: 1/51<br>Procedure-related MI: 1/51<br>Stroke: 1/51<br>Coronary perforation: 2/51<br>No reflow: 0/51<br>Vascular access complications: 1/51<br><br>2. 6-month follow-up<br>Total MACE: 6/51<br>Death: 4/51<br>TLR: 1/51<br>Stroke: 1/51<br>Stent thrombosis: 1/51	1. In hospital follow up<br>Death: 1/50<br>Procedure-related MI: 0/50<br>Stroke: 0/50<br>Coronary perforation: 1/50<br>No reflow: 0/50<br>Vascular access complications: 1/50<br><br>2. 6-month follow-up<br>Total MACE: 3/50<br>Death: 1/50<br>TLR: 1/50<br>Stroke: 1/50<br>Stent thrombosis: 0/50	Both IVL and RA are safe and effective methods for treatment of heavily calcified coronary lesions with similar outcomes at short term follow up
33	Okamoto, 2019	R Cohort (2013 - 2016)	Patients who underwent PCI treated with atherectomy for moderate to severely calcified lesions	1,149	1 year	826 (71.9%)	71.1 ± 10.5	HT: 1077 (93.7)<br>DM: 544 (47.3)	STEMI: 1 (0.09%)<br>NSTEMI: 91 (7.9%)<br>UA: 332 (28.9%)<br>SCAD: 664 (57.8%)	Rotatory vs. orbital atherctomy	Primary 1-year clinical outcome was MACE, defined as a composite of death, MI or TLR.	1-year<br>MACE: 209/965<br>death: 34/965<br>MI: 147/965<br>TVR: 68/965<br>dissect: 3/965<br>tamponade: 1/965<br>slow/no flow: 21/965<br>perforation: 2/965<br>Fluoro time: 28.7 ± 13.8<br>contrast vol: 156.4 ± 59.2	1-year<br>MACE: 30/184<br>death: 1/184<br>MI: 27/184<br>TVR: 5/184<br>dissect: 3/184<br>tamponade: 1/184<br>slow/no flow: 4/184<br>perforation: 3/184<br>Fluoro time: 25.5 ± 10.6<br>contrast vol: 147.9 ± 53.4	OA was associated with lower unadjusted but similar adjusted one-year MACE compared to RA with higher rates of dissection and device-induced perforation

No.	First Author, Year	Study design and Period	Study Population	Sample size (n)	Follow-up (months)	Male (n,%)	Age (years)	CAD risk factors n (%)	Clinical pesentation n (%)	Comparisons	Outcomes measured	Events Rota (n/total)	Events Comparator (n/total)	Conclusions
34	Rola, 2022	R Cohort (January 2014-June 2021)	Patients with calcified lesions who had undergone PCI	44 total patients<br>Rota 29<br>S-IVL 15	30 days and 6 months post-discharge	Rota: 21 (72.4%)<br>S-IVL: 12 (80.0%)	Rota: 70.3 ± 9.1<br>S-IVL: 72.1 ± 6.1	Rota<br>DM: 16 (55.2%)<br>HT: 25 (86.2%)<br><br>S-IVL<br>DM: 10 (66.7%)<br>HT: 13 (86.7%)	Rota<br>Stable: 13 (44.8%)<br>UA: 6 (20.7%)<br>NSTEMI: 9 (31.0%)<br>STEMI: 1 (3.4%)<br><br>S-IVL<br>Stable: 6 (40.0%)<br>UA: 2 (13.3%)<br>NSTEMI: 6 (40.0%)<br>STEMI: 1 (6.7%)	Rotational Atherectomy (RA) vs. Shockwave Intravascular (S-IVL)	1. In hospital MACE (death, MI, an urgent need for TVR, and probable or definite stent thrombosis)<br><br>2. 6-mo MACE (cerebrovascular episodes, all kinds of revascularization procedures, and scaffold restenosis)	1. In hospital<br>MACE: 3/29<br>Death: 3/29<br>MI: 0/29<br>TVR: 0/29<br>Stent thrombosis: 0/29<br><br>2. 6-month follow-up<br>MACE: 5/29<br>Death: 4/29<br>MI: 1/29<br>TVR: 0/29<br>Stent thrombosis: 0/29<br>Revascularization: 2/29	1. In hospital<br>MACE: 1/15<br>Death: 1/15<br>MI: 1/15<br>TVR: 1/15<br>Stent thrombosis: 1/15<br><br>2. 6-month follow-up<br>MACE: 2/15<br>Death: 2/15<br>MI: 1/15<br>TVR: 1/15<br>Stent thrombosis: 1/15<br>Any revascularization: 2/15	RA and S-IVL could be safe and effective therapeutic strategies for calcified LM disease.
35	Sareen, 2017	P Cohort (January 2013-December 2015)	Patient with moderate to severely calcified lesions undergoing percutaneous coronary intervention (PCI)	998<br>841 Rota<br>157 Orbital	30 days and 6 months	72%	71<br>Rota : (71.10 ± 10.37)<br>Orbital : (70.96 ± 10.83)	HT<br>Rota : 794 (94.4%)<br>Orbital : 144 (91.7%)<br><br>DM<br>Rota : 414 (49.2%)<br>Orbital : 60 (38.2%)	Rota<br>Asymptomatic= 36 (4.3%)<br>Stable angina= 486 (57.8%)<br>USA= 245 (29.1%)<br>NSTEMI= 73 (8.7%)<br>STEMI= 1 (0.1%)<br><br>Orbital<br>Asymptomatic= 16 (10.2%)<br>Stable angina= 90 (57.3%)	Rotational vs Orbital atherectomy	1. Post procedure : MI, Death, CABG, Dialysis, CHF, CVA, Blood transfusion, CKMB 3-5 or >5<br><br>2. Major vascular complications (death, stroke, CABG, renal failure, or CKMB >10x)<br><br>3. 30-day and 6-month clinical outcomes including	1. Post procedure:<br>MI: 110/841<br>Death: 2/841<br>CABG: 0/841<br>Dialysis: 2/841<br>CHF: 12/841<br>CVA: 0/841<br>TVR: 6/841<br>Blood transfusion: 28/841<br>CKMB 3-5 or >5: 75/841<br><br>2. MACE (death, stroke, renal failure, CKMB >10x): 15/841<br><br>3. 30 days and 6 months clinical	1. Post procedure:<br>MI: 18/157<br>Death: 0/157<br>CABG: 0/157<br>Dialysis: 0/157<br>CHF: 2/157<br>CVA: 0/157<br>TVR: 0/157<br>Blood transfusion: 2/157<br>CKMB 3-5 or >5: 10/157<br><br>2. Major vascular complications: 1/157<br><br>3. 30 days and 6 months clinical outcomes:<br>a. 30 days	no significant differences in 6-month rates of adverse events associated with use of OA vs. RA in patients with moderate to severe calcification undergoing PCI.

No.	First Author, Year	Study design and Period	Study Population	Sample size (n)	Follow-up (months)	Male (n,%)	Age (years)	CAD risk factors n (%)	Clinical pesentation n (%)	Comparisons	Outcomes measured	Events Rota (n/total)	Events Comparator (n/total)	Conclusions
									USA= 43 (27.4%)<br>NSTEMI= 8 (5.1%)<br>STEMI= 0 (0.0%)		death, MI, and TLR, TVR, cerebrovascular acciden (CVA)	outcomes:<br>a. 30 days<br>Death: 0.95%<br>MI : 14.15%<br>TVR : 0.71%<br>TLR : 0.71%<br>CVA: 0.00%<br>TLR, MI, or death: 15.22%<br>TVR, MI, or death: 15.22%<br>Stent thrombosis: 1.19%<br><br>b. 6 months.<br>Death: 2.73%<br>MI : 14.63%<br>TVR : 3.09%<br>TLR : 2.73%<br>CVA: 0.12%<br>TLR, MI, or death: 18.55%<br>TVR, MI, or death: 18.79%<br>Stent thrombosis: 1.43%	Death: 0.00%<br>MI : 11.46%<br>TVR : 0.00%<br>TLR : 0.00%<br>CVA: 0.64%<br>TLR, MI, or death: 11.46%<br>TVR, MI, or death: 11.46%<br>Stent thrombosis: 0.00%<br><br>b. 6 months.<br>Death: 0.00%<br>MI : 11.46%<br>TVR : 0.64%<br>TLR : 0.00%<br>CVA: 0.64%<br>TLR, MI, or death: 11.46%<br>TVR, MI, or death: 12.10%<br>Stent thrombosis: 0.00%	

No.	First Author, Year	Study design and Period	Study Population	Sample size (n)	Follow-up (months)	Male (n,%)	Age (years)	CAD risk factors n (%)	Clinical pesentation n (%)	Comparisons	Outcomes measured	Events Rota (n/total)	Events Comparator (n/total)	Conclusions
36	Tamekiyo, 2009	P Cohort (August 2004 - November 2006)	Patients undergoing PCI with sirolimus-eluting stents (SES) after rotational atherectomy (ROTA) vs. bare-metal stents (BMS)	704 (SES: 79 with ROTA, 625 without ROTA) + historical control 1,123 (BMS: 144 with ROTA, 979 without ROTA)	24 months	SES: 65.8% ROTA, 69.3% non-ROTA BMS: 53.5% ROTA, 70.6% non-ROTA	SES: 70.6±10.7 (ROTA), 69.2±10.2 (non-ROTA) BMS: 70.3±10.4 (ROTA), 68.4±10.7 (non-ROTA)	HT: SES: 76.0% ROTA, 75.2% non-ROTA BMS: 73.6% ROTA, 63.2% non-ROTA DM: SES: 50.6% ROTA, 45.0% non-ROTA BMS: 48.6% ROTA, 41.6% non-ROTA	UA: SES: 31.7% ROTA, 29.9% non-ROTA BMS: 22.2% ROTA, 30.1% non-ROTA	SES vs. BMS, with and without ROTA	MACE, Death, MI, TLR, Stent Thrombosis	**30 days**<br>death: 5 /223<br>CD or MI: 6 /223<br>stent thrombosis: 3 /223<br>TLR: 6 /223<br>MACE:9 /223<br><br>**1 year**<br>death: 24 /223<br>CD or MI: 16 /223<br>stent thrombosis: 6 /223<br>TLR: 64 /223<br>MACE: 72/223<br><br>**2 year**<br>death: 38 /223<br>CD or MI: 21 /223<br>stent thrombosis: 6 /223<br>TLR: 67 /223<br>MACE: 74/223	**30 days**<br>death: 15/1604<br>CD or MI: 22/1604<br>stent thrombosis: 10/1604<br>TLR: 18/1604<br>MACE: 34/1604<br><br>**1 year**<br>death: 66/1604<br>CD or MI: 51/1604<br>stent thrombosis: 13/1604<br>TLR: 251/1604<br>MACE: 291/1604<br><br>**2 year**<br>death: 97/1604<br>CD or MI: 66/1604<br>stent thrombosis: 16/1604<br>TLR: 276/1604<br>MACE: 327/1604	SES after ROTA was associated with lower MACE and TLR compared to BMS after ROTA. However, SES had a limited effect in dialysis patients, even after lesion preparation with ROTA.
37	Tang, 2016	R Cohort (January 2014 - May 2015)	Patients with moderate or severe calcified coronary lesions undergoing RA with CB or plain balloon	132	9 months	58.7% (RACB), 56.5% (RA), 40% (POBA)	RACB: 66 ± 10, RA: 70 ± 7, POBA: 68 ± 9	HT: RACB: 80.4%, RA: 84.8%, POBA: 70% DM: RACB: 34.8%, RA: 37%, POBA: 47.5% Smoking: RACB: 23.9%, RA: 45.7%, POBA: 27.5% Dyslipidemia: RACB:	Unstable angina: RACB: 71.7%, RA: 63.0%, POBA: 80.0% Stable angina: RACB: 28.3%, RA: 37.0%, POBA: 20.0%	rotational artherectomy combined with cutting balloon (RACB) vs RA followed by plain balloon angioplasty (RA group) vs plain old balloon angioplasty only (POBA)	9-month MACE was defined as death, myo-cardial infarction, or target vessel revascularization. Deaths included all causes of mortality.	RACB group (46)<br>Death 1/46<br>MI: 0/46<br>TLR:1/46<br><br>RA group (46)<br>Death:0/46<br>MI:0/46<br>TLR:1/46	POBA group (40)<br>Death: 0/40<br>MI: 0/40<br>TLR: 2/40	Aggressive plaque modification with RA and CB achieve more optimal stent expansion.

No.	First Author, Year	Study design and Period	Study Population	Sample size (n)	Follow-up (months)	Male (n,%)	Age (years)	CAD risk factors n (%)	Clinical pesentation n (%)	Comparisons	Outcomes measured	Events Rota (n/total)	Events Comparator (n/total)	Conclusions
								26.1%, RA: 17.4%, POBA: 20%						
38	Tian, 2015	R Cohort (October 2003-January 2013)	Patients with heavily calcified coronary lesions (HCCL) prior to drug-eluting stent (DES) implantation	737	12 months	NR	RA = 71.9 ± 10.4 years; POBA = 68.0 ± 10.8 years; CBA = 68.7 ± 11.8	HT<br><br>RA = 90.9%; POBA = 80.9%; CBA = 84.2%;	NR	Rotational atherectomy (ROTA)vs. plain old balloon angioplasty (POBA) vs. cutting-balloon angioplasty (CBA)	1-month, 6-month, and 12-month rates of death (all-cause and cardiac), Q-wave MI, TLR, definite stent thrombosis (ST), MACE: defined as the composite of death, Q-wave MI, or TLR.	**RA group (**n=264)<br><br>1 month<br><br>All cause death: 2.8%<br>cardiac death: 0%<br>Q-wave MI: 0%<br>TLR: 0.6%<br>Stent thrombosis (ST) : 0%<br>MACE: 2.8%<br><br>1 year<br>All cause death: 9.8%<br>cardiac death: 3.1%<br>Q-wave MI:0%<br>TLR: 5.2%<br>Stent thrombosis (ST) :0%<br>MACE:14.6%	**POBA group** (n=220)<br><br>1 month<br><br>All cause death: 1.5%<br>cardiac death: 0%<br>Q-wave MI: 0%<br>TLR: 0%<br>ST : 0%<br>MACE: 1.5%<br><br>1-year<br>All cause death: 8.2%<br>cardiac death:2.5%<br>Q-wave MI:0%<br>TLR: 3.5%<br>ST: 0%<br>MACE:12.3%<br><br>**CBA group (**n=253)<br><br>1 month	RA, POBA, or CBA in HCCL may be associated with similar clinical outcomes in patients undergoing percutaneous intervention with DES. The RA group had a trend toward greater MACE, death, and TLR.

No.	First Author, Year	Study design and Period	Study Population	Sample size (n)	Follow-up (months)	Male (n,%)	Age (years)	CAD risk factors n (%)	Clinical pesentation n (%)	Comparisons	Outcomes measured	Events Rota (n/total)	Events Comparator (n/total)	Conclusions
													All cause death: 1.1%<br>cardiac death: 0.5%<br>Q-wave MI: 0%<br>TLR: 0.5%<br>ST : 0.5%<br>MACE: 1.1%<br><br>1 year<br>All cause death: 4.5%<br>cardiac death:1.3%<br>Q-wave MI:0.7%<br>TLR: 3.9%<br>ST: 0.6%<br>MACE: 8.3%	
39	Vaquerizo, 2010	P Cohort (December 2004 - November 2009)	Patients with calcified coronary lesions treated with RA/CB and DES implantation	145 patients	15 ± 11 months	72.4%	68.7 ± 10.1	HT: 73.1%<br>DM: 46.9%<br>Smoking: 26.9%<br>Dyslipidemia: 60.7%	ACS: 42.7%<br>STEMI: 6.2<br>3 vessel disease: 38.6%	CB (cutting balloon) vs RA (rotational atherectomy) alone or followed by CB	MACE were defined as a combined end-point of cardiac death, myocardial infarction (MI), or target lesion revascularization (TLR)	Cummulative FU<br>RA group (n=63)<br>Death: 10 (6.9%)<br>MI: 3 (2.1%)<br>TLR: 5 (3.4%)<br>MACE: 14 (9.6%)<br>ST: 4 (2.4%)	Cummulative FU<br>CB group (n=82)<br>Death: 6 (7.3%)<br>MI: 2 (2.5%)<br>TLR: 2 (2.4%)<br>overall MACE: 8 (9.8%)<br>ST: 2 (2.1%)	Aggressive PM (plaque modification) by CB and/or RA before DES implantation provides excellent mid-term outcomes
40	Wong, 2023	R Cohort (July 2019 - March 2022)	Patients with coronary artery calcification (CAC) undergoing PCI with RA or OA and IVL	25	In hospitalmia 1486	76%	72.2 ± 7.6	HT: 92%<br>DM: 72%<br>smoking: 36%	Stable Ischemic Heart Disease: 76%<br>Acute Coronary Syndrome: 24%	RA and IVL (rotatripsy) vs OA and IVL (orbital-tripsy)	Procedural success and in-hospital MACE (cardiovascular death, MI, and target-vessel revascularization)	MACE: 2/14<br>death (ICH): 1/14<br>death (cardiac arrest): 1/14	MACE: 0/11	Efficacious use of both rotatripsy and orbital- tripsy to modify severe CAC during PCI

No.	First Author, Year	Study design and Period	Study Population	Sample size (n)	Follow-up (months)	Male (n,%)	Age (years)	CAD risk factors n (%)	Clinical pesentation n (%)	Comparisons	Outcomes measured	Events Rota (n/total)	Events Comparator (n/total)	Conclusions
41	Al MacIsaac et al, 1995	P Cohort	Patients with calcified lesions	2161	NR	725 (67%)	66,2 ± 10.3	NR	UA: 475/1063	Non-calcified lesions group	Success and complication rates, angiographic complications	Success: 1016/1078<br>Death: 14/1078	Success: 1031/1083<br>Death: 5/1083	The success rate of rotational atherectomy was not reduced by calcification despite the more frequent complex nature of the calcified lesions.
42	Ayoub et al, 2023	R Cohort (January 2015- December 2019)	patients with calcified coronary artery stenosis	597	NR	78.9%	73.0 ± 9.0	HT: 222 (90.3%), DM: 86 (35.1%),Smoking: 24 (9.8%)	NR	ROTAPRO vs Rotablator	In-hospital major adverse cardiovascular and cerebral event (MACCE) rat	In hospital MACCE: 9/246<br>Mortality: 6/246<br>MI type 4a: 17/246<br>TVR: 7/246<br>Stroke: 0	In hospital MACCE: 20/351,<br>Mortality: 9/351<br>MI type 4a: 3/351<br>TVR: 13/351<br>Stroke: 17/351	The present study demonstrates the safety and efficacy of the new Rotapro system.
43	Desta et al. 2022	R Cohort (January 2005- December 2016)	Patients undergo PCI in heavily calcified lesions using high-speed rotational atherectomy (HSRA)	1479	1 year	1061 (71)	Radial access: 72.3 (9.2); Femoral access: 72.0 (9.3)	HT: 1173 DM: 503 Smoking: 135	NR	Radial access vs femoral access	MACE, including death, myocardial infarction (MI) or target vessel revascularisation (TVR)), in-hospital bleeding and restenosis	**Radial access**<br>MACE: 97 (15.3)<br>All cause mortality: 47 (7.4)<br>TVR: 34 (5.7)<br>Restenosis: 28 (3.1)<br>Target lesion thrombosis: 7 (0.8)	**Femoral access**<br>MACE: 156 (20.2)<br>All cause mortality: 67 (8.6),<br>TVR: 48 (6.6)<br>Restenosis: 52 (5.0),<br>Target lesion thrombosis: 8 (0.8)	HSRA-PCI by TRA was associated with significantly lower risk for in-hospital bleeding and equivalent long-term efficacy when compared with TFA

No.	First Author, Year	Study design and Period	Study Population	Sample size (n)	Follow-up (months)	Male (n,%)	Age (years)	CAD risk factors n (%)	Clinical pesentation n (%)	Comparisons	Outcomes measured	Events Rota (n/total)	Events Comparator (n/total)	Conclusions
44	Dong, 2023	R Cohort (May 2018-July 2021)	individuals with severe coronary artery calcification (CAC) who underwent RA-assisted PCI	318 total patients<br><br>-RA + DES= 261<br>- RA + DCB = 57	1. RA/DCB = 15 months (12.0, 22.5)<br>2. RA/DES = 22 months (15.0, 30.0)	1. RA + DES = 154 (59)<br>2. RA + DCB= 30 (52.63)	1. RA + DES = 65.64±7.99<br>2. RA + DCB= 66.68±8.13	1. RA + DES<br>DM = 96 (36.78%)<br>HT =170 (65.13%)<br>Smoker =78 (29.89%)<br><br>2.RA + DCB<br>DM = 24 (42.11%)<br>HT = 38 (66.67%)<br>Smoker= 14 (24.56%)	NA	RA + DES vs RA + DCB	1. In-hospital events included all-cause mortality, MI, and stroke.<br>2. Long term MACCE (composite of all-cause death, non-fatal MI, TLR, and stroke)	1. In hospital events<br>RA + DES = 7/261<br><br>2. Long term MACCE<br>RA + DES = 49/461	1. In hospital events<br>RA + DCB =0/57<br><br>2. Long term MACCE<br>RA + DCB = 7/57	Drug-coated balloon treatment after rotational atherectomy appears safe and effective in selected severe coronary artery calcification.
45	Gorol, 2018	R Cohort (2009-2016)	patients hospitalized at a high-volume percutaneous coronary intervention (PCI) center who underwent RA	156 total patients<br><br>-Elective RA = 43<br>- Bailout RA (secondary to failed conventional angioplasty) = 113	12 months	1. Elective RA = 27 (62.8)<br>2. Bailout RA = 74 (65.5)	1. Elective RA = 71 ±8.2<br>2. Bailout RA = 69 ± 8.7	1. Elective RA<br>DM = 21 (48.8%)<br>HT =39 (91.2%)<br>Smoker = 11 (26.19%)<br><br>2.Bailout RA<br>DM = 57 (50.4%)<br>HT = 95 (84.1%)<br>Smoker 30 (28.4%)	ACS (UA+NSTEMI)<br>- Elective RA = 14 (32.6)<br>-Bailout RA = 31 (27.4)	Elective RA vs Bailout RA	1. in-hospital major complications (Stroke, MACE, death)<br>2. 1-year complication (death,<br>TLR, TVR, MI, stroke, MACE, death)	**Elective RA (In hospital)**<br>- Stroke =0/43<br>- MACE = 3/43<br>- Death = 1/43<br><br>**Elective RA (1 year)**<br>- Stroke =0/43<br>- MACE = 7/43<br>- Death = 6/43<br>- TLR = 1/43<br>- TVR = 2/43<br>- MI = 2/43	**Bailout RA (In hospital)**<br>- Stroke =1/113<br>- MACE = 6/113<br>- Death = 0/113<br><br>**Bailout RA (1 year)**<br>- Stroke =1/113<br>- MACE = 17/113<br>- Death = 9/113<br>- TLR = 6/113<br>- TVR = 8/113<br>- MI = 8/113	Rotational atherectomy is associated with high efficacy and a relatively low risk of complications, with no significant differences in outcomes between patients treated with primary and secondary RA procedures.

No.	First Author, Year	Study design and Period	Study Population	Sample size (n)	Follow-up (months)	Male (n,%)	Age (years)	CAD risk factors n (%)	Clinical pesentation n (%)	Comparisons	Outcomes measured	Events Rota (n/total)	Events Comparator (n/total)	Conclusions
46	Hemetsberger, 2024	P Cohort (2002-2021)	patients with STEMI and required RA	104 total patients<br>RA-STEMI with shock = 36<br>RA-STEMI Non shock = 68	In hospital	73 (70.2%)	72.8±9.1	DM = 39 (37.5%)<br>HT =93 (89.4%)	All STEMI	RA-STEMI shock vs RA-STEMI non shock	in-hospital death, TLR, TVR, CABG, bleeding BARC type 3 or 5 stroke	RA STEMI shock<br>Death 18/36<br>TLR = 0/36<br>TVR = 0/36<br>CABG =0/36<br>Bleeding = 4/36<br>Stroke = 2/36	RA STEMI Non shock<br>Death 1/68<br>TLR = 2/68<br>TVR = 2/68<br>CABG =1/68<br>Bleeding = 1/68<br>Stroke = 0/68	In this analysis of selected patients, RA was performed as a bailout strategy in the majority, and, as such, RA seems to be feasible with a high procedural success rate.
47	Iwasaki, 2020	R Cohort (January 2016-August 2018)	calcified lesions of nonsmall vessels	157 total patients<br><br>-RA + DES= 88<br>- RA + DCB = 69	1 year	1. RA + DES = 58 (67)<br>2. RA + DCB= 44 (64)	1. RA + DES = 74±8.4<br>2. RA + DCB= 76±7.2	1. RA + DES<br>DM = 41 (47%)<br>HT =51 (68%)<br>Smoking history =56 (64%)<br><br>2.RA + DCB<br>DM = 37 (54%)<br>HT = 54 (79%)<br>Smoking history =41 (60%)	Stable Angina<br>- RA + DES = 86 (98)<br>-RA + DCB = 67 (98)	RA + DES vs RA + DCB	MACE (cardiac death, noncardiac death, target-vessel-related myo cardial infarction, target lesion revascularization (TLR), and major bleeding (BARC ≥ type 3	RA + DES (in hospital)<br>- Cardiac Death = 0/88<br>- Non cardiac death = 0/88<br>- TLR = 0/88<br>- MI = 0/88<br>- Emergency CABG = 0/88<br><br>RA + DES (1 year)<br>- Post-discharge MACE = 7/88<br>- Cardiac Death = 0/88<br>- Non cardiac death = 3/88<br>- TLR = 4/88<br>- Major bleeding = 1/88	RA + DCB (in hospital)<br>- Cardiac Death = 0/69<br>- Non cardiac death = 0/69<br>- TLR = 0/69<br>- NQMI = 0/69<br>- Emergency CABG = 0/69<br><br>RA + DCB (1 year)<br>- Post-discharge MACE = 7/69<br>- Cardiac Death = 0/69<br>- Non cardiac death = 2/69<br>- TLR = 6/69<br>- Major bleeding = 1/69	For calcified lesions of nonsmall vessels, RA + DCB showed good results as well as RA + DES. RA + DCB is a potential new strategy for these lesions

No.	First Author, Year	Study design and Period	Study Population	Sample size (n)	Follow-up (months)	Male (n,%)	Age (years)	CAD risk factors n (%)	Clinical pesentation n (%)	Comparisons	Outcomes measured	Events Rota (n/total)	Events Comparator (n/total)	Conclusions
48	Jung 2023	R Cohort (January 2010 - October 2019)	Patient with severe coronary artery calcification who underwent PCI	540 patient (583 lesions)	1 year	59,8%	non-PMI group: 71.2 ± 10.2<br>PMI group: 73.6 ± 8.5	HT: 76,8%<br>DM: 56,5%<br>Smoking: 19,1%	Stable CAD: 39,2%<br>STEMI and NSTEMI: 60,7%	Rotational atherectomy (non-PMI vs PMI)	MACCE defined as a composite of cardiac death, target-vessel MI, TVR, CVA, total bleeding and repeat revascularization	**Non-PMI**<br>**In Hospital**<br>Death : 8/495<br>Urgent CABG: 2/495<br>Urgent PCI: 5/495<br>Coronary dissect: 38/495<br>Coronary perforation: 7/495<br>total bleeding: 22/495<br>Procedure success: 483/495<br><br>**1 year**<br>MACCE: 49/495<br>death: 20/495<br>MI: 6/495<br>TVR: 28/495<br>CVA: 9/495<br>total bleeding: 20/495<br>repeat revascularization: 32/495	**PMI**<br>**In Hospital**<br>Death : 3/45<br>Urgent CABG: 0/45<br>Urgent PCI: 2/45<br>Coronary dissect: 8/45<br>Coronary perforation: 3/45<br>total bleeding: 5/45<br>Procedure success: 37/45<br><br>**1 year**<br>MACE: 8/45<br>death: 4/45<br>MI: 1/45<br>TVR: 4/45<br>CVA: 0/45<br>total bleeding: 5/45<br>repeat revascularization: 4/45	Peri-procedural myocardial infarction (PMI) after RA in patients with severe CAC was associated with more frequent in-hospital events (p value = 0.005) and a nonsignificant trend for more events during 1 year follow-up
49	Kato, 2012	R Cohort (2009-2010)	Patient with severe calcified de novo lesions who undergoing PCI	21 patients	12 months	47,6%	RA + dual wire balloon group : 76±5<br>RA-only group: 70±13	HT: 90,5%<br>DM: 66,7%<br>Smoking: 57,1%	NR	Rotational atherectomy (RA) + dual wire baloon group vs. RA-only group	Angiographical results at follow up, including diameter stenosis, late loss, in-stent restenosis, target lesion revascularization (TLR) incidence	In stent restenosis: 0/9<br>diameter stenosis: 19 ± 11<br>Late loss: 0.3 ± 0.6<br>TLR: 0/9	In stent restenosis: 2/6<br>diameter stenosis: 32 ± 24<br>Late loss: 0.5 ± 0.4<br>TLR: 1/6	Not statistically significant between combination therapy of RA and dual wire balloon compared to RA only

No.	First Author, Year	Study design and Period	Study Population	Sample size (n)	Follow-up (months)	Male (n,%)	Age (years)	CAD risk factors n (%)	Clinical pesentation n (%)	Comparisons	Outcomes measured	Events Rota (n/total)	Events Comparator (n/total)	Conclusions
50	Kawamoto, 2016	P Cohort (April 2002 - August 2013)	Patients with severely calcified coronary lesions treated with rotational atherectomy (RA)	718 patients (358 planned RA, 309 provisional RA)	12 months	271 (76.1%) in planned RA, 245 (79.3%) in provisional RA	71.1 ± 8.7 (planned RA), 70.6 ± 9.6 (provisional RA)	HT: 300 (83.8%) planned RA, 248 (80.3%) provisional RA DM: 156 (43.6%) planned RA, 131 (42.4%) provisional RA Insulin-dependent DM: 58 (16.2%) planned RA, 33 (10.7%) provisional RA Smoking: 57 (15.9%) planned RA, 75 (24.3%) provisional RA	NSTEMI/UA: 80 (22.3%) planned RA, 95 (30.7%) provisional RA	Planned RA vs. Provisional RA	MACE, Death, MI, TLR, TVR, Stent Thrombosis, Stroke	MACE: 67/358 (22.2%),<br>Death: 17/358 (5.5%),<br>MI: 6/358 (1.9%),<br>TLR: 47/358 (16.8%),<br>TVR: 58/358 (20.9%),<br>Stent Thrombosis: 7/358 (2.1%),<br>Stroke: 2/358 (0.6%)	MACE: 30/309 (13.1%),<br>Death: 10/309 (4.3%),<br>MI: 4/309 (1.9%),<br>TLR: 20/309 (8.9%),<br>TVR: 22/309 (9.8%),<br>Stent Thrombosis: 2/309 (0.8%),<br>Stroke: 2/309 (0.8%)	Planned RA is safe and associated with shorter procedural and fluoroscopy times, reduced contrast volume, and lower pre-dilation balloon use. MACE at 1 year was higher in planned RA group in unadjusted analysis but not significant after propensity adjustment
51	Kotronias, 2019	R Cohort (January 2012 - July 2018)	Patient with severe aortic stenosis undergoing RA PCI	544 patients<br>SAS group : 66<br>no SAS group : 478	In hospital, 30 days, and 1 year	Total : 396 (75%)<br>SAS group : 38 (78%)<br>no SAS group : 358 (75%)	Total : 76 (70,82)<br>SAS group : 82 (79, 87)<br>no SAS group : 76 (69,82)	HT: 90%<br>DM: 30%<br>Smoking: 69%	MI: 26%	Rotational artherectomy : severe aorthic stenosis (SAS) vs no SAS	In-hospital periprocedural incidence and 1 year cumulative incidence of cardiovascular death, periprocedural MI, and stent restenosis	In hospital death: 1/70<br>Death: 9/70<br>Coronary perforation: 0/70<br>Dissect : 1/70<br>Slowflow/no reflow: 0/70<br>Pericardial effusion: 0/70	In hospital death: 0/70<br>Death: 2/70<br>Coronary perforation: 0/70<br>Dissect : 1/70<br>Slowflow/no reflow: 0/70<br>Pericardial effusion: 0/70	RA can be safely performed in patients with severe AS

No.	First Author, Year	Study design and Period	Study Population	Sample size (n)	Follow-up (months)	Male (n,%)	Age (years)	CAD risk factors n (%)	Clinical pesentation n (%)	Comparisons	Outcomes measured	Events Rota (n/total)	Events Comparator (n/total)	Conclusions
52	Ielasi, 2017	R Cohort (April 2002- August 2013)	patients with calcified ULM lesions by RA	962 total patients<br><br>ULM (unprotected left main) =86 patients (86 lesions)<br><br>non-ULM = 876 patients (1060 lesions)	1 year	1. ULM = 60 (69.8%)<br>2. Non-ULM = 688 (78.7%)	1. ULM = 73.2 ±7.5<br>2. Non-ULM = 70.7 ± 9.3	1.ULM<br>DM = 43 (50%)<br>HT =78 (90.7%)<br><br>2.Non ULM<br>DM = 290 (33.1%)<br>HT =704 (80.4%)	NA	ULM vs Non-ULM	1. in-hospital major adverse cardiac events (MACE)<br>2. 1-year MACE (death, MI, and TVR), Definite/probable stent thrombosis (ST) and stroke	ULM (in-hospital)<br>- MACE = 1/86<br>- Death = 1/86<br>- Any MI = 5/86<br>- TLR = 0/86<br>- TVR = 0/86<br>- Stent thrombosis = 1/86<br>- Stroke = 0/86<br><br>ULM (1-year)<br>- MACE = 19/86<br>- Death = 9/86<br>- Any MI = 3/86<br>- TLR = 10/86<br>- TVR = 13/86<br>- Stent thrombosis = 3/86<br>- Stroke = 2/86	Non ULM (in-hospital)<br>- MACE = 70/876<br>- Death = 5/876<br>- Any MI = 62/876<br>- TLR = 3/876<br>- TVR = 6/876<br>- Stent thrombosis = 2/876<br>- Stroke = 3/876<br><br>Non ULM (1-year)<br>- MACE = 99/876<br>- Death = 29/876<br>- Any MI = 6/876<br>- TLR = 68/876<br>- TVR = 81/876<br>- Stent thrombosis = 6/876<br>- Stroke = 3/876	RA followed by stent implantation in patients with heavily calcified ULM narrowing is feasible and associated with good in-hospital results
53	Mangiacapra, 2012	P Cohort (January 2003 - December 2008)	Patients with calcified coronary lesions treated with rotational atherectomy and stenting	187	12 months	71%	73.2 ± 8.9	HT: 70%<br>DM: 35%<br>Smoking: 39%	UA: 23%	ROTA +DES vs. ROTA+BMS	MACE, Stent Thrombosis, TVR	ROTA +DES (n=104)<br>MACE: 12%<br>Stent Thrombosis: 3%<br>TVR: 5%	ROTA+BMS (n=83)<br>MACE: 23.5%<br>Stent Thrombosis: 4.9%<br>TVR: 12.3%	DES associated with significantly lower MACE compared to BMS. Benefit pronounced in smaller stents and longer lesions.

No.	First Author, Year	Study design and Period	Study Population	Sample size (n)	Follow-up (months)	Male (n,%)	Age (years)	CAD risk factors n (%)	Clinical pesentation n (%)	Comparisons	Outcomes measured	Events Rota (n/total)	Events Comparator (n/total)	Conclusions
54	Wu, 2023	R Cohort (February 2017 - January 2022)	Patients with Acute Coronary Syndrome	283	1 month	62.6%	Low speed: 72.06±8 .34 High speed: 72.10 ± 9.58	DM (low: 44.0% high: 28.7%) Smoking: (low: 30.8% high: 28.7%)	UA ((low: 85.2% high: 71.3%) STEMI, (low: 4.9% high: 5.9% NSTEMI: (low: 9.9% high: 22.8%	Low speed vs High speed RA	Heart failure, stent thrombosis, and cardiac death during hospitalization, and 30-day MACCE	**Low Speed (182)** Heart failure, 65/182 Stent thrombosis, 1/182 Cardiac death 3/182 MACCE, 4/182 MI 0/182 Stent thrombosis 0/182 TVR 0/182 Cardiac death, 4/182 Death from any reason, 4/182 Stroke 0/182	**High Speed (101)** Heart failure, 43/101 Stent thrombosis, 1/101 Cardiac death, 4/101 MACCE, 5/101 MI 0/101 Stent thrombosis 0/101 TVR 0/101 Cardiac death, 5/101 Death from any reason, 5/101 Stroke 0/101	patients with ACS treated with a lower RA speed had a higher risk of vasospasm, whereas those treated with higher speeds had a higher incidence of slow flow.
55	Abdel-Wahab, 2012	R Cohort (January 2003 - July 2009)	Patients with heavily calcified de novo coronary lesions treated with rotational atherectomy and DES	205	15 months	73.6%	69.7 ± 9.3	HT: 69.7% DM: 93.1% Smoking: 31.2%	ACS: 16.1% Non ACS: 83.9%	RA only	MACE, Death, MI, TVR, TLR	In hospital Death: 3/205 MI: 5/205 TLR: 0 /205 TVR:1/205 Stroke: 0 /205 MACE: 9/205 long term followup: Death: 17/188 MI: 5/188 TVR: 21/188 TLR: 15/188 MACE: 39/188		RA followed by DES implantation is feasible, safe, and effective with low TLR and MACE at long-term follow-up.
56	Benezet et al, 2011	R Cohort (June 2005- October 2009)	patients with heavily calcified coronary lesions	102	15 months	71 (69.6%)	68.8 ± 7.4	HT: 84 (82.4%) DM:54 (52.9%) Smoking: 47 (46.1%)	UA: 73 (71.6%)	RA only	MACE	Death from all causes: 4/102 Cardiac death: 5/102 AMI: 4/102 Q-wave: 1/102 Without Q-wave: 3/102 TLR: 9/102 Stent thrombosis: 5/102		Death from all causes Cardiac death AMI Q-wave Without Q-wave

No.	First Author, Year	Study design and Period	Study Population	Sample size (n)	Follow-up (months)	Male (n,%)	Age (years)	CAD risk factors n (%)	Clinical pesentation n (%)	Comparisons	Outcomes measured	Events Rota (n/total)	Events Comparator (n/total)	Conclusions
														TLR, Stent thrombosis
57	Bouisset et al, 2021	P Cohort (October 2016- July 2018)	patients with calcified coronary lesions treated by PCI	966	1 year	699(72.4)	74.5±9.8	HT: 792 (82.1) DM: 415 (43.4) Smoking: 162 (19.2)	NR	RA only	Cardiovascular death, myocardial infarction (MI), TLR, stroke and coronary artery bypass grafting (CABG)	**In-hospital outcomes:**<br>Clinical success: 885/963<br>MACE: 45/966<br>Death: 15/965<br>Myocardial infarction: 28/965<br>Stroke or TIA: 3/965<br>Perforation: 16/965<br>Dissection: 38/965<br>Low flow/no flow: 12/965<br>Emergency CABG: 0/965<br>Tamponade: 5/965<br>Bleeding, BARC ≥3: 12/966.<br><br>**1-year follow up:**<br>Clinical success: 127/966<br>MACE: 94/966<br>Death: 55/966<br>Myocardial infarction: 45/966<br>Stroke or TIA: 23/966<br>Perforation: 8/966<br>Dissection: 5/966<br>Low flow/no flow: 33/966<br>Emergency CABG: 29/966		Despite the high level of complexity of the studied population, RA turned out to be an effective procedure with a low rate of in-hospital complications and demonstrated good immediate and midterm results.
58	Chen et al, 2016	R Cohort (January 2009- April 2013)	patients who underwent transradial RA prior to intracoronary stenting for heavily calcified	47	3 years	35/47	63±12.6	HT: 39 (83)<br>DM: 28 (59)<br>Smoking: 31 (65)	NA	RA only	MACE, including death, nonfatal myocardial infarction, and repeat revascularization	Restenosis: 13/47<br>Event free survival: 78%<br>MACE rate: 22%		transradial approach for RA with a small burr is feasible and safe for heavily

No.	First Author, Year	Study design and Period	Study Population	Sample size (n)	Follow-up (months)	Male (n,%)	Age (years)	CAD risk factors n (%)	Clinical pesentation n (%)	Comparisons	Outcomes measured	Events Rota (n/total)	Events Comparator (n/total)	Conclusions
			coronary artery lesions											calcified coronary lesions in most cases, and this procedure is associated with a high success rate and a satisfactory long-term outcome
59	Chiang et al, 2013	R Cohort (March 2004-November 2010)	patients who required RA treatment for severely calcified de novo lesions of native coronary arteries	67	23.2 months (range: 5-86)	42 (62.7%)	73.2 ± 10.3	HT: 58 (86.6)<br>DM: 34 (50.7)<br>Smoking: 33 (49.3)	STEMI: 6 (9.0%), NSTEMI: 13 (19.4%), UA: 24 (35.8%), SA: 24 (35.8%)	RA only	MACE and stent thrombosis	In-hospital MACE (n), %<br>Death: 5 (7.5%)<br>Q wave MI: 0<br>Non-Q wave MI: 1 (1.5%)<br>TLR: 0<br>TVR: 0<br><br>Out-of-hospital MACE (n), %<br>Death: 1 (1.5%)<br>Q wave MI: 0<br>Non-Q wave MI: 0<br>TLR: 7 (10.4%)<br>TVR: 7 (10.4%)<br><br>Stent thrombosis, any (n), %<br>1 (1.5%)		Plaque modification with RA is crucial for successful DES delivery in complex, calcified lesions. It offers high procedural success, low complication rates, and favorable long-term outcomes
60	Chiou, 2020	R Cohort (December 2006 - April 2017)	Patients with severely calcified coronary lesions treated with RA	58	41,2 months	53.4%	74.3 ± 9.7	HT: 91.4%<br>DM: 58.6 %<br>Dyslipidemia: 67.2%<br>Current smokers: 12.1%	Stable angina: 3.4%<br>Unstable angina: 63.8%<br>NSTEMI: 20.7%	All patients receive RA-assisted PCI	Procedural and clinical outcomes (ACS, TLR, TVR, CV death)	41.2 months outcome:<br>- ACS: 6/58<br>- TLR: 12/58<br>- TVR: 2/58<br>- Cardiovascular died: 6/58		This study shows convincing evidence supporting the use of this device to treat complex

No.	First Author, Year	Study design and Period	Study Population	Sample size (n)	Follow-up (months)	Male (n,%)	Age (years)	CAD risk factors n (%)	Clinical pesentation n (%)	Comparisons	Outcomes measured	Events Rota (n/total)	Events Comparator (n/total)	Conclusions
								Ex- smokers: 25.9%	STEMI: 3.4%<br>CHF: 8.6%					calcified coronary lesions using a single-burr strategy.
61	Cho, 2000	R Cohort (April 1997 - April 1999)	Patient with coronary artery stenosis who underwent PCI	30 patients	12.6 ± 8.5 months	20 (66,7%)	62.4 ± 6.3	Systematic HT: 10 (33%)<br>DM: 5 (17%)<br>Smoking: 14 (47%)	UA : 15 (50%)	RA only	Procedural success rate (no death, no coronary perforation, no-reflow, or emergency CABG), side branch occlusion incidence, in hospital complication (death, non-Q MI, and emergency CABG) and follow up (death, nonfatal MI, repeated revascularization)	SBO incidence : 0/30<br><br>In-hospital outcome<br>Procedural success rate : 30/30<br>Death : 0/30<br>Non Q MI : 4/30<br>Emergency bypass surgery : 0/30<br><br>Follow up outcome<br>Death : 0/30<br>Nonfatal MI : 0/30<br>Repeated revascularization : 5/30		
62	Dardas, 2011	R Cohort (January 2002-May 2011)	Patients with CCL who underwent RotA + DES	184	49 months (12-92 months)	130 (70.65)	67 ± 9.2	HT: 149 (80.98%)<br>DM: 49 (26.63%)	NA	RA only	Composite MACE, death, MI, TVR and TLR	MACE: 27/184<br>Death: 7/184<br>Myocardial infarction: 6/184<br>Stroke: 3/184<br>TVR: 6/184<br>TLR: 5/184		The combination of Rota and DES in calcified lesions has a very good angiographic result and a satisfactory

No.	First Author, Year	Study design and Period	Study Population	Sample size (n)	Follow-up (months)	Male (n,%)	Age (years)	CAD risk factors n (%)	Clinical pesentation n (%)	Comparisons	Outcomes measured	Events Rota (n/total)	Events Comparator (n/total)	Conclusions
														clinical outcome.
63	de Melo, 2015	R Cohort (July 2012-November 2014)	all patients undergoing rotational atherectomy with coronary arteries with heavy calcification or previous failed dilation.	29	median: 13.2 months	21 (72.4%)	69.5 ± 7.6	DM: 18 (62.1)<br>HT: 27 (93.1)<br>Smoking: 13 (44.8)	NR	RA only	MACE, defined as death, Q-wave myocardial infarction or repeat target vessel revascularization.	MACE: 3 (10.3)<br>Death from all cause: 1 (3.4)<br>Cardiac death: 0 (0)<br>Q-wave AMI: 0 (0)<br>Target-vessel revascularization: 2 (6.9)		Contemporary rotational atherectomy incorporates less aggressive strategies of ablation with high rates of acute success and low occurrence of major adverse cardiovascular events during late follow-up
64	Dhillon, 2019	R Cohort (January 2008-January 2017)	High-Risk Patients With Severely Calcified Left Main Coronary Artery Disease	55	12 months	64%	73.0 ± 10	HT: 51 (92.7)<br>DM: 33 (60)<br>Smoking: 24 (43.6)	STEMI: 2 (3.6%), NSTEMI: 27 (49.1%), UA: 15 (27.3%), SA: 11 (20.0%)	RA only	In-hospital MACCE	Cardiac mortality: 2/55<br>Major bleeding: 4/55<br>AKI: 7/55<br>Stroke: 0<br>Perforation: 0<br>dissection: 0		RA of the LM coronary artery can be performed safely and is associated with a high rate of angiographic success.

No.	First Author, Year	Study design and Period	Study Population	Sample size (n)	Follow-up (months)	Male (n,%)	Age (years)	CAD risk factors n (%)	Clinical pesentation n (%)	Comparisons	Outcomes measured	Events Rota (n/total)	Events Comparator (n/total)	Conclusions
65	Dong, 2021	R Cohort (January 2013-December 2015)	Percutaneous coronary intervention (PCI) in hemodialysis patients with severely calcified and diffused lesions	138	12 months	38 (27.5%)	68 (54–78)	HT: 126<br>DM:73<br>Smoking: 17	NR	RA only	MACE	Cummulative rates of MACE: (61/138)<br>umulative rates of TLR: 21.01% (29/138)<br>Cumulative all-cause death at 30-day follow-up: 6.52%<br>Cumulative all-cause death at 1-year follow-up 18.8%		the overall prognosis of ROTA-facilitated PCI in hemodialysis patients was poor. ISR was a significant risk factor for MACE, especially TLR
66	Dong, 2020	R Cohort (January 2013 - November 2015)	Patients with de novo calcified lesions and in stent restenosis who undergo PCI	1169 patients	24 months	762 (65.2%)	75 (69–81)	HT: 1018 (87.1%)<br>DM: 521 (44.6%)<br>Smoking: 136 (11.6%)	STEMI : 1 (0.1%)<br>NSTEMI : 1 (0.1%)<br>UA : 35 (3.0%)<br>Stable CAD : 1132 (96.8%)	Modified RA, including RA+DES, DCB, or POBA	Early MACE, defined as combination of all-cause death and TLR during hospitalization and 30 days after PCI<br><br>Midterm MACE, defined as 12 and 24-month rates of all-cause death, cardiac death, first occurrence of hospitalization due to heart failure, definite stent thrombosis, and TLR	Early MACE<br>In-hospital death : 29/1169<br>30-day death : 6/1169<br>TLR : 0/1169<br><br>12-month MACE<br>MACE : 240/1169<br>TLR : 120/1169<br>All-cause death : 64/1169<br>Cardiac death : 28/1169<br>HF : 27/1169<br>Stent thrombosis : 1/1169<br><br>24-month MACE<br>MACE : 313/1169<br>TLR : 137/1169<br>All-cause death : 70/1169<br>Cardiac death : 36/1169<br>HF : 69/1169<br>Stent thrombosis : 1/1169		Modified RA technique is a safe and effective, even in high-risk patients

No.	First Author, Year	Study design and Period	Study Population	Sample size (n)	Follow-up (months)	Male (n,%)	Age (years)	CAD risk factors n (%)	Clinical pesentation n (%)	Comparisons	Outcomes measured	Events Rota (n/total)	Events Comparator (n/total)	Conclusions
67	Eftychiou et al, 2016	R Cohort (March 2005-January 2013)	Patients undergoing RA for calcified coronary lesions	518	22 (6-85) months	347 (68.3%)	72 (37-94)	HT: 382 (73.9%) DM: 148 (28.7%) Smoking: 283 (55.7%)	ACS: 178 (34.6%)	Rotational atherectomy (single arm)	MACE, Death, MI, TVR, Stent Thrombosis, Stroke	MACE: 92/518 (17.8%), Death: 71/518 (13.7%), MI: 60/518 (11.7%), TVR: 39/518 (7.5%), Stent Thrombosis: 7/518 (1.4%), Stroke: 15/518 (2.9%)		RA was associated with high procedural success and acceptable MACE rates. Higher SYNTAX score and ACS were predictors of worse outcomes
68	Ferri, 2016	R Cohort	Petient for the treatment of undilatable underexpanded stants implanted in coronary calcific lesions	16 patients	In hospital and 1 year	50%	65 ± 13.8	HT: 87,5% DM: 37,5% Smoking: 12,5%	UA: 12,5% Stable angina: 87,5%	Rotational atherectomy (single arm)	In hospital periprocedural incidence and 1 year cumulative incidence of cardiovascular death, periprocedural MI, and stent restenosis	**In hospital**<br>Death: 0/16<br>CKMB elevation: 1/16 (6.3%)<br>Coronary dissect: 1/16 (6.3%)<br>Coronary perforation: 0/16<br>Burr entrapment: 1/16<br>Complication: 2/16<br><br>**1 Year**<br>Death: 1/15<br>MI : 1/15<br>TLR : 2/15<br>Stroke : 0/15<br>MACE: 4/15		RA was an effective treatment for resistant stent underexpansion with acceptable outcomes
69	Furuichi, 2009	R Cohort (April 2002-November 2006)	patients with severely calcified de novo lesions undergoing RA before DES implantation	95 patients (96 lesions)	14.7 (6.0–57.7)	82 (86.3%)	68 ± 9	HT: 72 (75.8%), DM: 29 (30.5%), Smoking: 40 (42.1%)	UA: 19 (20%), Prior MI: 41 (43.2%)	Rotational atherectomy (single arm)	MACE, Death, MI, TLR, TVR, Stent thrombosis	MACE: 15/95 (15.8%), Death: 4/95 (4.2%), Q-wave MI: 2/95 (2.1%), Non-Q-wave MI: 3/95 (3.2%), TLR: 9/95 (9.5%), TVR: 11/95 (11.6%), Stent Thrombosis: 4/95 (4.2%)		RA followed by DES implantation in severely calcified coronary lesions is feasible with high procedural

No.	First Author, Year	Study design and Period	Study Population	Sample size (n)	Follow-up (months)	Male (n,%)	Age (years)	CAD risk factors n (%)	Clinical pesentation n (%)	Comparisons	Outcomes measured	Events Rota (n/total)	Events Comparator (n/total)	Conclusions
														success and acceptable TLR rate
70	Garcia-Lara, 2011	R Cohort (June 2005 - June 2008)	Patient with severely calcified ULM stenosis and not eligible for CABG	40 patients	24 months	26 (65%)	73 ± 8.3	HT: 36 (90%)<br>DM: 22 (55%)<br>Smoking: 8 (21%)	NSTEMI : 7 (17.5%)<br>UA : 16 (49%)<br>Stable CAD : 3 (7.5%)	Rotational atherectomy (single arm)	In hospital outcome, including procedural and 24-hour death, major and minor bleeding, and elevated CK-MB. 2 year follow up, including death, cardiac mortality-free survival rate, and TVR rate	In Hospital<br>Procedural death : 1/40<br>24 hours death : 2/40<br>Major bleeding : 1/40<br>Minor bleeding : 2/40<br>Elevated CK-MB : 9/40<br><br>2 year follow up<br>Death : 13/40<br>Cardiac mortality-free survival rate : 71 ±7%<br>TVR rate : 19.3 ± 7%		RA on severely calcified left main stenosis is feasible and could pose the only possible effective treatment
71	Gioia, 2000	P Cohort (January - July 1999)	Patients with coronary lesions	16 total patients: 19 total of treated vessels	In hospital	13 (81%)	62 ±12	DM = 6 (38%)<br>HT = 8 (50%)<br>Smoking = 4 (25%)	UA = 8 (50%)<br>Stable angina = 5 (31%)	RA without Stent vs RA + Stent	1. Vascular complication<br>2. Non-Q myocardial infarction	1. Vascular complication = 0/16<br>2. Non-Q myocardial infarction = 2/16		Transradial PTCRA when used in conjunction with IVUS of the RA is a safe and feasible procedure in selected cases.

No.	First Author, Year	Study design and Period	Study Population	Sample size (n)	Follow-up (months)	Male (n,%)	Age (years)	CAD risk factors n (%)	Clinical pesentation n (%)	Comparisons	Outcomes measured	Events Rota (n/total)	Events Comparator (n/total)	Conclusions
72	Hernandez, 2018	P Cohort (January 2013 and July 2017)	patients requiring stent ablation	11 total patients	5-32 months	10 (90.9)	65.4±18 .6	DM = 4 (36.4%)<br>HT =8 (72.7%)	AMI = 5<br>UA = 1<br>Stable angina = 5	RA only	MACE (composite of death, myocardial infarction, and target vessel revascu-larization (TVR) events)	In hospital<br>1. In hospital MACE = 0/11<br>2. In hospital death = 0/11<br><br>Long Term follow up<br>1. MACE = 1/11<br>2. MI = 1/11<br>TLR = 0/11<br>Non-TLR = 2/11<br>Death = 0/11		RA of under expanded stents is a feasible option with a high rate ofprocedural success. At long-term follow-up, all of them were alive and 90.9% ofpatients remained free from MACE
73	Jariwala, 2023	R Cohort (January 2015 - June 2022)	Patient with complex and calcified coronary lesion who undergo PCI	376 patients	12 months	241 (64%)	68.5 ± 26.4	HT: 288 (76,5%)	NR	Rotational atherectomy (single arm)	Periprocedural outcome and long-term MACE, included CV and all-cause death, MI, and target vessel revascularization	Coronary dissection : 12/376<br>Burr entrapment : 10/376<br>MACE : 31/376<br>All-cause mortality : 21/376<br>TVR : 17/376		
74	Jiang, 2012	R cohort (August 2006 - August 2012)	Patients with complex coronary lesions who underwent RA followed by DES	253 patients	mean follow up 3 years after discharge	69%	73 ± 12	HT = 66%<br>DM = 25%<br>Smoking = 30%	Angina = 89%	NA	Death, acute MI, target lesion restenosis, new coronary lesions, and target lesion revascularization.	In hospital<br>Death = 2/253<br>AMI = 3/253<br><br>Follow up (mean 3 years)<br>Death = 1/251<br>AMI = 2/251<br>Restenosis = 14/251<br>TLR = 8/251		RA- DES is a safe and effective technique for patients with complex coronary lesions, especially calcified and non-dilatable lesions.

No.	First Author, Year	Study design and Period	Study Population	Sample size (n)	Follow-up (months)	Male (n,%)	Age (years)	CAD risk factors n (%)	Clinical pesentation n (%)	Comparisons	Outcomes measured	Events Rota (n/total)	Events Comparator (n/total)	Conclusions
75	Jossart, 2021	R Cohort (June 2015 - December 2019)	Patient with severe calcified lesion who have undergone PCI	100 patients	6 months	74%	72.1 ± 8.6	HT: 85%<br>DM: 39%<br>Smoking: 17%	NR	Rotational atherectomy followed by drug-eluting stent implantation (single arm)	In-hospital and 6-month cumulative incidence of cardiovascular death, periprocedural MI, stent restenosis	**In hospital**<br>No-reflow : 1/100<br>Perforation and burr entrapment : 0/100<br><br>**6 months follow-up**<br>Cardiovascular Death: 1/100<br>Periprocedural MI: 1/100<br>Stent restenosis: 1/100		RA results in a high success rate in terms of procedural performance and clinical outcomes
76	Jujo K , 2017	R Cohort (2004 - 2013)	Patient with severely calcified coronary artery who underwent PCI	315 patients	3 years	81%	NR	NR	NR	Rotational atherectomy (single arm)	Cardiovascular death	cardiovascular death: 72/315 (cumulative risk, 23%), 35 patient were within the first year		RA results in a high success rate for procedural performance and clinical outcomes.
77	Jujo K, 2019	R Cohort (2004 - 2015)	Patients with de novo calcified lesion after PCI	1089 patients	5 years	245/302 (81%)	Total : 65.0 ± 10.1	HT: 87%<br>DM: 66%<br>Smoking: 23%	MI: 25%	Rotational atherectomy followed by drug-eluting stent implantation (HD vs non HD group)	Death	death non-HD: 59/302 (19.5%)<br>death HD: 59/787 (7.5%)		The incidence of death in HD group is significantly lower compared non HD group
78	Kauffman, 1989	P Cohort (October 1988 - January 1989)	Patients undergoing coronary atherectomy for coronary artery stenosis	50 patients (53 procedure)	In-hospital follow-up	76%	61	NR	CAD unspecified.<br>NYHA A: 17<br>NYHA III: 15<br>NYHA II: 15<br>NYHA I: 3<br>1 vessel: 21<br>2 vessel: 12<br>3 vessel: 17	No comparison, RA only	Reduction of the stenosis by 40%, infarction, and no need for a bypass surgical procedure.	reduction of the stenosis atleast by 40%: 47/53<br>non Q-wave MI: 3/53<br>Bypass procedure: 5/53		Coronary atherectomy promises to be a relatively safe and efficient treatment of coronary artery stenosis.

No.	First Author, Year	Study design and Period	Study Population	Sample size (n)	Follow-up (months)	Male (n,%)	Age (years)	CAD risk factors n (%)	Clinical pesentation n (%)	Comparison s	Outcomes measured	Events Rota (n/total)	Events Comparator (n/total)	Conclusions
79	Kawamoto (ROTATE study), 2016	R Cohort (2002 - 2013)	De novo lesions with calcified coronary lesions	985 patients (1176 lesions)	2 year	78,30%	70.9 ± 9.2	HT: 80,9%<br>DM: 34,5%<br>Smoking: 47,3%	STEMI : 26,2%	No comparison, RA only	Procedural complication, defined as dissection, perforation, slow/noflow, VF, and wire break<br><br>MACE defined as composite periprocedural MI, TVR, and death	**Procedural complication (n = total lesions)**<br>dissection : 82/1176<br>perforation: 12/1176<br>slow/noflow : 13/1176<br>VF: 1/1176<br>Wire break: 1/1176<br><br>**In hospital**<br>MACE: 82/985<br>Death: 6/985<br>PMI: 73/985<br>Stroke: 3/985<br>TLR: 4/985<br>TVR: 7/98<br><br>**1 year**<br>MACE: 121/985<br>Death: 38/985<br>Follow up MI: 13/985<br>Stroke: 6/985<br>TLR: 82/985<br>TVR: 97/985<br><br>**2 year**<br>MACE: 164/985<br>Death: 59/985<br>Follow up MI: 20/985<br>Stroke: 7/985<br>TLR: 106/985<br>TVR: 126/985		RA appears to be safe and effective with acceptable in-hospital and follow-up MACE considering

No.	First Author, Year	Study design and Period	Study Population	Sample size (n)	Follow-up (months)	Male (n,%)	Age (years)	CAD risk factors n (%)	Clinical pesentation n (%)	Comparisons	Outcomes measured	Events Rota (n/total)	Events Comparator (n/total)	Conclusions
80	Khattab, 2007	P Cohort (January-December 2004)	Patients with CAD and subjected to PCI	61	9 months and 2 year	DES group = 17 (63%)<br>BMS group = 30 (88%)	DES group = 70 ± 8<br>BMS group = 71 ± 10	DES group<br>DM = 7 (26%)<br>HT = 26 (96%)<br><br>BMS group<br>DM = 14 (41%)<br>HT =30 (88%)	UA<br>DES = 4 (15%)<br>BMS = 7 (21%)	All patients were treated by rotablation followed by Drug-eluting stents (DES) vs Bare metal stents (BMS)	**9 months**<br>MACE (i.e. postprocedural MI, and/or an elevation of CK-MB to greater than twice normal; death, TLR)<br><br>**2-year FU**<br>death and MI	9 month<br>- DES (n=27)<br>Death, MI, or TLR : 2/27<br>Death any cause : 0/27<br>MI : 0/27<br>Clinical TLR : 2/27<br><br>- BMS (n = 34)<br>Death, MI, or TLR : 13/34<br>Death any cause : 1/34<br>MI : 1/34<br>Clinical TLR : 12/34	At 2-year<br>- DES<br>1 sudden death<br>1 subdural hematoma<br>1 major stroke<br>1 aortic valve endocarditis<br>1 lung cancer<br><br>- BMS<br>3 death (unknown)<br>1 renal failure	Rota-DES has a favorable effect on clinical and angiographic outcomes at 9 months when treating heavily calcified lesions compared to rota-BMS.
81	Koch, 2002	P Cohort	Patient with de novo coronary artery calcified lesions	25 patients	In hospital	70%	61 ± 9	NR	UA: 80%	No comparison, RA only	The incidence, extent, and severity of perfusion defects induced by RA	Transient perfusion defects : 23/25 (92%)<br>Perfusion in 3.3 ± 2.5 region		
82	Kubota, 2010	R Cohort (January 2003 - March 2010)	Patients with CAD who underwent PCI	251	6-12 months for BMS<br>10-18 months for SES	BMS = 67%<br>SES = 63,4%	BMS = 71.5 ± 9,45<br>SES = 71.8 ± 8.10	DM<br>BMS = 46.5%<br>SES = 46.9%	NA	BMS (n=84) vs SES (n=167) using RA	primary: cardiac death, nonfatal recurrent MI<br><br>Secondary: in-stent binary restenosis (BR, defined as >50% diameter stenosis)	Primary endpoints :<br>SES = 2.3%<br>BMS = 7.1%<br><br>Secondary endpoints<br>SES = 21.3%<br>BMS = 27.1%		Posiitive long-term safety of SES for calcified lesions using a rotablator in daily practice, SES did not show a benefit for the angiographic outcomes compared to BMS.

No.	First Author, Year	Study design and Period	Study Population	Sample size (n)	Follow-up (months)	Male (n,%)	Age (years)	CAD risk factors n (%)	Clinical pesentation n (%)	Comparisons	Outcomes measured	Events Rota (n/total)	Events Comparator (n/total)	Conclusions
83	Li, 2013	R Cohort (March 1, 2010 - September 1, 2012)	Patient with heavily calcified coronary lesions who underwent PCI	65 patients (78 lesions)	17.6 ± 8.5 months	NR	NR	NR	NR	No comparison, RA only	In-hospital periprocedural outcome (success rate) and follow up MACE	success rate : 78/78<br>average burr/artery ratio : 0.50 ± 0.04<br>average number of burr/case : 1.15 ± 0.36<br>follow up MACE : 9/65		RA-DES is a safe and efficient technique for treating heavily calcified coronary lesions
84	Lippmann, 2017	R Cohort	Patient with severe aortic stenosis with calcific coronary artery disease	29 patients	NR	55,2%	79.8 ± 8.8	HT: 93,1%<br>DM: 24,1%<br>Smoking: 41,3%	MI: 34,5%	No comparison, RA only	MACCE defined as a composite of cardiac death, target-vessel MI, and CVA	death : 0/29<br>stroke : 0/29<br>coronary perfortion :0/29<br>transient no reflow :1/29<br>coronary dissect :2/29<br>MI : 0/29		RA-facilitated PCI can be safely performed in patients with severe AS and severely calcified CAD with low risk of complications
85	Lunardi, 2020	R Cohort	Patients with CAD and calcified coronary lesions undergoing trans-catheter aortic valve implantation (TAVI)	19 patients	24 month	57,89%	81.4±7.2	HT: 84,2%<br>DM: 42,1%	NR	Rotational atherectomy with TAVI (single arm)	In hospital death, troponin rise, hospital stay and long term follow-up (death, major stroke, TLF, urgent revascularization, cumulative MACE	**In hospital**<br>death : 1/19<br>Troponin rise: 19/19<br>hospital stays: 9.9 ± 10.5<br><br>24 **months**<br>Death: 6/18<br>major stroke : 0/18<br>TLF : 1/18<br>MACE : 3/18<br>Urgent revascularization : 0/18		RA concomitant with TAVI was feasible and safe in patients

No.	First Author, Year	Study design and Period	Study Population	Sample size (n)	Follow-up (months)	Male (n,%)	Age (years)	CAD risk factors n (%)	Clinical pesentation n (%)	Comparisons	Outcomes measured	Events Rota (n/total)	Events Comparator (n/total)	Conclusions
86	Malik, 2021	R Cohort (November 2014 - October 2019)	CKD patient with calcified coronary artery disease who underwent PCI	203 patients	24.11 ± 18.05 months	149 (73.4)	63.94 ± 8.79	HT: 184 (90.6%)<br>DM: 133 (65.5%)<br>Smoking: 104 (51.2%)	ACS : 54 (27%)<br>Stable CAD : 149 (73%)	Rotational atherectomy followed by second or third-generation DES implantation (single arm)	procedural success (achieving angiographic success without in-hospital MACE), follow up MACCE, and in-stent restenosis	Procedural success : 198/203<br><br>In hospital complication<br>MI : 3/203<br>Death : 3/203<br>Cardiac arrest : 4/203<br>Stent thrombosis : 3/203<br>Stroke : 1/203<br><br>Follow up MACCE<br>Death : 18/203<br>MACCE : 22/203<br>In stent restenosis : 1/203		RA followed by second-or third-generation DES implantation is feasible and safe with high procedural success and low in-stent restenosis
87	Mankerious, 2020	R Cohort (June 2010 - December 2018)	Patient with calcified lesions who underwent PCI	323 patients	1 year	72%	74 ± 8	HT: 88,85<br>DM: 35,6%<br>Smoking: 25,7%	UA: 6,81%<br>MI: 13,62%	Rotational atherectomy (single arm)	In-hospital adverse outcomes (i.e. persistent slow flow, dissection, perforation, burr entrapmen, and MACE).<br><br>1 year cumulative incidence of MACE, mortality, and TVR	**In hospital**<br>persistent slow flow : 13/323<br>dissection : 19/323<br>perforation : 8/323<br>burr entrapment : 2/323<br>in-hospital MACE : 14/323<br>**1 year**<br>MACE : 66/232<br>death : 19/232<br>TVR : 31/232		Rotational coronary atherectomy is an effective treatment in patient with coronary ostial lesions
88	Mezilis, 2010	R Cohort (2002-2008)	Patients with CAD who underwent RA	150	Mean follow up 3 years, max 78 months	111 (74%)	70 ± 8	HT = 123 (82%)<br>DM = 33 (22%)	Stable angina = 138 (92%)<br>Acute MI = 3 (2%)	NA	MACE : the occurrence of death, MI, stroke, TLR, and TVR.	94% (141/150) patients were followed up<br><br>Death : 4 (1 non cardiac)<br>MI : 5<br>Stroke : 2<br>TVR : 3<br>TLR : 3<br>MACE : 17		RA-DES has a favorable effect for heavily calcified lesions in both the angiographic and clinical

No.	First Author, Year	Study design and Period	Study Population	Sample size (n)	Follow-up (months)	Male (n,%)	Age (years)	CAD risk factors n (%)	Clinical pesentation n (%)	Comparisons	Outcomes measured	Events Rota (n/total)	Events Comparator (n/total)	Conclusions
														outcomes. No safety concerns are observed up to 6 years.
89	Motwani, 2000	P Cohort (July 1992 - June 1997)	All patients who underwent rotational atherectomy of atherosclerotic RCA ostial stenosis	119	6 months after intervention.	58 (49%)	69 ±9	DM = 15 (12.6%) HT =72 (60.5%) Smoking = 22 (18.5%)	UA = 53 (44.5%)	RA only	1. Death 2. Restenosis	1. Death = 3/119 (2 non cardiac (1 COPD, 1 malignancy), 1 CHF) 2. Restenosis 18/119		RA of right coro nary ostial stenosis results in excellent acute procedural success and in low incidence of clinical recurrence.
90	Naito, 2012	R Cohort (September 2004- April 2010)	Patients with DES implanta-tion following rotational atherectomy for calcified coronary stenosis	233	21 months	74.3 % (SES), 79.6 % (PES)	69.0 ± 8.8 (SES), 67.3 ± 9.3 (PES)	HT:72.6% (SES), 77.7% (PES), DM: 50.3% (SES), 42.6% (PES) Smoking: 17.3% (SES), 24.1% (PES)	Acute MI: 3.4% (SES), 9.3 (PES) UA: 7.3% (SES), 7.4 % (PES) ACS: 10.6% (SES), 16.7% (PES)	Sirolimus-eluting stents (SES) vs. Paclitaxel-eluting stents (PES)	TLR, TVR, MACE	SES TLR: 8/162 TVR: 11/162 MACE: 24/162 PES TLR: 5/51 TVR: 6/51 MACE:7/51		No significant difference in long-term clinical outcomes between SES and PES, both following rotational atherectomy.

No.	First Author, Year	Study design and Period	Study Population	Sample size (n)	Follow-up (months)	Male (n,%)	Age (years)	CAD risk factors n (%)	Clinical pesentation n (%)	Comparisons	Outcomes measured	Events Rota (n/total)	Events Comparator (n/total)	Conclusions
91	Patel, 1997	P Cohort (October 1995 - January 1997)	cardiac transplant patients who developed de novo coronary artery disease	18	Maximal 64 months	88%	51.3 +- 3.1 (45 – 63	HT: 100%<br>DM: 27.8%<br>Dyslipidemia: 44.4%	Angina: 11%<br>MI: 22%	PTCA (10 patients) vs **PTCRA (6)** vs CABG (5) vs TMLR (1).	Restenosis, death, EF improval	PTCRA was successful in all patients with a decrease in mean stenosis from 83.4 +-4.4 to 11.7+-1.9% reveal, no reocclusion after follow-up<br><br>Two PTCA patients require restenosis. Two CABG patients who had hypertrophy died of heart failure 2 and 9 days after their operations. One CABG died after 15 days because of pulmonary failure. TMLR patient show clinical improvement.		Revascularization procedures can be effective in patients with CAD after cardiac transplantation and that coronary angioplasty or atherectomy would be a therapy of choice for single proximal lesions
92	Popma, 1993	R Cohort (April 1990 - June 1992)	Patient with corinary ostial lesion	105 patients	6 months	63,86%	66 (37 - 89)	NR	UA: 50%<br>MI: 38%	Rotational atherectomy (single arm)	Incidence of death, coronary spasm, dissection, post-procedural coronary trombus	Death: 4/105<br>Coronary spasm: 3/105<br>Dissect: 18/105<br>Post-procedural coronary trombus : 3/105		Rotational coronary atherectomy is an effective alternative treatment to balloon angioplasty in patient with coronary ostial lesions
93	Rathore, 2010	R Cohort (January 2003 and December 2007)	patients with fibro-calcified coronary lesion treated with rotational atherectomy	516	6-9 months	65,10%	70.60 ± 8.79	HT: 66.9%<br>DM: 40.1%<br>Smoking: 40.1%	Acute MI: 1.2%<br>UA: 13.0%	ROTA+Drug-eluting stents (DES) vs. ROTA+ Bare-metal stents (BMS)	Binary restenosis, target lesion revascularization (TLR), major adverse cardiac events (MACE)	ROTA+ DES<br>Binary restenosis: 29/391<br>TLR: 28/391<br>MACE: 10/391<br>ROTA+ BMS<br>Binary restenosis: 27/125		DES significantly reduces binary restenosis and TLR compared to

No.	First Author, Year	Study design and Period	Study Population	Sample size (n)	Follow-up (months)	Male (n,%)	Age (years)	CAD risk factors n (%)	Clinical pesentation n (%)	Comparisons	Outcomes measured	Events Rota (n/total)	Events Comparator (n/total)	Conclusions
			followed by stenting									TLR: 24/125<br>MACE: 5/125		BMS, with high procedural success and low complications.
94	Rissanen, 2017	R Cohort (March 2011- December 2013)	Patients with calcifed de novo coronary lesions treated with PCI using drug-coated balloon (DCB) after rotational atherectomy (rotablation)	65	24 months	68%	72 ±10	HT: 75%<br>DM: 37%<br>smoker: 12%<br>Ex-smoker: 26%<br>Dyslipidemia: 89%	ACS: 32%	All patients received RA followed with PCI	Composite MACE, death, MI, and TLR	12 Months (only presented in percent)<br>Overall MACE rate: 14%<br>CV Death: 6.2%<br>Non fatal MI:6.2%<br>TLR Rate : 1.5%<br><br>24 Months (only presented in percent)<br>Overall MACE rate: 20%<br>CV Death: 12.3%<br>Non fatal MI:6.2%<br>TLR Rate: 3.1%		PCI using DCB after preparation of calcified lesions with rotablation is safe and effective, in complex calcified lessions with a bleeding risk
95	Sakakura, 2016	P Cohort (January 2013 - July 2016)	Patients undergoing rotational atherectomy for coronary artery disease (J-PCI Registry)	13,355	In hospital	69.8%	72.6±9.6	HT: 79.4%<br>DM: 53.4%<br>Smoking: 23.5%	N/R	RA only	In-hospital death, cardiac tamponade, emergent surgery	In-hospital death: 80/13335<br>cardiac tamponade: 86/13335<br>emergent surgery: 24/13335<br>Composite outcomes: 175/13335		Complication risks linked to patient condition and institutional volume. High-volume centers had fewer complications.
96	Sharma, 1998	P Cohort (November 1995 - November 1996)	Patients with in-stent restenosis treated with RA	100 patients	All patients have completed at least 9 months of follow-up, with a mean of 13 +- 5 months.	72%	62 ± 12	Two or more conventional risk factors (hypertension, hypercholesterolemia, diabetes mellitus, smoking and	NR	No comparison, All patients receive RA-assisted PCI	In-hospital outcome<br>Minor angiographic dissections, slow flow, CKMB enzyme elevation<br><br>Late (9-months)	In hospital outcome<br>- Minor angiographic dissections: 7/100<br>- Slow flow: 3/100<br>- CKMB enzyme elevation: 11/100<br><br>Late (9-months) clinical result<br>- Uncomplicated non–Q-wave MI: 5/100<br>- Repeat in-stent restenosis: 28/100<br>- Total Vessel Restenosis: 28/100		Rotational atherectomy is a safe and feasible technique for treatment of ISR and is associated with a relatively low

No.	First Author, Year	Study design and Period	Study Population	Sample size (n)	Follow-up (months)	Male (n,%)	Age (years)	CAD risk factors n (%)	Clinical pesentation n (%)	Comparisons	Outcomes measured	Events Rota (n/total)	Events Comparator (n/total)	Conclusions
								family history) were present in 53% of cases.			clinical results: uncomplicated non–Q-wave MI, repeat in-stent restenosis:, total Vessel Restenosis.			recurrent restenosis
97	Simsek, 2022	P Cohort (2012-2022)	Patients with CTO (chronic total occlusion) PCI (devided by balloon undilatable and balloon dilatable lesions)	6535	In-hospital follow-up	80%	64 ± 10	HT: 89%<br>DM: 42%<br>Smoking: 26%<br>Dyslipidemia: 83%	NR	Balloon dilatable vs balloon undilatable (A cutting balloon : 27%, a scoring balloon: 15%, laser:14%, **RA: 28%**, OA: 10%, IVL: 1% and other modalities: 5%	Procedural success and MACE (composite of in-hospital death, acute myocardial infarction, stroke, re-PCI, emergency CABG, and pericardiocentesis)	Balloon dilatable (558)<br>all-cause death: 7/558<br>AMI:6/558<br>stroke:1/558<br>re-PCI:3/558<br>emergency CABG: 2/558<br>pericardiocentesis: 17/558<br><br>Balloon undilatable (5977)<br>all-cause death, 17/5977<br>AMI: 23/5977<br>stroke:7/5977<br>re-PCI:11/5977<br>emergency CABG:0/5977<br>pericardiocentesis:35/5977		Approximately 1 in 12 CTO (8.5%) lesions are balloon undilatable. Treatment of balloon undilatable lesions is associated with lower technical success and higher in-hospital MACE.

No.	First Author, Year	Study design and Period	Study Population	Sample size (n)	Follow-up (months)	Male (n,%)	Age (years)	CAD risk factors n (%)	Clinical pesentation n (%)	Comparisons	Outcomes measured	Events Rota (n/total)	Events Comparator (n/total)	Conclusions
98	Takagi, 2022	R Cohort (April 1993 - June 2001)	Patients with unprotected left main coronary artery stenosis undergoing PCI	67	31 ± 23 months	84%	65 ± 12	HT: 55%<br>DM: 9%<br>Smoking: 46%<br>Dyslipidemia 70%	Unstable angina: 40%	Stents only (39)<br>**RA + stents** (12)<br>directional coronary atherectomy + stents (13)<br>directional coronary atherectomy only (3)	MACE (death, Q-wave or non–Q-wave myocardial infarction (MI), or coronary artery bypass graft (CABG))	**In-hospital outcome**<br>Death: 0<br>CABG: 2/67<br>MI: 5/75<br>Q-wave MI:2/67<br>Non–Q-wave MI : 3/67<br>Total MACE: 6/67<br><br>**Follow-up outcome**<br>Death: 11/67<br>Cardiac death: 8/67<br>CABG: 5/67<br>MI: 2/67<br>TVR: 16/67<br>Total MACE at follow-up: 23/67		Elective percutaneous coronary intervention of de novo lesions in left main coronary arteries is feasible, with low procedural risk.
99	Tan, 2021	R Cohort (September 2017 - June 2018)	patients aged more than 80, severe left ventricular dysfunction and chronic kidney disease	213 patients	18 months	77.9%	72.9 ± 8.6 years	HT: 67.6%<br>DM: 35.7%<br>Actively Smoking: 9.4%	ACS: 52.6%<br>Stable angina: 41.3%<br>STEMI: 6.1%	RA only	Target lesion revascularisation (TLR), readmission with heart failure, acute coronary syndrome (ACS) within 1 year, 30-day mortality and 18-month mortality.	TLR: 3/213<br>Rehospitalization with MI in 1 year: 17/213<br>Rehospitalization with HF in 1 year: 5/213<br>30 day mortality: 7/213<br>18 months mortality: 28/213<br>MACE: 38/213		severe LVSD and CKD 4- 5 may be a predictor for worse outcomes among rotablation patients.

No.	First Author, Year	Study design and Period	Study Population	Sample size (n)	Follow-up (months)	Male (n,%)	Age (years)	CAD risk factors n (%)	Clinical pesentation n (%)	Comparisons	Outcomes measured	Events Rota (n/total)	Events Comparator (n/total)	Conclusions
100	Tern, 2024	R Cohort (2020-2022)	Patients who undergo rotatripsy (RA+IVL)	57	1 month	67%	Median Age 72 (mean: 63,80)	HT: 89%<br>DM: 72%<br>Actively Smoking: 23%<br>Dyslipidemia: 84%	NR	No comparison, All patients receive RA +IVL	In hospital and 30-day MACE	In-hospital<br>• MACE 10.5%<br>• MI 5.3%<br>• Mortality 5.3%<br>• TVR 0%<br>• Procedural Success 100%<br>• Stroke 5.3%<br>• Perforation 0%<br>• Slow/No-reflow 11%<br>30-day<br>• MACE 12.3%<br>• MI 5.3%<br>• Mortality 7.0%<br>• TVR 1.8%		"RotaTripsy" was successful in facilitating stent delivery and restoring flow,
101	Tervo, 2022	R Cohort (August 2014 - November 2018)	Stable CAD and ACS Patients undergoing PCI using drug-coated balloon (DCB) with paclitaxel combined with citrate ester excipient (CEE)	338	25 ± 12 months	68%	71 ± 11	HT: 70%<br>DM: 37%<br>Smoker: 14%	UAP or NSTEMI: 36%<br>STEMI: 12%	Stable CAD vs. ACS (Rotational atherectomy was used in 11% of cases before the DCB-only approach)	MACE, mortality, TLR	12 Months (only presented in percent)<br>Total mortality: 7.2 ± 1.4%<br>CVD Death: 4.8 ± 1.2%<br>MACE rate: 11.6 ± 1.8%<br>TLR Rate : 5.6 ± 1.3%<br><br>24 Months (only presented in percent)<br>Total mortality: 10.6 ± 1.8%<br>CVD Death: 5.6 ± 1.3%<br>MACE rate: 15.5 ± 1.8%<br>TLR Rate : 6.0 ± 1.3%		The DCB-only strategy using a paclitaxel-coated (CEE) device was technically feasible, safe, and effective in an all-comers population.

No.	First Author, Year	Study design and Period	Study Population	Sample size (n)	Follow-up (months)	Male (n,%)	Age (years)	CAD risk factors n (%)	Clinical pesentation n (%)	Comparisons	Outcomes measured	Events Rota (n/total)	Events Comparator (n/total)	Conclusions
102	Towashiraporn, 2023	R Cohort (January 2015 - December 2018)	Patients undrgo RA-assisted PCI and whose creatinine levels were accessible	528	12 months	46.2%	72.6 ± 9.7	NR	NR	No comparison, All patients receive RA-assisted PCI	MACCE consists of cardiac death, ischemic stroke, definite stent thrombosis, target lesion revascularization, and target vessel revascularization.	1-year MACCE:7.39%<br><br>HR for developing MACCE:<br>Previous CABG: 2.53<br>Female: 1.91<br>Increased Cr: 1.16<br><br>The cut point of serum creatinine that contributed to the escalation of the MACCE rate was 1.4 mg/dl.		Rotational atherectomy is feasible for treating calcified coronary artery lesions as this procedure provides good long-term clinical follow-up. The higher the serum creatinine level, the greater risk of developing MACCE.
103	Towashiraporn, 2022	R Cohort (January 2015 - December 2018)	Patients with heavily calcified coronary lesions who underwent RA-assisted PCI	616 patients (663 lessions)	12 months	52,60%	72.8 ± 9.7	HT: 93.5%<br>Smoking: 11.0%	STEMI: 2.6%<br>NSTE-ACS: 30.4%<br>Stabel CAD: 66.9%	No comparison, All patients receive RA-assisted PCI	MACCE (cardiac death, ischemic stroke, definite stent thrombosis, target lesion revascularization (TLR), and target vessel revascularization (TVR).	30-day MACCE<br>cardiac death: 6/660<br>Ischaemic stroke: 0/660<br>Definite stent thrombosis: 3/660<br>TLR: 2/660<br>TVR: 3/660<br><br>1-year MACCE<br>cardiac death: 10/660<br>Ischaemic stroke: 0/660<br>Definite stent thrombosis: 17/660<br>TLR: 22/660<br>TVR: 29/660		RA is an effective revascularization technique for heavily calcified lesions with high success rate and good short- to intermediate-term

No.	First Author, Year	Study design and Period	Study Population	Sample size (n)	Follow-up (months)	Male (n,%)	Age (years)	CAD risk factors n (%)	Clinical pesentation n (%)	Comparisons	Outcomes measured	Events Rota (n/total)	Events Comparator (n/total)	Conclusions
104	Watanabe, 2018	R Cohort (January 2010- March 2014)	Patients with low LVEF	272	1 month	76% (Low LVEF) 75.9% (Preserved LVEF)	69.7 ± 7 (Low LVEF) 70.8 ± 8.8 (Preserved LVEF)	HT: 88% (Low LVEF) 88.9% (Preserved LVEF) DM: 55% (Low LVEF) 51.3% (Preserved LVEF) smoking: 58% (Low LVEF) 63.9% (Preserved LVEF)	NR	All patients received RA but catgorazed based on MVEF: Low LVEF vs. Preserved LVEF	MACE (including all-cause death, MI, emergency CABG and TVR)	Low LVEF<br>MACE : 3/33<br>death :1/33<br>postprocedure onset MI: 1/33<br>emergency CABG: 0/33<br>TVR: 1/33<br><br>Preserved LVEF:<br>MACE : 3/237<br>death : 2/237<br>postprocedure onset MI: 2/237<br>emergency CABG: 0/237<br>TVR: 1/237		The angiographic success rate and in-hospital MACE rate of PCI with A in patients with low LVEF could be expected to have good outcomes similar to those for patients with preserved LVEF.
105	Wei, 2016	R Cohort (September 2011 - June 2014)	Patients with heavily calcified coronary arteries treated with RA and DES	80	12–18 months	65%	72.4 ± 10.4	HT: 86.3%<br>DM: 32.5%<br>Smoking: 40%	AMI: 13.8%<br>UA: 65%	No comparison, All patients receive RA	Primary endpoint: all-cause death.<br><br>Secondary endpoints: MACCE (cardiac death, non- fatal MI, recurrence of angina, worsening of heart failure, TVR, TLR, and non-fatal ischemic stroke).	All cause death: 5/80<br>All MACCE: 12/80<br>Cardiac death: 3/80<br>TLR:4/80<br>TVR: 1/80<br>Worsening of heart failure: 3/80<br>Recurrence of angina: 4/80<br>Ischemic stroke: 2/80		RA followed by implantation of DES was effective and safe for heavily coronary calcified patients. Diabetes, LVEF and maximum pressure of post-dilatation were predictive for MACCE.

No.	First Author, Year	Study design and Period	Study Population	Sample size (n)	Follow-up (months)	Male (n,%)	Age (years)	CAD risk factors n (%)	Clinical pesentation n (%)	Comparisons	Outcomes measured	Events Rota (n/total)	Events Comparator (n/total)	Conclusions
106	Whiteside, 2018	R Cohort	patients age greater than 75, undergoing RA over a three-year period	28 patients	6 months	60.7%	80.36 ± 4.36	HT: 100%<br>DM: 50%<br>Dyslipidemia: 75%	NR	No comparison, All patients receive RA-assisted PCI	MACE	MACE<br>Death: 2/28<br>Sustained Ventricular arythmia:1/28<br>MI STEMI:0/28<br>MI NSTEMI:2/28<br>Type 2 MI: 2/28<br>TVR: 4/28<br>Stroke: 0/28		RA is a safe and feasible technique that should be considered in elderly patients with severe CAC.
107	Whiteside, 2019	R Cohort	Patient with severely calcified lesion	20 patients	12 months	15 (75%)	66.6 ± 9.4	HT: 17 (85%)<br>DM: 12 (60%)	STEMI : 1 (5%)<br>NSTEMI : 8 (40%)<br>UA : 11 (55%)<br>Stable CAD : 0 (0%)	Rotational atherectomy (single arm)	Procedural safety outcomes and major adverse cardiac events (MACE)	**In Hospital Outcome**<br>Procedural success : 20/20<br>MACE : 0/20<br>Death : 0/20<br>MI : 0/20<br>TLR : 0/20<br>Stroke : 0/20<br><br>**30-days outcome**<br>MACE : 1/20<br>Death : 0/20<br>MI : 1/20<br>TLR : 0/20<br>Stroke : 0/20<br><br>**1-year outcome**<br>MACE : 8/20<br>Death : 1/20<br>MI : 2/20<br>TLR : 6/20<br>Stroke : 0/20<br><br>**Safety outcome**<br>Coronary dissection : 0/20<br>No-reflow : 0/20<br>Burr entrapment : 0/20		

No.	First Author, Year	Study design and Period	Study Population	Sample size (n)	Follow-up (months)	Male (n,%)	Age (years)	CAD risk factors n (%)	Clinical pesentation n (%)	Comparisons	Outcomes measured	Events Rota (n/total)	Events Comparator (n/total)	Conclusions
108	Yabushita, 2014	R Cohort (January 2005 - August 2011)	Patients with heavily calcified ULM (unprotected left main) lesions undergoing PCI with rotational atherectomy	64 patients	12 months	67.2%	71.4‡ 8.7	HT: 81.3%<br>DM: 54.7%<br>Dyslipidemia: 65.6%	not specified, all patients had severely calcified unprotected left main (ULM) lesions.	No comparison, All patients underwent PCI using Rota for de novo	MACE defined as cardiac death, myocardial infarction (MI), or requirement of target lesion revascularization (TLR) at the 1-year follow up.	Periprocedural outcome<br>- MI: 5/64<br>- Rupture: 0<br>- Death: 0<br><br>MACE:<br>Cardiac death: 6/64<br>MI: 3/64<br>TLR: 12/64		PCI using Rota for calcified ULM lesions might guarantee high procedural success and a low com-plication rate except fot HD patient
109	Yoneda, 2023	R Cohort (April 2009 - March 2020)	Patients who underwent RA during PCI (ACS and CCS patients)	198	24 months	61.2 (ACS), 73.8 (CCS)	75.8 +- 1.2(ACS), 75.2 +- 0.7 (CCS)	HT: 87.7% (ACS), 89.8% (CCS)<br>DM: 53.0% (ACS), 46.3% (CCS)<br>Smoking: 40.8 (ACS), 50.6 (CCS)<br>Dyslipidemia: 67.3 (ACS), 84.5 (CCS)	UAP: 27 (13.6%)<br>NSTEMI: 18 (9.0%) ,<br>STEMI: 4 (2.0%)	No comparison, All patients receive RA-assisted PCI, but the author devide the population into ACS and CCS	2-year MACE, which was defined as the composite of all-cause death, tar- get lesion revascularization (TLR), and spontaneous MI.	ACS Group (49)<br>MACE: 19/49<br>all cause death: 9/49<br>TLR: 9/49<br>MI: 2/49<br><br>CCS Group (149)<br>MACE: 26/149<br>all cause death: 6/149<br>TLR: 15/149<br>MI: 726/149		RA could be a feasible option to prepare severely calcified lesions for PCI even in patients with ACS.
110	Zimarino,1994	R Cohort (November 1991 - March 1993)	Patients with ostial lesions (OL) treated with RA + PTCA	63	>6 months	70%	64 ± 10	NR	Stable angina: 25%<br>Unstable angina: 44%	No comparison, All patients receive RA and followed by PTCA	Procedural success, in-hospital major complications (death, emergency CABG, AMI).<br>24 h angiography, 6 months treadmill testing and/or cardiac catheterization	Procedural success: 58/63<br>CABG: 1/58<br>MI: 1/58<br>Elevated CK-MB without MI: 7/58<br><br>6 months follow up:<br>abnormal Exercise test : 30/58<br>angiographic resetenosis in at least 1 successful treted OL: 13/30		RA with adjunctive PTCA is a safe and effective treatment of OL
